# Integrating Polygenic Risk and Ocular Phenotyping Reveals an Axial-Length–Dominant Mechanism in High and Extreme High Myopia

**DOI:** 10.1016/j.xops.2026.101194

**Published:** 2026-04-15

**Authors:** Jingwen Hui, Xuehao Cui, Qiuchen Zhao, Liyu Ren, Ying Wang, Xiaoyan Yang, Jing Wang, Quanhong Han

**Affiliations:** 1 Nankai University Optometry and Vision Sicence Insitute, Nankai University Affiliated Tianjin Eye Hospital, Tianjin, China; 2 Tianjin Key Laboratory of Ophthalmology and Visual Science, Tianjin Eye Institute, Tianjin Eye Hospital, Tianjin, China; 3 Department of Clinical Neurosciences, University of Cambridge, Cambridge, UK; 4 John van Geest Centre for Brain Repair, University of Cambridge, Cambridge, UK; 5 Department of Pathology, University of Cambridge, Cambridge, UK; 6 Cancer Research UK Cambridge Centre and Department of Oncology, University of Cambridge, Cambridge, UK

**Keywords:** High myopia, Extreme high myopia, Polygenic risk score, Axial length, Whole-genome sequencing

## Abstract

**Objective:**

To integrate polygenic risk scoring with detailed ocular phenotyping to investigate severity-related genetic architecture in high myopia (HM) and extreme HM (EHM) in a clinically deeply phenotyped Chinese cohort.

**Design:**

Hospital-based cross-sectional genetic association study.

**Participants:**

A total of 576 participants from Tianjin Eye Hospital were included, comprising 443 individuals with myopia, 105 with HM, and 28 with EHM.

**Methods:**

Genomic DNA obtained from oral swab samples underwent whole-genome sequencing at an average coverage of approximately 20×. Polygenic risk scores (PRS) were constructed using external genome-wide association study summary statistics for refractive error. Detailed ocular phenotyping included axial length (AL), spherical equivalent (SE), sphere, cylinder, and inter-eye asymmetry metrics. Associations between PRS and ocular traits were assessed using correlation analyses and multivariable linear regression models adjusted for age and sex. Severity-stratified genome-wide association analyses were further performed for HM and EHM.

**Main Outcome Measures:**

Associations of PRS with myopia severity and ocular structural phenotypes, particularly AL and SE; severity-stratified genome-wide association signals in HM and EHM.

**Results:**

Polygenic risk score increased progressively across severity groups. Higher PRS was significantly associated with longer AL and more negative SE. In multivariable models, each 1-standard deviation increase in PRS was independently associated with a 0.174-mm increase in AL and a −0.498-diopter shift in SE, but was not significantly associated with cylinder. Severity-stratified genome-wide analyses suggested partially overlapping but nonidentical signal landscapes between HM and EHM. However, findings from the EHM subgroup should be interpreted cautiously because of the limited sample size.

**Conclusions:**

Polygenic burden is associated with increasing myopia severity and appears to influence refractive status primarily through axial elongation. These findings provide additional insight into the relationship between common-variant genetic risk and structural ocular phenotypes in severe myopia.

**Financial Disclosure(s):**

Proprietary or commercial disclosure may be found in the Footnotes and Disclosures at the end of this article.

Myopia is one of the most common refractive errors worldwide and continues to rise.^1^^,^^2^ Global assessments indicate that myopia prevalence was already high between 2000 and 2010 and has since increased further across many regions.^3^ The burden is particularly severe in East Asia, where prevalence among late adolescents and young adults in some regions reaches 84% to 97%, far exceeding that in most other populations.^4^^,^^5^ In China, recent epidemiological data indicate that myopia is highly prevalent among children and adolescents, with substantial increases across educational stages. In 2020, the overall prevalence was reported to be 52.7%, rising from 35.6% in primary school students to 71.1% in junior high school students and 80.5% in senior high school students, underscoring the escalating burden during school-age development.^6^^,^^7^

Within the myopia spectrum, high myopia (HM)—commonly defined as a spherical equivalent (SE) ≤ –6.0 D—and the more severe extreme HM (EHM) (SE ≤ –10.0 D) warrant particular concern.^8^^,^^9^ The prevalence of HM shows marked regional variation, ranging from 4.5% to 38% in East Asian populations, compared with approximately 2% to 5% in Western populations, indicating a substantially higher burden of extreme myopic phenotypes and potentially distinct genetic susceptibility in East Asian, particularly Chinese, populations.^10^, ^11^, ^12^ Clinically, HM is strongly associated with sight-threatening complications.^13^ Systematic reviews have demonstrated that with each additional diopter of myopia, the risk of myopic macular degeneration, retinal detachment, posterior subcapsular cataract, and primary open-angle glaucoma increases.^3^^,^^14^ Individuals approaching the extreme end of the myopia spectrum therefore accumulate a disproportionately higher risk of structural damage, highlighting the need for earlier and more precise risk stratification and mechanistic insight.^15^^,^^16^

Genetic studies have substantially advanced the understanding of myopia, with large-scale genome-wide association studies (GWASs) identifying numerous common-variant loci associated with refractive error and myopia susceptibility.^17^ Refractive error is highly heritable, but the proportion of phenotypic variance explained by currently identified common variants remains limited, especially for severe clinical phenotypes.^18^ Several important gaps therefore remain. First, relatively few studies have examined how polygenic burden relates to detailed ocular structural traits, particularly axial length (AL) and intereye asymmetry, in clinically phenotyped Chinese cohorts.^19^ Second, the performance and interpretation of polygenic risk scores (PRSs) may vary across ancestry groups and study settings. Third, severity-stratified analyses specifically focused on HM and EHM remain limited, particularly in datasets that combine genome-wide sequencing with deep ocular phenotyping. Accordingly, the novelty of the present study lies not in claiming that the genetics of myopia are unknown but in integrating PRS, ocular biometric measures, and severity-stratified genome-wide analyses within the same Chinese clinical cohort to examine how polygenic burden relates to axial elongation across the severe myopia spectrum.^20^, ^21^, ^22^

Against this background, we analyzed a clinical cohort from Tianjin Eye Hospital in which genomic DNA obtained from oral swab samples underwent whole-genome sequencing at an average coverage of approximately 20×, integrating PRS, refractive and structural phenotypes, and severity-stratified analyses within the same cohort. This study addressed 2 key questions: whether polygenic burden increases with myopia severity in parallel with axial elongation and whether stratified genome-wide analyses of HM and EHM reveal partially shared or distinct association patterns. By jointly linking PRS, AL, clinical severity, and sequencing-based stratified analyses in a hospital-based Chinese clinical cohort, this study aimed to refine the phenotypic interpretation of genetic risk in severe myopia and to generate hypotheses for future replication and functional investigation rather than to establish immediate clinical utility.

## Methods

### Study Design and Participants

**Figure 1:**
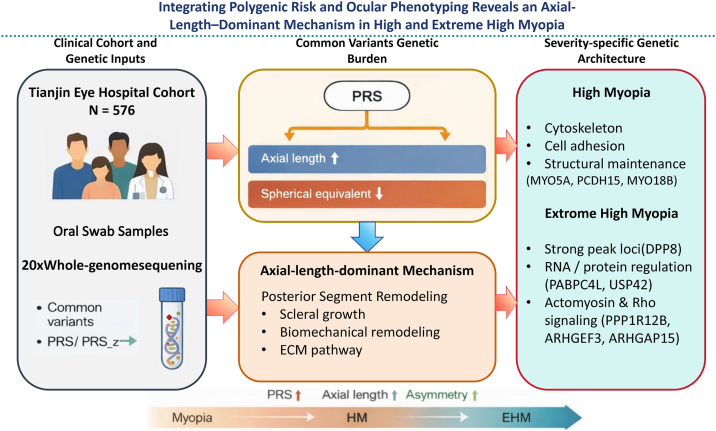
Integrated genetic framework linking polygenic risk and ocular phenotyping in HM and EHM. A Tianjin Eye Hospital cohort (N = 576) underwent whole-genome sequencing at an average coverage of approximately 20× using genomic DNA obtained from oral swab samples, together with ocular phenotyping. Polygenic risk increased with myopia severity and was associated with axial elongation and refractive deepening. Stratified analyses suggested partially overlapping yet distinct signal profiles in HM and EHM, particularly in pathways related to extracellular matrix (ECM) remodeling and structural regulation. EHM = extreme high myopia; HM = high myopia; PRS = polygenic risk score.

This study was a hospital-based genetic epidemiological investigation. Participants were recruited from patients attending Tianjin Eye Hospital (Fig 1). Oral swab samples were collected from all participants, and clinical phenotype data were obtained simultaneously, including age, sex, and bilateral refractive and AL measurements. Individuals were eligible for analysis if they had complete records of bilateral sphere, cylinder, and SE and bilateral AL measurements, ensuring the calculability of mean values and intereye asymmetry metrics. A total of 576 participants were included in the final analysis, comprising 443 individuals with myopia, 105 with HM, and 28 with EHM. Prior to genetic statistical analyses, all data underwent consistency checks and range validation, and records with missing key variables or implausible values were excluded.

### Phenotype Definition and Severity Stratification

Refractive phenotypes were defined based on sphere, cylinder, and SE, with SE calculated using the standard clinical formula:

$$\text{Spherical}\;\text{equivalent}=\text{Sphere}+\frac{\text{Cylinder}}{2}$$

To characterize both overall refractive status and intereye asymmetry, the following metrics were derived: bilateral mean measures (AL_mean, SE_mean, Sphere_mean, Cyl_mean), calculated as the arithmetic mean of right and left eyes; and intereye asymmetry measures (AL_asym, SE_asym), defined as the absolute difference between right and left eyes, reflecting asymmetry in ocular growth and refractive status. Severity stratification was based on the more severely affected eye, defined using SE_min (the minimum SE value between the 2 eyes), to avoid misclassification caused by dilution of extreme values when using bilateral averages. Consistent with commonly used thresholds in HM research, HM was defined as SE ≤ –6.00 D, and EHM was defined as SE ≤ –10.00 D. Accordingly, participants were categorized into 3 groups—myopia, HM, and EHM—using consistent SE_min-based thresholds.

### Blood Collection, DNA Extraction, Library Preparation, and Whole-Genome Sequencing

Oral swab samples were collected from all participants and preserved in cell preservation tubes (LAKEbio Co, Ltd, Cat. 3bS0107.0-01.0-50). Genomic DNA was extracted using a commercial extraction kit (Magen Biotech Co, Ltd, Cat. IVD3102-BD) according to the manufacturer’s protocol and stored at –80°C until further processing. For library preparation, 120 ng of genomic DNA was input into the experimental system (C11122, iGeneTech) to generate sequencing libraries. The quality and quantity of the resulting libraries were assessed using the Qubit dsDNA Assay Kit (Thermo Fisher Scientific) and the Agilent Bioanalyzer 2100. Only libraries meeting the predefined quality criteria, including a total library yield >100 ng and a library fragment size of approximately 500 bp, were selected for subsequent sequencing. The qualified libraries were sequenced on the SURFSeq 5000 platform (GeneMind) and the BGISEQ-T7 platform (MGI) using a 150-cycle paired-end high-output sequencing mode according to standard protocols. The SURFSeq 5000 platform is based on sequencing by synthesis, in which DNA sequences are determined by detecting fluorescent signals during real-time DNA strand extension. The BGISEQ-T7 platform is based on DNA nanoball linear amplification and combinatorial probe-anchor synthesis chemistry. The average sequencing depth across all samples was approximately 20×, and ≥96% of the whole genome in each sample was covered at a depth of ≥4×. These quality metrics supported the subsequent alignment, variant calling, and joint genotyping analyses.

### Quality Control, Alignment, and Variant Calling

Raw sequencing reads first underwent quality control to remove adapter contamination, reads with excessive low-quality bases, and reads containing a high proportion of ambiguous bases. Clean reads passing quality control were aligned to the human reference genome GRCh38 using Burrows-Wheeler Aligner maximal exact matches algorithm (BWA-MEM, v0.7.17) with default parameters. The resulting binary alignment/map files were sorted and duplicate reads were marked using Picard tools (v2.23.8) to reduce potential polymerase chain reaction amplification bias. Base quality score recalibration and variant calling were then performed using the Genome Analysis Toolkit (GATK, v4.2) following recommended best-practice procedures. Single-nucleotide variants and small insertions/deletions (indels) were identified using Genome Analysis Toolkit HaplotypeCaller, followed by joint genotyping across samples to improve consistency of variant detection within the cohort. Quality control was subsequently performed at both the sample and variant levels. At the sample level, samples with genotype call rate <95%, mean sequencing depth <10×, or evidence of abnormal heterozygosity were excluded. At the variant level, variants were filtered based on the following criteria: minor allele frequency ≥0.01, variant call rate ≥95%, and Hardy–Weinberg equilibrium *P* ≥ 1.00E-6. Variants failing these thresholds were excluded prior to downstream analyses. After these filtering procedures, the resulting high-quality variant set was used for subsequent analyses, including PRS construction and genome-wide association analyses.

### PRS

A PRS was constructed to quantify the individual-level burden of common myopia-associated alleles. Polygenic risk score construction was based on external GWAS summary statistics for refractive error from the meta-analysis reported by Tedja et al, which integrated data from the Consortium for Refractive Error and Myopia, 23andMe, and the UK Biobank Eye and Vision Consortium.^23^^,^^24^

Variants in the discovery GWAS summary statistics were harmonized with variants identified in the present sequencing dataset according to chromosome, genomic position, and effect allele. Only autosomal biallelic variants passing sample-level and variant-level quality control were retained for PRS construction. Variant inclusion criteria included a minor allele frequency ≥0.01 and genotype missingness <5%. Variants with ambiguous strand assignment or inconsistent allele orientation between the discovery and target datasets were excluded. Linkage disequilibrium (LD) clumping was performed using PLINK (A Tool Set for Whole-Genome Association and Population-Based Linkage Analyses) v1.9 with an LD threshold of r^2^ < 0.1 within a 250-kb window, retaining the most significant variant within each LD block to reduce redundancy among correlated variants.

The PRS was calculated as the weighted sum of effect alleles across retained variants: PRS = Σ (βᵢ × Gᵢ), where βᵢ denotes the effect size from the external GWAS summary statistics and Gᵢ represents the dosage of the effect allele (0, 1, or 2) carried by each participant. After quality control and LD clumping, the final PRS included approximately 120 000 variants. The resulting score was standardized to a z score (mean = 0; standard deviation [SD] = 1) to facilitate interpretation across phenotypes. To evaluate and control for potential population stratification, principal component analysis was performed using genotype data after LD pruning. In the primary regression models, age and sex were included as covariates. The top 10 principal components were additionally included in sensitivity analyses to assess the robustness of the associations to residual population structure.

### Statistical Analysis and GWAS

Continuous variables were compared across the 3 severity groups using 1-way analysis of variance, and categorical variables were compared using the χ^2^ test. Associations between PRS and ocular phenotypes were first evaluated using Pearson correlation analysis and were further quantified using multivariable linear regression models. Age and sex were included as prespecified covariates in all multivariable models.

Severity-stratified genome-wide association analyses were conducted separately for HM and EHM under an additive genetic model. Genome-wide significance was defined as *P* < 5.00E-8. To assess potential genomic inflation and overall test statistic calibration, quantile–quantile plots were generated. These analyses were used to evaluate the extent of deviation from the null distribution and to identify potential systematic bias or residual population stratification. Significant loci were annotated according to chromosomal position and nearest gene symbol to facilitate downstream biological interpretation. Given the limited size of the EHM subgroup, the stratified EHM analysis was interpreted primarily as hypothesis-generating. To further characterize shared genetic architecture, loci showing concordant or overlapping signals between HM and EHM analyses were summarized in a supplementary table.

### Ethics Statement

This study was conducted in accordance with the Declaration of Helsinki and was approved by the Ethics Committee of Tianjin Eye Hospital (Approval No. KY-2026002). Written informed consent was obtained from all participants or their legal guardians prior to enrollment.

## Results

### Cohort Composition and Baseline Phenotype Differences

A total of 576 individuals were included (myopia: n = 443; HM: n = 105; EHM: n = 28). Sex distribution was comparable across groups (male proportion: 50.56%, 45.71%, and 39.29%, respectively; Table 1). Age increased progressively with severity, from 8.21 ± 2.84 years in the myopia group to 11.31 ± 3.22 years in the HM group and 12.64 ± 4.01 years in the EHM group (*P* = 1.20E–43; Table 1), consistent with cumulative myopia progression and axial elongation, and underscoring the need for age adjustment in subsequent analyses. Refractive and structural phenotypes showed a clear severity gradient. Mean AL increased from 24.06 ± 1.12 mm to 26.09 ± 0.88 mm and 27.13 ± 1.46 mm across myopia, HM, and EHM groups (*P* = 3.76E–68; Table 1). Correspondingly, mean SE decreased from –2.04 ± 1.96 D to –6.99 ± 1.24 D and –10.84 ± 3.50 D (*P* = 1.92E–147), with similar stepwise changes observed for Sphere_mean and Cyl_mean (all *P* < 1.00E–25; Table 1). Intereye asymmetry also increased with severity. Axial length_asym rose from 0.18 ± 0.23 mm in myopia to 0.38 ± 0.37 mm in HM and 0.83 ± 1.22 mm in EHM (*P* = 1.29E–20), while SE_asym increased from 0.38 ± 0.54 D to 0.96 ± 0.85 D and 2.30 ± 3.36 D (*P* = 8.77E–23; Table 1). These findings indicate that increasing myopia severity is accompanied not only by more extreme axial and refractive phenotypes but also by greater intereye developmental asymmetry.

**Table 1:** Baseline Characteristics Stratified by Myopia Severity

Variable	Myopia	HM	EHM	*P* Value
N	443	105	28	
Age	8.21 ± 2.84	11.31 ± 4.15	12.64 ± 5.40	5.13E-23
PRS_score	13.36 ± 4.57	14.89 ± 5.07	18.02 ± 5.45	1.68E-07
PRS_z	–0.11 ± 0.95	0.21 ± 1.05	0.86 ± 1.13	1.68E-07
AL_mean	24.06 ± 1.12	26.09 ± 1.04	27.13 ± 1.64	2.60E-68
SE_mean	–2.04 ± 1.79	–6.99 ± 1.02	–10.84 ± 2.24	1.92E-147
Sphere_mean	–1.78 ± 1.74	–6.28 ± 1.13	–9.74 ± 2.26	2.25E-133
Cyl_mean	–0.77 ± 0.82	–1.51 ± 1.00	–2.30 ± 1.30	4.28E-22
AL_asym	0.18 ± 0.23	0.38 ± 0.37	0.83 ± 1.22	1.29E-20
SE_asym	0.38 ± 0.59	0.96 ± 0.95	2.30 ± 3.57	8.77E-23
Sex	Female: 218 (49.2%); Male: 225 (50.8%)	Female: 52 (49.5%); Male: 53 (50.5%)	Female: 17 (60.7%); Male: 11 (39.3%)	4.97E-1

AL = axial length; EHM = extreme high myopia; HM = high myopia; PRS = polygenic risk score; SE = spherical equivalent.

Data are presented as mean ± standard deviation or n (%). PRS_z indicates standardized polygenic risk score. AL_mean and SE_mean denote mean AL (mm) and SE (D) of both eyes, respectively. Sphere_mean and Cyl_mean represent refractive components (D). AL_asym and SE_asym indicate absolute intereye differences in AL (mm) and SE (D). *P* values were derived from 1-way analysis of variance or χ^2^ test.

### PRS Increases with Severity and Aligns with Ocular Biometry

**Figure 2:**
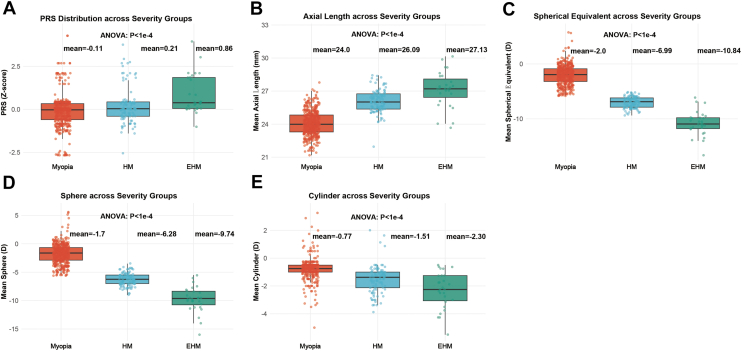
Polygenic risk score and ocular phenotypes across myopia severity groups. **A,** Distribution of standardized PRSs across myopia, HM, and EHM groups. Polygenic risk score increases stepwise with severity, with significant between-group differences (1-way ANOVA, *P* < 1E–4). **B,** Mean axial length (AL_mean) across severity groups, showing progressive axial elongation from myopia to HM and EHM (*P* < 1E–4). **C,** Mean spherical equivalent (SE_mean) across severity groups, demonstrating increasingly negative refraction with greater severity (*P* < 1E–4). **D,** Mean spherical power (Sphere_mean) across severity groups (*P* < 1E–4). **E,** Mean cylindrical power (Cyl_mean) across severity groups (*P* < 1E–4). Boxplots show medians and interquartile ranges; points represent individual participants. Group means are annotated above each panel. ANOVA = analysis of variance; EHM = extreme high myopia; HM = high myopia; PRS = polygenic risk score.

Polygenic risk scores showed a clear stepwise increase across severity groups. The raw score increased from 13.36 ± 4.57 in the myopia group to 15.20 ± 4.99 in the HM group and 17.91 ± 5.65 in the EHM group (*P* = 1.68E-7; Table 1). After standardization, the corresponding PRS values were –0.11 ± 0.95, 0.21 ± 1.04, and 0.86 ± 1.18, respectively (*P* = 1.68E-7; Table 1). These trends were visually evident in the PRS distribution plots, where both medians and overall distributions shifted rightward with increasing severity (Fig 2). Consistent stepwise changes were also observed for AL and refractive phenotypes. Taken together, these findings indicate that higher polygenic burden tracks with increasing clinical severity and key structural features of myopia.

### PRS Correlates with Axial Elongation and Refractive Error

**Figure 3:**
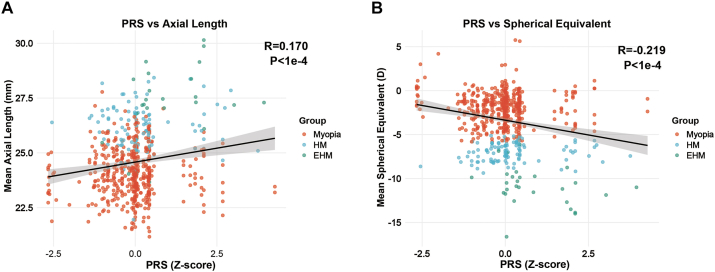
Associations between PRS and ocular phenotypes. **A,** Scatter plot showing the association between PRS and mean axial length (AL_mean). Higher PRS is associated with longer axial length (Pearson R = 0.170, *P* < 1.00E–4). **B,** Scatter plot showing the association between PRS and mean spherical equivalent (SE_mean). Higher PRS is associated with more negative refractive error (Pearson R = –0.219, *P* < 1.00E–4). Each point represents one individual, colored by severity group. Solid lines indicate linear regression fits with 95% confidence intervals. EHM = extreme high myopia; HM = high myopia; PRS = polygenic risk score.

Across the entire cohort, PRS was significantly correlated with ocular biometry and refraction. Polygenic risk scores showed a positive correlation with mean AL (Fig 3A), with a Pearson correlation coefficient of R = 0.170 (*P* < 1.00E–4), indicating that higher genetic risk is associated with longer AL. Conversely, PRS was negatively correlated with mean SE (Fig 3B), with R = –0.219 (*P* < 1.00E–4), suggesting that higher genetic risk corresponds to more negative refractive error. To improve interpretability, regression models were used to quantify phenotypic changes per SD increase in PRS. In unadjusted models, each 1-SD increase in PRS corresponded to an approximate 0.25-mm increase in AL_mean and a 0.68 D decrease in SE_mean, consistent with the observed correlations. Given the strong association between age and both AL and refraction in this cohort, subsequent analyses incorporated age and sex adjustment, after which the independent effects of PRS on key phenotypes remained evident, indicating that these associations were not solely driven by age-related differences. Additional adjustment for the top 10 principal components in sensitivity analyses did not materially change the effect estimates, indicating that the observed associations were robust to population stratification.

### Multivariable Effects Support an AL–Dominant Pathway

**Figure 4:**
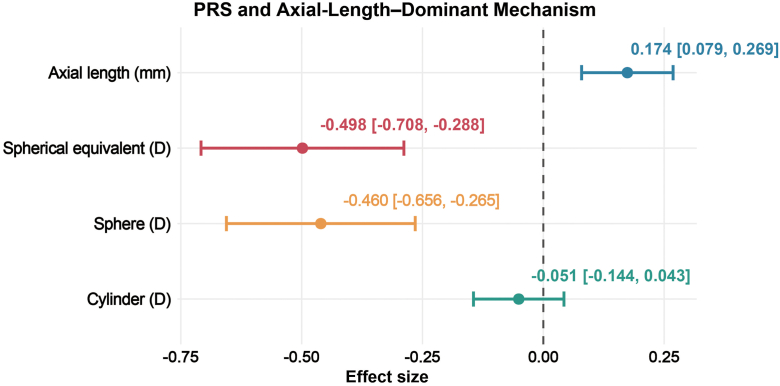
Multivariable effects of PRS on ocular phenotypes. Forest plot showing effect sizes and 95% confidence intervals for the association between PRS and key ocular phenotypes in multivariable linear regression models adjusted for age and sex. Polygenic risk score shows a significant independent effect on axial length (AL_mean) and refractive components (SE_mean and Sphere_mean), but not on cylinder (Cyl_mean). The dashed vertical line indicates the null effect (β = 0). Effect sizes represent changes in phenotype per 1-SD increase in PRS. PRS = polygenic risk score; SD = standard deviation.

In multivariable linear regression models adjusting for age and sex, the effects of PRS differed substantially across phenotypes (Fig 4). Each 1-SD increase in standardized PRS was associated with a 0.174-mm increase in AL_mean (95% confidence interval: 0.079–0.269), demonstrating an independent contribution of polygenic burden to axial elongation. Polygenic risk score was also significantly associated with refractive deepening, with effect sizes of –0.498 D for SE_mean (95% confidence interval: –0.708 to 0.288) and –0.460 D for Sphere_mean (95% confidence interval: –0.656 to 0.265). These results indicate that the genetic risk captured by PRS predominantly operates through AL–related pathways rather than astigmatism-related mechanisms.

### Correlation Structure Links Refraction, AL, and Asymmetry

**Figure 5:**
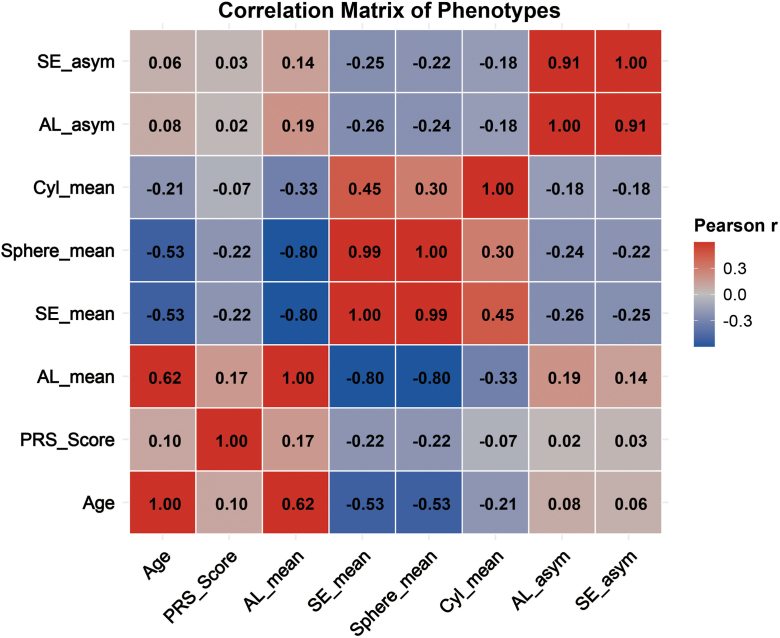
Correlation matrix of ocular phenotypes and PRS heatmap showing Pearson correlation coefficients among age, PRS, AL (AL_mean), refractive measures (SE_mean, Sphere_mean, Cyl_mean), and intereye asymmetry indices (AL_asym and SE_asym). Strong negative correlations are observed between AL_mean and refractive measures (SE_mean and Sphere_mean), while AL_asym and SE_asym show a strong positive correlation. Polygenic risk score is weakly but consistently correlated with AL and refractive phenotypes. Color scale represents Pearson r values. AL = axial length; PRS = polygenic risk score; SE = spherical equivalent.

The phenotype correlation matrix further elucidated the internal structure of ocular traits in the cohort (Fig 5). Mean AL showed strong negative correlations with both SE_mean and Sphere_mean (both approximately r = –0.80), indicating that refractive deepening is tightly coupled with axial elongation. SE_mean and Sphere_mean were almost perfectly correlated (r = 0.99), reflecting the dominant contribution of spherical power to SE. Cylinder showed only moderate correlation with SE_mean (r = 0.45) and weaker correlation with Sphere_mean (r = 0.30), suggesting a secondary role of astigmatism in determining overall refractive status. Polygenic risk scores displayed weak but consistent correlations with AL (r = 0.17) and negative correlations with SE_mean and Sphere_mean (both r = –0.22). This pattern mirrors the results shown in Figures 3 and 4 and further supports an AL–dominant genetic pathway.

Intereye asymmetry metrics were strongly linked, with AL_asym and SE_asym showing a very high positive correlation (r = 0.91), indicating that asymmetric axial growth is closely mirrored by asymmetric refractive outcomes. Age was positively correlated with AL_mean (r = 0.62) and negatively correlated with SE_mean (r = –0.53), consistent with cumulative axial elongation and myopia progression over time. Correlations between age and asymmetry measures were weaker but directionally consistent, suggesting gradual accumulation of intereye differences with growth and disease progression. Together with the marked elevation of asymmetry in HM and EHM, these findings indicate that extreme myopia may involve not only longer AL but also more asymmetric ocular growth patterns.

### Stratified GWAS Signals for HM and EHM

**Figure 6:**
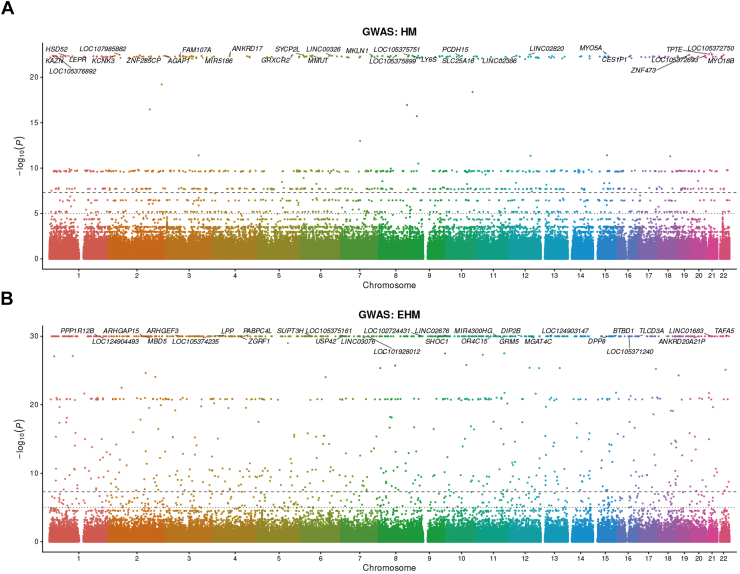
Stratified GWAS of HM and EHM. (**A**) Manhattan plot for the GWAS of HM. Each point represents a single variant plotted by chromosomal position and –log10(P). The horizontal dashed line indicates the genome-wide significance threshold (*P* = 5E–8). Top loci are annotated with nearby gene symbols. (**B**) Manhattan plot for the GWAS of EHM. Compared with HM, EHM shows stronger peak signals and a broader set of highly significant loci, including the strongest signal near DPP8. The same genome-wide significance threshold is shown. EHM = extreme high myopia; GWAS = genome-wide association study; HM = high myopia.

Genome-wide association analyses stratified by severity suggested partially overlapping but not identical signal landscapes for HM and EHM (Fig 6A, B). In the HM GWAS, 707 loci reached genome-wide significance (*P* < 5.00E–8), with *P* values ranging from 2.35E–23 to 4.99E–8 (Table S1, available at www.ophthalmologyscience.org). Top signals included loci near *MYO5A* (chr15:52329905; *P* = 2.35E–23), *TPTE* (chr21:10752117; *P* = 2.82E–23), *ANKRD17* (chr4:73171178; *P* = 3.09E–23), LINC02820 (chr12:85369026; *P* = 3.21E–23), and *PCDH15* (chr10:55450955; *P* = 3.93E–23) and loci near *MYO18B* and *SLC25A16* (Fig 6A; Table S1). These signals suggest involvement of genes related to cytoskeletal organization, cell adhesion, and regulatory noncoding regions in HM.

In the EHM GWAS, 975 loci reached genome-wide significance, with the strongest signal observed near DPP8 (chr15:65483586; *P* = 1.62E-107; Table S2, available at www.ophthalmologyscience.org). Additional highly significant loci included regions near *PABPC4L*, *USP42*, *LOC102724431*, and *LPP* and signals near *MGAT4C*, *GRM5*, *ARHGAP15*, *ARHGEF3*, and *PPP1R12B* (Fig 6B; Table S2). However, because the EHM subgroup was small (n = 28), these findings should be interpreted cautiously, and independent replication will be necessary before assigning robust biological significance to individual loci. Notably, HM and EHM shared several loci showing significant or near-significant associations in both analyses, including regions near *PLPP3*, *PDE4D*, and *RBFOX1*. These shared or concordant loci between the HM and EHM stratified analyses are summarized in Tables S3 and S4 (available at www.ophthalmologyscience.org).

### Genomic Inflation and Quantile–Quantile Plot Assessment

**Figure 7:**
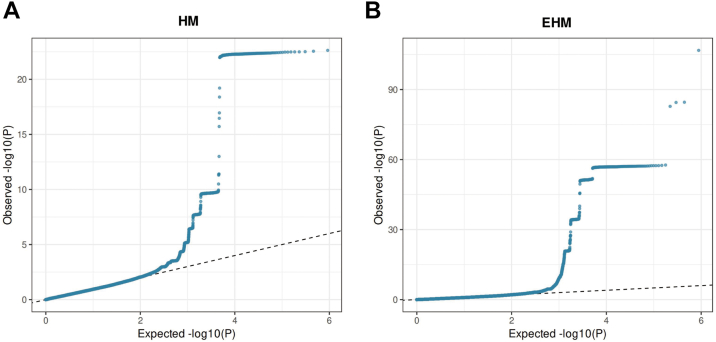
Quantile–quantile plots of GWAS results for HM and EHM. **A,** Quantile–quantile plot for the HM subgroup GWAS. **B,** Quantile–quantile plot for the EHM subgroup GWAS. The dashed line represents the expected null distribution. Observed *P* values largely follow the expected distribution, with deviation primarily in the tail region, indicating enrichment of true association signals rather than systematic inflation. EHM = extreme high myopia; GWAS = genome-wide association study; HM = high myopia.

To further evaluate the robustness of the genome-wide association results, quantile–quantile plots were generated to assess potential genomic inflation and overall test statistic calibration for both HM and EHM analyses (Fig 7). Overall, the observed test statistics showed good agreement with the expected null distribution in the early and mid portions of the distribution. Deviations from the null were primarily observed in the tail region, consistent with the presence of true polygenic association signals rather than systematic bias.

In the EHM subgroup, a more pronounced deviation in the upper tail was observed, which is expected given the smaller sample size and increased statistical variability. Importantly, there was no evidence of early global deviation, suggesting that substantial genomic inflation is unlikely to account for the observed associations.

## Discussion

In this clinically phenotyped Chinese cohort, integrated analyses of PRS, ocular biometry, and severity-stratified genome-wide signals yielded 3 main observations. First, polygenic burden increased stepwise from myopia to HM and EHM, consistent with a continuous relationship between common-variant burden and clinical severity. Second, higher PRS was independently associated with both refractive deepening and axial elongation after adjustment for age and sex, with stronger evidence for an axial-length–related pathway than for astigmatism-related mechanisms. Third, stratified genome-wide analyses suggested partially overlapping yet nonidentical signal patterns between HM and EHM. Importantly, these findings should be interpreted in the context of prior large-scale GWAS of refractive error and myopia; the contribution of the present study is not to claim that the genetics of myopia are unknown but to connect polygenic burden with detailed ocular structural phenotypes in a deeply phenotyped Chinese clinical cohort.

Evidence for an AL–dominant pathway in this study emerges from consistent findings across multiple analytic layers rather than a single statistic. The magnitude of the PRS effects observed in this study was modest, which is expected for polygenic scores derived from common variants in a highly polygenic trait such as myopia. Mean AL increased stepwise with severity and was strongly negatively correlated with SE, indicating tight coupling between refractive deepening and axial elongation. Although the PRS–AL association was modest, it remained independently significant after multivariable adjustment, suggesting that genetic effects on axial growth are not solely driven by age differences. In contrast, PRS showed no robust independent association with cylinder, and cylinder correlated only weakly with AL, indicating that astigmatism is unlikely to mediate the PRS–severity relationship. Collectively, these results support the interpretation that the genetic risk captured by this PRS primarily promotes EHM through posterior segment axial growth and tissue remodeling rather than anterior-segment astigmatic changes. Importantly, a nonsignificant association does not imply that astigmatism is clinically unimportant; rather, it suggests that the common-variant set represented by this PRS is more closely aligned with the genetic architecture of general myopia and AL. In other words, astigmatism may be driven by different genetic modules or stronger corneal determinants.^25^ Thus, to explain the larger cylinder magnitude observed in EHM, future work may require PRS models specifically optimized for astigmatism or corneal curvature rather than directly reusing a myopia-focused PRS.

A potentially underappreciated but clinically meaningful observation is that intereye asymmetry increased substantially in HM and especially in EHM. This suggests that EHM does not always develop symmetrically in both eyes; rather, one eye may undergo faster axial elongation and refractive deepening, producing an anisotropic progression pattern. Mechanistically, such asymmetry may reflect local differences in visual environment, lateralized near-work behavior, heterogeneity in ocular alignment or refractive correction adherence, or eye-specific thresholds in biomechanics and biological responses.^26^ Clinically, if EHM is more prone to asymmetric progression, screening and follow-up should not rely solely on mean values or a single refraction measurement; instead, intereye differences should be monitored to identify “rapidly progressing unilateral” trajectories earlier and to evaluate related risks such as amblyopia, strabismus, or structural fundus complications.^27^

An additional point requiring emphasis is that PRS performance remains ancestry-dependent. Recent methodological developments in polygenic risk scoring, including Bayesian approaches such as LDpred and PRS-continuous shrinkage, may further improve effect size estimation by incorporating LD structure and prior distributions. In the present study, we adopted a conventional clumping-and-thresholding framework based on large-scale meta-GWAS summary statistics, which provides computational efficiency and robust effect estimates for common variants. However, the application of more advanced Bayesian approaches may further refine PRS performance, particularly when multiomics data and larger ancestry-matched reference panels become available. Future studies incorporating such methods may help identify additional causal variants and improve predictive accuracy. Sensitivity analyses incorporating principal components further supported that the associations between PRS and ocular phenotypes were not driven by population stratification. Although PRS provides a useful summary of common-variant burden, its magnitude, calibration, and predictive performance may vary across populations because allele frequencies, LD structure, and effect-size estimates differ across ancestry groups. Our cohort consisted entirely of Chinese participants recruited from a hospital-based setting; therefore, the present findings should not be assumed to generalize directly to other populations without external validation. From a clinical perspective, PRS should currently be viewed primarily as a research and risk-enrichment tool rather than a standalone diagnostic instrument. In practice, genetic risk information is likely to be most informative when interpreted together with established clinical predictors such as baseline refraction, AL, age at onset, progression history, and environmental exposures.

The EHM-stratified GWAS identified several prominent loci, with the strongest signal located near DPP8.^28^ Additional highly significant loci included regions near PABPC4L, USP42, LOC102724431, and LPP and signals near MGAT4C, GRM5, ARHGAP15, ARHGEF3, and PPP1R12B.^29^, ^30^, ^31^, ^32^ These findings may point to biological processes related to intracellular regulation, protein homeostasis, and cytoskeletal or adhesion-related pathways. However, given the small size of the EHM subgroup, these signals should be interpreted cautiously and regarded primarily as candidates for future replication, fine-mapping, and functional validation rather than definitive mechanistic conclusions.

The HM-stratified GWAS displayed a multipeak landscape, with top loci including regions near *MYO5A* (chr15:52329905), *PCDH15* (chr10:55450955), *MYO18B* (chr22:24145378), and *ANKRD17* (chr4:73171178). A shared characteristic of these genes is their potential involvement in cytoskeletal organization and cellular structural maintenance: *MYO5A* and *MYO18B* belong to myosin-related families,^33^^,^^34^ *PCDH15* is a protocadherin-related molecule,^35^ and *ANKRD17* contains repeat domains that may contribute to the stability of structural or signaling complexes.^36^ In the context of the AL–dominant phenotype pattern observed in this study, these HM loci may represent candidate signals related to structural maintenance, mechanosensing, and ocular growth regulation, although further validation is required. High myopia loci also included multiple long noncoding RNA regions and transport-related loci (*LINC0*2820 and *SLC25A16*), suggesting that transcriptional regulation and metabolic transport processes may contribute alongside structural proteins. Because tissue-specific expression and pathway enrichment were not performed in this study, the most appropriate current interpretation is that these signals represent a set of candidate mechanisms, to be prioritized based on statistical strength, replication across strata, and consistency with AL–related phenotypic associations.

We observed partially shared significant loci between HM and EHM—for example, regions near *PLPP3*, *PDE4D*, and *RBFOX1* showed signals in both stratified analyses.^37^, ^38^, ^39^ Importantly, several loci identified in the present analyses correspond to regions previously implicated in large-scale GWAS of refractive error and myopia.^40^, ^41^, ^42^, ^43^, ^44^ For example, loci near PCDH15, PLPP3, PDE4D, and RBFOX1 have been reported in earlier GWASs, supporting the robustness and biological plausibility of these signals. In contrast, some loci showing strong signals in the EHM-specific analysis, such as those near DPP8 and USP42, have been less consistently reported in previous refractive error GWAS and may therefore represent candidate signals enriched in the most severe phenotype. Together, these findings suggest that severe myopia may share part of a common polygenic architecture with general refractive error while also potentially exhibiting additional signals associated with extreme disease severity. This supports the possibility of a shared foundational genetic “backbone” across the myopia continuum from general myopia to HM and further to EHM, potentially involving broad modules such as lipid signaling, second-messenger pathways, and neurodevelopment/splicing regulation. Taken together, these findings are compatible with the possibility that HM and EHM share part of a common genetic background, while the most extreme phenotype may additionally enrich certain signals. This interpretation remains provisional and should be tested in larger independent datasets.

### Limitations and Future Directions

This study has several limitations. First, it was based on a single-center hospital cohort, which may introduce referral-related and severity-enrichment biases. Second, the EHM subgroup was small, and therefore the extremely strong association peaks observed in the stratified EHM GWAS may reflect a combination of true signal enrichment, statistical instability, and false-positive risk; independent replication is essential before assigning biological significance to individual loci. Quantile–quantile plot assessment indicated that the observed genome-wide signals were not driven by substantial systematic inflation. The absence of early deviation from the null distribution suggests adequate control of population stratification and technical bias. However, the more pronounced tail deviation observed in the EHM subgroup likely reflects a combination of true signal enrichment and increased statistical variability due to the limited sample size, and therefore these findings should be interpreted cautiously. Third, PRS quantifies common-variant burden, but its interpretation depends on the underlying discovery GWAS and may vary across ancestry groups and clinical settings. Fourth, although whole-genome sequencing at an average coverage of approximately 20× provides broad genome-wide information, limitations remain for structural variants, highly repetitive regions, and ultrarare variants. Future work should prioritize replication in larger multicenter East Asian cohorts, refinement of ancestry-aware PRS models, and functional studies linking prioritized loci to scleral, choroidal, and retinal remodeling relevant to axial elongation.

## Conclusion

In this clinically characterized whole-genome sequencing cohort, PRS increased with myopia severity and showed consistent associations with axial elongation and refractive deepening. Multivariable analyses indicated that polygenic burden was independently associated with AL and refractive error, but not with cylinder, supporting an AL–dominant pattern in severe myopia. Stratified genome-wide analyses further suggested partially overlapping yet nonidentical signal profiles between HM and EHM, although the EHM findings require cautious interpretation and external replication. Together, these results provide an integrated framework for examining the relationship between common-variant genetic burden and structural ocular phenotypes in severe myopia.

## Data Availability

Data from the Tianjin cohort are not publicly available due to local institutional and ethical restrictions involving sensitive clinical and ophthalmic information; access to deidentified data may be granted upon reasonable request to the corresponding author, with approval from the relevant institutional review boards.
