# Supplementary material for: Integrating Polygenic Risk and Ocular Phenotyping Reveals an Axial-Length–Dominant Mechanism in High and Extreme High Myopia
Source: Ophthalmol Sci. 2026 Apr 15;6(6):101194. doi: 10.1016/j.xops.2026.101194 (PMC13195619; doi:10.1016/j.xops.2026.101194)
Supplement: Table S1 [file mmc1.pdf]

Table S1. Genome-wide significant loci for high myopia (HM)

variant.id	chr	pos	pval	gene_symbol
30782475	15	52329905	2.35E-23	MYO5A
36979025	21	10752117	2.82E-23	TPTE
9956924	4	73171178	3.09E-23	ANKRD17
27106996	12	85369026	3.21E-23	LINC02820
3746671	2	47264023	3.24E-23	LOC107985882
37139836	21	23811333	3.27E-23	LOC105372750
35829368	19	50035452	3.38E-23	ZNF473
37684576	22	25799131	3.39E-23	MYO18B
13924294	6	10897410	3.55E-23	SYCP2L
13302931	5	145858316	3.63E-23	GRXCR2
4918186	2	132326140	3.78E-23	ZNF285CP
802254	1	59157859	3.80E-23	HSD52
14514071	6	49348423	3.91E-23	MMUT
20099602	8	127088449	3.91E-23	LOC105375751
23003367	10	55450955	3.93E-23	PCDH15
32172466	16	55835677	4.07E-23	CES1P1
226415	1	13935252	4.11E-23	KAZN
7050163	3	58587474	4.11E-23	FAM107A
19455247	8	73384077	4.11E-23	LOC105375899
36785994	20	57850288	4.18E-23	LOC105372693
26337163	12	30156657	4.18E-23	LINC02386
881481	1	65658101	4.24E-23	LEPR
20318587	8	143066148	4.25E-23	LY6S
17942110	7	131472594	4.27E-23	MKLN1
413117	1	27527008	4.28E-23	LOC105376892
3459516	2	26735054	4.31E-23	KCNK3
6177449	2	235446800	4.33E-23	AGAP1
23169349	10	68540408	4.33E-23	SLC25A16
8274619	3	151650320	4.33E-23	MIR5186
15588765	6	133168410	4.36E-23	LINC00326
20698729	9	21579020	4.36E-23	MIR31HG
6323937	3	2335584	4.41E-23	CNTN4
7242535	3	72763022	4.41E-23	SHQ1
18688721	8	15689821	4.49E-23	TUSC3
7751716	3	109040829	4.50E-23	MORC1
21901842	9	123311012	4.52E-23	LOC124902266
15441100	6	120765345	4.52E-23	TBC1D32
14412067	6	41390340	4.53E-23	LOC100505711
19169448	8	50051239	4.55E-23	SNTG1
313424	1	19838266	4.59E-23	RNF186-AS1
30061400	14	91586571	4.63E-23	LOC101928957
28234278	13	47193396	4.65E-23	LOC105370195
9937050	4	71604035	4.69E-23	LOC105377271

2280876	1	185865582	4.70E-23	HMCN1
360646	1	23389487	4.70E-23	LOC124903875
21764122	9	112099015	4.71E-23	SUSD1
7752787	3	109137070	4.72E-23	C3orf85
2705743	1	220306032	4.73E-23	RAB3GAP2
18237729	7	153937437	4.76E-23	DPP6
29621922	14	56833277	4.78E-23	OTX2-AS1
10830988	4	145364310	4.78E-23	LINC02266
35116440	19	1166231	4.80E-23	LOC102723798
23931450	10	128380367	4.83E-23	LOC124902558
5504118	2	180252415	4.87E-23	LOC105373768
30932970	15	63030470	4.88E-23	TPM1
908904	1	67942538	4.88E-23	GNG12-AS1
1264252	1	97736850	4.92E-23	DPYD
14108001	6	24440581	4.92E-23	GPLD1
22401036	10	17224471	4.93E-23	VIM-AS1
15842199	6	153840960	4.94E-23	OPRM1
33998473	18	2956311	4.96E-23	LPIN2
30721764	15	47422180	4.99E-23	SEMA6D
33619291	17	59442519	5.02E-23	LOC124904040
13976240	6	15021254	5.03E-23	LOC105374945
16152506	7	2763191	5.08E-23	GNA12
7080450	3	60730960	5.09E-23	FHIT
7831698	3	115822362	5.13E-23	LSAMP
5912809	2	215087487	5.13E-23	ABCA12
37544261	22	16871609	5.13E-23	HSFY1P1
5118742	2	148658607	5.14E-23	EPC2
2460595	1	200326928	5.16E-23	LINC00862
20065212	8	124362662	5.17E-23	TMEM65
3350345	2	17857780	5.18E-23	LOC112267888
32131979	16	52788336	5.18E-23	CASC16
23990325	10	132046461	5.19E-23	JAKMIP3
17331449	7	81127272	5.21E-23	SEMA3C
661146	1	47365271	5.21E-23	CMPK1
7751584	3	109033286	5.21E-23	MORC1
8985102	4	6191913	5.21E-23	C4orf50
13691693	5	176544451	5.21E-23	CDHR2
22268236	10	8180704	5.21E-23	LOC107984206
23792240	10	118461984	5.21E-23	LOC105378507
30467375	15	27722780	5.21E-23	GABRG3
33695257	17	65950699	5.21E-23	CEP112
3231908	2	9051629	5.21E-23	LOC124907729
7380654	3	82991979	5.21E-23	LOC101927439
14118687	6	25151673	5.21E-23	CMAHP
19003346	8	38682061	5.21E-23	LOC124901936

22888192	10	46547000	5.21E-23	LOC124902419
36398718	20	29318077	5.24E-23	FRG2EP
29543724	14	50841784	5.24E-23	LOC124903423
26604021	12	43912507	5.24E-23	TMEM117
7094236	3	61746050	5.24E-23	PTPRG
1808642	1	147091270	5.24E-23	RNVU1-8
21774544	9	112896914	5.26E-23	SLC46A2
34413896	18	29198121	5.26E-23	LINC02879
10931607	4	153828840	5.27E-23	LOC101927947
6825376	3	39636998	5.27E-23	MOBP
30957146	15	64932951	5.30E-23	ANKDD1A
36750578	20	55249398	5.30E-23	LINC01440
2954246	1	239197700	5.31E-23	LOC124904566
37334608	21	38904147	5.33E-23	ETS2-AS1
27162270	12	90393575	5.34E-23	LOC124902982
4744504	2	119267247	5.35E-23	STEAP3-AS1
459316	1	31115991	5.36E-23	LOC107984935
26603715	12	43897312	5.36E-23	TMEM117
17918666	7	129589360	5.36E-23	NRF1
28892423	13	99676186	5.45E-23	CLYBL
5390501	2	171035341	5.46E-23	TLK1
20435085	9	4851885	5.49E-23	RCL1
25713309	11	118874640	5.51E-23	CXCR5
12253202	5	59128015	5.54E-23	PDE4D
2460185	1	200298575	5.55E-23	LINC00862
34568251	18	42392075	5.55E-23	LINC00907
24928077	11	57593333	5.56E-23	SERPING1
14489640	6	47457573	5.56E-23	CD2AP-DT
6445083	3	10233182	5.60E-23	IRAK2
7640546	3	100068350	5.61E-23	CMSS1
28074931	13	34325752	5.61E-23	LINC02343
35112016	19	962513	5.64E-23	ARID3A
30936597	15	63303874	5.65E-23	APH1B
32888014	17	10852559	5.66E-23	LOC124903926
7264371	3	74446683	5.66E-23	CNTN3
35613928	19	35003143	5.68E-23	GRAMD1A
4615226	2	109166459	5.69E-23	SH3RF3
3036323	1	245202759	5.70E-23	KIF26B-AS1
13998934	6	16687657	5.77E-23	ATXN1
27045195	12	80308981	5.77E-23	OTOGL
37185673	21	27321425	5.77E-23	LOC105372762
7443592	3	88049394	5.80E-23	CGGBP1
30767575	15	51194253	5.86E-23	LOC112268146
22178185	10	2616886	5.90E-23	LOC105376350
765937	1	56249737	5.90E-23	PLPP3

21538685	9	94326743	5.90E-23	NUTM2F
2830100	1	230074098	5.91E-23	GALNT2
12801849	5	105939670	5.93E-23	NA
4847559	2	127342388	5.95E-23	LOC124906074
33449430	17	45399591	5.98E-23	LOC105371796
37558050	22	17673361	5.99E-23	BCL2L13
18068320	7	141429308	6.03E-23	TMEM178B
31726063	16	14341302	6.03E-23	MIR193BHG
20903691	9	36802753	6.07E-23	LOC105376030
36000287	20	1750142	6.14E-23	LOC124904858
16498672	7	25452358	6.15E-23	LOC124901604
13667169	5	174704053	6.24E-23	MSX2
34008439	18	3638247	6.26E-23	DLGAP1
11975512	5	39261337	6.29E-23	FYB1
8545524	3	173600200	6.30E-23	NLGN1
33241222	17	28333072	6.30E-23	IFT20
19120055	8	46123768	6.30E-23	ASNSP1
28329821	13	55300825	6.31E-23	LINC02335
8001220	3	129141193	6.33E-23	ISY1
27530372	12	120298908	6.38E-23	SIRT4
34386427	18	26823438	6.39E-23	AQP4-AS1
8849741	3	196401823	6.43E-23	UBXN7
2769773	1	225367008	6.45E-23	DNAH14
10979383	4	157665341	6.46E-23	LINC02433
1845683	1	149982841	6.48E-23	OTUD7B
36011931	20	2636459	6.49E-23	TMC2
16583918	7	32282485	6.57E-23	PDE1C
1223632	1	94405310	6.59E-23	ARHGAP29-AS1
27417369	12	111336424	6.64E-23	CUX2
27481379	12	116487606	6.64E-23	LINC02457
80650	1	4398515	6.65E-23	LINC01777
17294428	7	78338984	6.66E-23	MAGI2
36846632	20	62088494	6.66E-23	LOC105372705
11985681	5	40032829	6.71E-23	LINC00603
14834242	6	72623391	6.89E-23	KCNQ5
4285205	2	88240883	7.07E-23	THNSL2
3804904	2	51456789	7.32E-23	NRXN1-DT
28176824	13	42498481	7.34E-23	LINC02341
14989041	6	84508221	7.43E-23	LOC107986620
20793099	9	28322438	7.52E-23	LINGO2
27579518	12	123786681	7.66E-23	ATP6V0A2
20610726	9	15698520	7.74E-23	CCDC171
34119207	18	11830282	7.79E-23	GNAL
8011015	3	129916960	7.83E-23	TMCC1-DT
32225405	16	59701168	8.09E-23	APOOP5

7633962	3	99450442	8.18E-23	LOC105374005
5489406	2	179069364	8.21E-23	CCDC141
24296800	11	19474129	8.28E-23	NAV2
30059762	14	91479293	8.47E-23	PPP4R3A
28770718	13	90056192	8.50E-23	LINC00559
25786479	11	124576377	8.58E-23	OR8A1
2958894	1	239576594	8.59E-23	CHRM3
7786017	3	111898182	8.76E-23	PHLDB2
31525364	16	3551981	8.83E-23	NLRC3
24085655	11	3505835	8.98E-23	LOC107984301
36125416	20	11398462	9.07E-23	LOC105372529
23782276	10	117631197	9.72E-23	LOC124902555
26886411	12	67146476	1.00E-22	LOC102724421
5207197	2	155906861	1.01E-22	LOC105373703
25484618	11	100429713	1.05E-22	CNTN5
6039812	2	225249873	6.19E-20	NYAP2
23731892	10	113443009	4.07E-19	LOC107984270
20009207	8	119671132	1.11E-17	ENPP2
5442583	2	175196845	3.47E-17	ATP5MC3
20618711	9	16249833	1.92E-16	LINC03041
17326306	7	80746718	1.01E-13	SEMA3C
30861756	15	57889216	3.91E-12	ALDH1A2
8127118	3	139462558	3.99E-12	RBP2
27150196	12	89288796	4.44E-12	LINC02458
34660537	18	49803239	5.02E-12	MYO5B
20708063	9	22247972	3.19E-11	CDKN2B-AS1
19188124	8	51473274	1.13E-10	PXDNL
1106228	1	84450716	1.33E-10	RPF1
29460637	14	44493806	1.51E-10	LINC02277
4818849	2	125114399	1.55E-10	CNTNAP5
30018906	14	88281830	1.60E-10	KCNK10
15658172	6	138992178	1.62E-10	REPS1
37911813	22	42652946	1.63E-10	CYB5R3
1205798	1	92860646	1.63E-10	DIPK1A
23556016	10	99244382	1.70E-10	HPSE2
3170146	2	4342604	1.70E-10	LOC107985841
14439176	6	43437433	1.71E-10	ABCC10
13565981	5	166976958	1.72E-10	TENM2
28745742	13	88100487	1.72E-10	LINC00373
37008679	21	14389996	1.73E-10	LOC105369304
28357693	13	57754107	1.73E-10	PCDH17
1989265	1	161559863	1.75E-10	FCGR3A
7663049	3	101985744	1.75E-10	RDUR
8798493	3	193545973	1.77E-10	ATP13A4
37869898	22	39406397	1.77E-10	TAB1

22529987	10	26558140	1.78E-10	APBB1IP
6578630	3	20706125	1.78E-10	LOC105376987
1502076	1	117126991	1.80E-10	LOC124904387
1858451	1	151038000	1.80E-10	BNIPL
7016322	3	55969392	1.82E-10	ERC2
14903520	6	77767693	1.82E-10	LOC105377865
17882126	7	126528614	1.83E-10	GRM8
35265696	19	9606676	1.84E-10	ZNF561
32399196	16	73709346	1.85E-10	ZFHX3
21796309	9	114548855	1.86E-10	ATP6V1G1
17662338	7	107598894	1.87E-10	DUS4L-BCAP29
25888989	11	132455762	1.87E-10	OPCML
9686221	4	53529102	1.88E-10	LNX1
13574664	5	167680311	1.88E-10	TENM2
4871354	2	129115110	1.88E-10	LINC01854
5610412	2	189245482	1.88E-10	COL5A2
1858847	1	151077356	1.89E-10	GABPB2
31842053	16	23258800	1.91E-10	SCNN1B
2950486	1	238924382	1.91E-10	LOC105373222
2122552	1	172239798	1.92E-10	DNM3
4654891	2	112446714	1.92E-10	RGPD8
1905837	1	154899862	1.92E-10	PMVK
10993710	4	158917720	1.92E-10	SPMIP2
5350459	2	167983477	1.93E-10	STK39
13658535	5	174144998	1.94E-10	LOC124901139
30994366	15	67788971	1.94E-10	MAP2K5
9706387	4	55142149	1.95E-10	KDR
28683898	13	83309638	1.95E-10	LOC105370286
24609079	11	43139265	1.96E-10	LOC124902662
35575421	19	32253141	1.96E-10	ZNF507
24059513	11	1894224	1.96E-10	LOC107984299
4598925	2	107904612	1.97E-10	RGPD4
3536122	2	32909975	1.97E-10	LINC00486
36029639	20	3892340	1.98E-10	PANK2
27109764	12	85648352	1.98E-10	LINC02820
36463175	20	32520285	1.98E-10	NOL4L
940638	1	70678860	1.98E-10	LINC01788
11297905	4	181754284	2.00E-10	TENM3
26532583	12	38372756	2.00E-10	ALG10B
19308188	8	61057366	2.00E-10	CLVS1
9321319	4	29312493	2.00E-10	LINC02472
18236604	7	153857322	2.01E-10	DPP6
5216558	2	156738694	2.01E-10	LINC01958
1907669	1	155076241	2.01E-10	EFNA4-EFNA3
344957	1	22152343	2.02E-10	LOC105376845

1930669	1	157008406	2.02E-10	ARHGEF11
19793809	8	101656873	2.02E-10	GRHL2
6108188	2	230613427	2.02E-10	LOC112268431
23333138	10	81326015	2.03E-10	LOC124902549
8730921	3	188394551	2.03E-10	LPP
17882366	7	126550043	2.03E-10	GRM8
33741537	17	69358569	2.03E-10	ABCA5
19192210	8	51745758	2.03E-10	PXDNL
18762653	8	19957988	2.03E-10	LPL
31865218	16	24884513	2.03E-10	TNRC6A
29532109	14	49910517	2.04E-10	ARF6
1389963	1	107722840	2.04E-10	VAV3
6544578	3	17910908	2.04E-10	TBC1D5
575118	1	40393101	2.04E-10	SMAP2
319980	1	20368264	2.05E-10	LINC01141
4461159	2	96889976	2.05E-10	FAM178B
13666859	5	174685617	2.05E-10	MSX2
36003712	20	2010639	2.05E-10	PDYN-AS1
28189263	13	43475794	2.06E-10	ENOX1
34996164	18	74767649	2.06E-10	ZNF407
33246855	17	28835024	2.07E-10	FAM222B
21389268	9	82881133	2.07E-10	LOC124902188
35356888	19	15653735	2.07E-10	CYP4F3
24698103	11	49952021	2.07E-10	OR4C13
1492722	1	116265265	2.07E-10	LOC124904669
25032701	11	66120148	2.08E-10	PACS1
22166678	10	1994025	2.08E-10	LINC00700
18696300	8	16076483	2.08E-10	MSR1
6766321	3	34666830	2.08E-10	LOC124909365
25847883	11	129388093	2.08E-10	BARX2
29262053	14	29129328	2.08E-10	LOC107984685
27097957	12	84554426	2.09E-10	LOC124903066
20608800	9	15578407	2.09E-10	CCDC171
24163901	11	8625463	2.10E-10	TRIM66
14494000	6	47789898	2.10E-10	OPN5
27456736	12	114616197	2.10E-10	LOC124903026
3409504	2	22682655	2.11E-10	LINC01830
443540	1	30025791	2.12E-10	LINC01648
37113848	21	21912500	2.12E-10	LINC01687
25797763	11	125495799	2.12E-10	FEZ1
2256163	1	183744314	2.13E-10	RGL1
12821275	5	107516174	2.13E-10	EFNA5
7254614	3	73679367	2.13E-10	PDZRN3-AS1
22042034	9	133470189	2.14E-10	LOC102723855
8467517	3	167068473	2.14E-10	LOC105374193

9860284	4	65875176	2.14E-10	LOC105377259
1932050	1	157109796	2.14E-10	ETV3L
10225388	4	95290343	2.14E-10	UNC5C
21489980	9	90564929	2.14E-10	LINC01501
29276456	14	30417746	2.14E-10	LOC112267868
15545746	6	129743053	2.15E-10	ARHGAP18
29040973	13	110603307	2.15E-10	NAXD-AS1
23722831	10	112691262	2.15E-10	VTI1A
847971	1	62896143	2.15E-10	ATG4C
13711097	5	177950034	2.15E-10	LOC124901146
3384859	2	20529383	2.15E-10	LOC102724948
6121807	2	231651402	2.15E-10	PTMA
11823817	5	27575863	2.15E-10	LOC124901177
14829483	6	72218107	2.15E-10	RIMS1
17735887	7	113869216	2.15E-10	PPP1R3A
32985828	17	17858226	2.15E-10	TOM1L2
3071736	1	247422378	2.15E-10	NLRP3
5966142	2	219369608	2.15E-10	DNPEP
30876574	15	58920627	2.15E-10	SLTM
36458581	20	32131481	2.15E-10	TM9SF4
23808666	10	119639672	2.15E-10	BAG3
13389884	5	152863985	2.15E-10	LINC01470
35773805	19	46417366	2.16E-10	CCDC8
22190614	10	3298153	2.16E-10	LOC107987146
18984115	8	37108093	2.16E-10	LOC105379377
17753936	7	115462792	2.16E-10	SNORA25B
24459810	11	31731567	2.16E-10	ELP4
33483245	17	48071995	2.16E-10	CBX1
13754223	5	180537804	2.16E-10	CNOT6
8967987	4	5163538	2.17E-10	STK32B
383972	1	25106042	2.17E-10	LOC124903880
35817829	19	49251221	2.17E-10	LOC107985340
13989429	6	15980655	2.17E-10	LOC105374949
15086669	6	92204498	2.17E-10	LOC124901505
22000175	9	130543798	2.18E-10	LOC124902286
35484972	19	23814216	2.18E-10	RPSA2
30997894	15	68043401	2.18E-10	PIAS1
10615883	4	128302749	2.18E-10	LINC02615
10615883	4	128302749	2.18E-10	LINC02615
1139506	1	87192117	2.18E-10	LINC02801
32235332	16	60439077	2.19E-10	LOC101927605
35143625	19	2594405	2.19E-10	GNG7
31719545	16	13811317	2.19E-10	LOC124903646
5966296	2	219380842	2.19E-10	DNPEP
20407727	9	3172768	2.19E-10	PUM3

37414767	21	44266469	2.19E-10	DNMT3L
28331762	13	55452747	2.20E-10	LINC02335
34144830	18	13635931	2.20E-10	LDLRAD4
4556858	2	104812152	2.20E-10	LINC01114
28196292	13	44055228	2.20E-10	NRAD1
30920432	15	62148095	2.20E-10	C2CD4B
35362374	19	15921054	2.21E-10	CYP4F11
22181725	10	2838232	2.21E-10	LOC105376352
33716787	17	67502744	2.21E-10	PITPNC1
23318682	10	80215726	2.21E-10	LINC00857
33766133	17	71336001	2.21E-10	CASC17
9299920	4	27704492	2.22E-10	LOC105374548
26768494	12	57345500	2.22E-10	R3HDM2
3174219	2	4626296	2.22E-10	LINC01249
15394369	6	117092007	2.22E-10	RFX6
10588111	4	125987232	2.22E-10	LINC02379
15545404	6	129718929	2.23E-10	ARHGAP18
15846664	6	154192318	2.24E-10	OPRM1
25481454	11	100176851	2.24E-10	CNTN5
31378769	15	96802591	2.24E-10	LOC105371002
36732945	20	53893220	2.25E-10	SUMO1P1
19834795	8	104911224	2.25E-10	ZFPM2
7936563	3	124304127	2.26E-10	KALRN
7042660	3	58003744	2.26E-10	FLNB
18545511	8	8268073	2.27E-10	LOC105379222
14750203	6	66344127	2.27E-10	LOC105377841
661150	1	47365633	2.28E-10	CMPK1
7802852	3	113201770	2.28E-10	BOC
16417488	7	19682210	2.28E-10	POLR1F
26101103	12	11674615	2.29E-10	ETV6
28989570	13	106998148	2.29E-10	NALF1
28237127	13	47439928	2.29E-10	LOC105370195
9314680	4	28807184	2.29E-10	MIR4275
27950372	13	24764888	2.30E-10	RNF17
29246340	14	27831159	2.30E-10	MIR3171HG
29733260	14	65629829	2.30E-10	FUT8
5277799	2	161978384	2.30E-10	SLC4A10
11706990	5	18636785	2.30E-10	LINC02100
3278757	2	12292005	2.30E-10	MIR3681HG
18567961	8	9497712	2.30E-10	LOC124901883
18134773	7	146881755	2.31E-10	CNTNAP2-AS1
8220481	3	147143148	2.31E-10	LOC124909494
27619389	12	126585858	2.32E-10	LOC100996671
12796228	5	105470752	2.32E-10	LOC105379110
2302639	1	187664935	2.32E-10	LINC01037

20246790	8	138339358	2.32E-10	FAM135B
34399437	18	27960019	2.33E-10	CDH2
28339409	13	56080659	2.33E-10	LOC105370214
8126401	3	139399582	2.33E-10	COPB2-DT
23635966	10	105953949	2.33E-10	LOC105378468
34761178	18	57549424	2.33E-10	FECH
37316536	21	37463203	2.34E-10	DYRK1A
8458991	3	166411247	2.34E-10	LOC124909498
27519863	12	119450868	2.34E-10	CCDC60
2769773	1	225367008	2.34E-10	DNAH14
17611702	7	103777883	2.34E-10	RELN
30460129	15	27216750	2.34E-10	GABRG3
31828430	16	22183934	2.34E-10	LOC124903666
9561776	4	47339541	2.35E-10	GABRB1
11791510	5	25160649	2.35E-10	LINC02228
3795528	2	50762122	2.35E-10	NRXN1
5540270	2	183277773	2.35E-10	LOC124906103
31489681	16	1391160	2.36E-10	UNKL
14081813	6	22792025	2.36E-10	LOC105374974
23406412	10	86975308	2.36E-10	AGAP11
25824171	11	127487039	2.37E-10	LOC107984373
33999158	18	3005676	2.37E-10	LPIN2
27525952	12	119948407	2.38E-10	LOC112268087
36174018	20	15295595	2.38E-10	MACROD2
29436358	14	42728328	2.38E-10	NA
31793298	16	19328491	2.39E-10	LINC02858
24638317	11	45498881	2.39E-10	LOC105376654
17737559	7	114028887	2.39E-10	PPP1R3A
26367950	12	32168007	2.39E-10	BICD1
6580904	3	20868232	2.39E-10	LOC105376987
288588	1	18108985	2.39E-10	IGSF21
18924394	8	31903148	2.40E-10	NRG1
18983641	8	37069736	2.40E-10	LOC105379377
14906706	6	78009120	2.41E-10	LOC105377865
5579992	2	186537565	2.41E-10	ZC3H15
8699579	3	186017205	2.42E-10	NMRAL2P
35043676	18	77899530	2.43E-10	LINC01029
2683490	1	218490857	2.43E-10	LINC02869
16543236	7	29075956	2.44E-10	CPVL
14082900	6	22867250	2.44E-10	LOC105374974
17253035	7	75457150	2.44E-10	POM121C
10525523	4	120750687	2.45E-10	PRDM5
21744162	9	110566324	2.45E-10	SVEP1
30463027	15	27393344	2.46E-10	GABRG3
23521274	10	96400357	2.46E-10	TLL2

35987721	20	771669	2.46E-10	SLC52A3
5906627	2	214591840	2.46E-10	VWC2L
10502219	4	118923888	2.47E-10	SYNPO2
4287707	2	88454873	2.47E-10	FOXI3
939711	1	70610021	2.48E-10	LINC01788
30181538	14	100311225	2.48E-10	MIR345
21945965	9	126785973	2.48E-10	ZBTB43
13549362	5	165703848	2.48E-10	LINC01938
31318207	15	92484916	2.49E-10	C15orf32
19156039	8	49038906	2.50E-10	PPDPFL
36722760	20	53193167	2.50E-10	TSHZ2
23376888	10	84697138	2.50E-10	LOC124902550
22887649	10	46522580	2.51E-10	LINC00842
27114965	12	86072026	2.51E-10	MGAT4C
2155868	1	175225095	2.51E-10	KIAA0040
27304386	12	101882107	2.51E-10	DRAM1
31456196	15	101855328	2.52E-10	OR4F13P
662085	1	47442636	2.54E-10	FOXD2
1097390	1	83667466	2.54E-10	LINC01725
34794036	18	59877755	2.55E-10	PMAIP1
13668171	5	174771848	2.55E-10	LOC105377742
2482043	1	201998230	2.58E-10	RNPEP
31581005	16	6554725	2.58E-10	RBFOX1
457862	1	31029913	2.63E-10	PUM1
10970301	4	156973790	2.63E-10	PDGFC
26918358	12	69820858	2.67E-10	MYRFL
3991053	2	65391195	2.67E-10	SPRED2
15974101	6	163525267	2.67E-10	QKI
5864470	2	211164089	2.77E-10	ERBB4
18178439	7	149969269	2.79E-10	ACTR3C
7385475	3	83428694	2.80E-10	LOC105377183
24303605	11	20023699	2.83E-10	NAV2
29731347	14	65476637	2.94E-10	FUT8
23451325	10	90653347	2.97E-10	LINC02653
29487447	14	46521511	3.24E-10	LINC00871
15481778	6	123955590	3.71E-10	NKAIN2
24496636	11	34685745	5.15E-10	LOC102723568
13950710	6	12977380	1.22E-09	PHACTR1
36376330	20	28616462	2.62E-09	FRG1CP
18694466	8	15980356	2.80E-09	MSR1
12765297	5	102989796	3.49E-09	PAM
26305890	12	27674811	4.19E-09	PPFIBP1
30704793	15	46104507	4.59E-09	LOC105370802
30999661	15	68182425	4.60E-09	PIAS1
14763262	6	67160430	5.48E-09	LOC124900229

23440940	10	89834943	5.68E-09	LINC00865
27924203	13	23099506	6.80E-09	LOC124903134
34017950	18	4368636	1.18E-08	DLGAP1
21929736	9	125566561	1.33E-08	MAPKAP1
22007120	9	131012062	1.46E-08	LAMC3
7147449	3	65570073	1.49E-08	MAGI1
559703	1	39187895	1.52E-08	MACF1
33440944	17	44746053	1.55E-08	DBF4B
12862656	5	110737002	1.59E-08	TMEM232
6126853	2	231944931	1.62E-08	DIS3L2
35553944	19	30630400	1.64E-08	ZNF536
27430059	12	112537873	1.66E-08	PTPN11
14022975	6	18446840	1.66E-08	RNF144B
10427939	4	112790484	1.67E-08	ANK2
16074739	6	169871709	1.67E-08	LOC105378149
3189090	2	5688834	1.68E-08	LOC107985843
35473364	19	23107073	1.68E-08	ZNF730
36608694	20	44608814	1.69E-08	PKIG
1387614	1	107518165	1.71E-08	NTNG1
8477602	3	167939643	1.72E-08	LRRC77P
2786627	1	226721348	1.73E-08	ITPKB
23613865	10	104079926	1.73E-08	COL17A1
27620729	12	126679915	1.73E-08	LINC00944
2906498	1	235904465	1.73E-08	LOC105373215
33841397	17	76583601	1.73E-08	ST6GALNAC2
31564540	16	5762356	1.74E-08	RBFOX1
18078190	7	142250256	1.74E-08	TAS2R38
37709031	22	27522876	1.75E-08	LOC105372981
29629264	14	57398643	1.75E-08	NAA30
31929830	16	29669876	1.76E-08	SPN
30908293	15	61183533	1.76E-08	RORA
20376982	9	1319700	1.76E-08	LOC102723803
13842464	6	5090015	1.76E-08	LYRM4
34778192	18	58797050	1.77E-08	LINC01926
18660384	8	14231160	1.77E-08	SGCZ
7913131	3	122338499	1.77E-08	CSTA
10485408	4	117552561	1.77E-08	LINC02263
10051108	4	80974687	1.77E-08	CFAP299
428933	1	28873271	1.78E-08	OPRD1
19174041	8	50395506	1.78E-08	SNTG1
18255932	7	155073323	1.79E-08	HTR5A
16383158	7	17208251	1.79E-08	AHR
34149637	18	13956929	1.80E-08	MC2R
36099079	20	9222036	1.80E-08	PLCB4
10712376	4	135730641	1.80E-08	LINC00613

12263333	5	59979421	1.80E-08	PDE4D
16029150	6	167338824	1.80E-08	TTLL2
13215399	5	138509894	1.81E-08	ETF1
5469225	2	177424452	1.81E-08	AGPS
5216558	2	156738694	1.82E-08	LINC01958
23634854	10	105868682	1.82E-08	LINC02627
13572134	5	167474519	1.82E-08	TENM2
10817671	4	144114460	1.82E-08	GYPA
23481380	10	93195780	1.82E-08	MYOF
31600818	16	7234588	1.82E-08	RBFOX1
13952870	6	13179405	1.83E-08	PHACTR1
20445732	9	5573226	1.83E-08	LOC124902114
7841404	3	116642645	1.83E-08	LSAMP
36846413	20	62074317	1.83E-08	LOC105372705
973128	1	73573094	1.84E-08	LOC105378802
12019633	5	42755007	1.84E-08	CCDC152
23918251	10	127455700	1.85E-08	DOCK1
5261241	2	160498239	1.85E-08	RBMS1
27639336	12	127986804	1.85E-08	LINC00508
6242005	2	239784371	1.85E-08	NDUFA10
21803578	9	115098938	1.86E-08	DELEC1
17666747	7	107942001	1.86E-08	LAMB1
29654180	14	59397492	1.87E-08	DAAM1
27204876	12	94023177	1.87E-08	LOC105369912
35358202	19	15684756	1.88E-08	CYP4F12
12199811	5	54813635	1.88E-08	LOC124900976
2101885	1	170488827	1.88E-08	GORAB-AS1
18561667	8	9171782	1.88E-08	LOC124901882
31044529	15	71779846	1.89E-08	THSD4
3557580	2	34294865	1.89E-08	LINC01320
14888407	6	76706828	1.89E-08	LOC105377861
8535695	3	172716546	1.89E-08	NCEH1
31523593	16	3442674	1.89E-08	NAA60
10109088	4	85831900	1.89E-08	ARHGAP24
14027420	6	18763593	1.89E-08	LOC105374957
31208880	15	84938200	1.90E-08	SLC28A1
25517766	11	102959682	1.90E-08	MMP13
24692991	11	49690793	1.90E-08	OR4C13
27727594	12	133029821	1.90E-08	ZNF84-DT
7007745	3	55259399	1.91E-08	LOC124906243
31906579	16	27986505	1.91E-08	GSG1L
31669782	16	10953976	1.91E-08	CLEC16A
21825074	9	116805109	1.91E-08	ASTN2
4997573	2	138675955	1.91E-08	NXPH2
37156518	21	24974688	1.92E-08	LINC01692

8045460	3	132504310	1.92E-08	DNAJC13
25499189	11	101558398	1.92E-08	TRPC6
16545551	7	29259023	1.93E-08	CHN2
33823356	17	75321169	1.93E-08	GRB2
2379297	1	193711617	1.93E-08	LOC124904475
2756942	1	224335758	1.93E-08	NVL
2841935	1	230929322	1.93E-08	TTC13
4756491	2	120301189	1.93E-08	LOC105373584
5416335	2	173008069	1.93E-08	RAPGEF4
10424975	4	112546072	1.93E-08	ZGRF1
11800975	5	25894167	1.93E-08	LOC124901176
13514514	5	162888391	1.93E-08	LOC105377698
20532445	9	10722854	1.93E-08	PTPRD-DT
24341215	11	22745118	1.93E-08	GAS2
27156062	12	89813876	1.93E-08	LOC105369890
27945091	13	24448456	1.93E-08	TPTE2P6
30663022	15	42625647	1.93E-08	STARD9
36441135	20	30940606	1.93E-08	ANKRD20A21P
23154650	10	67430820	1.93E-08	CTNNA3
400863	1	26512374	1.93E-08	HMGN2
9832996	4	63946058	1.93E-08	LOC105377254
7268033	3	74729207	1.93E-08	LOC105377166
25677849	11	116271687	1.93E-08	LOC107987166
14492216	6	47660958	1.94E-08	ADGRF2P
28173780	13	42258267	1.94E-08	DGKH
7780306	3	111434220	1.94E-08	LOC105374039
15919283	6	159622507	1.94E-08	LOC105378085
1108159	1	84621264	1.94E-08	LINC01461
14970521	6	82826247	1.94E-08	LOC105377877
20376537	9	1288745	1.94E-08	LOC102723803
21236664	9	70505587	1.94E-08	KLF9-DT
31532890	16	4018951	1.94E-08	ADCY9
35730304	19	43299551	1.94E-08	LINC03078
34138220	18	13142189	1.94E-08	CEP192
27688153	12	131016892	1.94E-08	ADGRD1
28656658	13	81179522	1.95E-08	LINC00564
17724650	7	112907842	1.95E-08	SAMTOR
11194388	4	174215969	1.95E-08	LINC02268
9793909	4	61220112	1.95E-08	ADGRL3
26965018	12	73611996	1.95E-08	LINC02445
22289593	10	9741219	1.95E-08	LINC02663
37498173	22	12414089	1.96E-08	LOC107987323
25269233	11	84124847	1.96E-08	DLG2
26276847	12	25376660	1.96E-08	LMNTD1
35586623	19	33031210	1.96E-08	RHPN2

24555060	11	39155842	1.96E-08	LOC101928563
13206502	5	137692819	1.96E-08	KLHL3
8106534	3	137613717	1.96E-08	LOC105374126
29769747	14	68648674	1.96E-08	RAD51B
27674517	12	130174893	1.97E-08	FZD10
18710457	8	16905015	1.97E-08	LOC105379297
3475460	2	28211386	1.97E-08	BABAM2
17940681	7	131325702	1.98E-08	MKLN1
12374166	5	69390952	1.98E-08	CENPH
15255846	6	105891626	1.99E-08	LOC105377923
4750433	2	119765914	1.99E-08	PTPN4
25712122	11	118789072	1.99E-08	DDX6
5072898	2	144632395	1.99E-08	LINC01412
27855673	13	18596426	1.99E-08	LINC00349
2001107	1	162462617	1.99E-08	UHMK1
21541652	9	94528666	1.99E-08	LOC107987100
28411116	13	61949708	2.00E-08	LINC00358
22285317	10	9370000	2.00E-08	LOC101928272
1239291	1	95689022	2.00E-08	LOC101928219
25206190	11	79181951	2.00E-08	TENM4
12005124	5	41523428	2.01E-08	PLCXD3
26989278	12	75532059	2.01E-08	LOC105369844
11212901	4	175731966	2.01E-08	GPM6A
19256384	8	56863442	2.01E-08	BPNT2
5296229	2	163601218	2.01E-08	FIGN
35945795	19	56393140	2.01E-08	ZNF582
26549179	12	39733069	2.01E-08	REDIC1
8192259	3	144930362	2.02E-08	LOC105374141
4031822	2	68441012	2.02E-08	FBXO48
22214431	10	4873685	2.02E-08	AKR1E2
23919065	10	127501826	2.03E-08	NPS
11190594	4	173928821	2.03E-08	LOC105377543
37050675	21	17434919	2.04E-08	LINC01549
13231001	5	139890473	2.04E-08	NRG2
25551141	11	105623053	2.04E-08	GRIA4
22470043	10	21883251	2.04E-08	DNAJC1
25945425	12	1058789	2.04E-08	ERC1
32223341	16	59546383	2.04E-08	APOOP5
28793998	13	91882670	2.05E-08	GPC5
35432628	19	20620846	2.05E-08	ZNF626
23480403	10	93120796	2.05E-08	CYP26A1
14520373	6	49845707	2.05E-08	CRISP1
22982570	10	53957503	2.05E-08	PCDH15
13575840	5	167776161	2.07E-08	TENM2
28477034	13	66970644	2.07E-08	PCDH9

4522525	2	101962640	2.07E-08	LINC01127
13310478	5	146472701	2.08E-08	TCERG1
21420048	9	85311131	2.08E-08	LOC124902322
11133543	4	169230855	2.09E-08	SH3RF1
15790230	6	150044310	2.10E-08	ULBP3
549622	1	38377799	2.11E-08	LOC105378657
24208463	11	12067488	2.11E-08	LINC02547
26776185	12	58030428	2.12E-08	LOC105369784
7365840	3	81749005	2.13E-08	GBE1
23261945	10	75981850	2.13E-08	LRMDA
30928306	15	62720648	2.14E-08	TLN2
21420112	9	85314566	2.15E-08	LOC124902322
13748784	5	180233038	2.16E-08	MAPK9
33840034	17	76498789	2.19E-08	RHBDF2
32464159	16	77920927	2.19E-08	VAT1L
16768836	7	46731541	2.20E-08	LOC730338
29786776	14	70110673	2.21E-08	SLC8A3
28645874	13	80330884	2.21E-08	SPRY2
22670898	10	36753220	2.22E-08	LOC124900298
28854642	13	96765588	2.22E-08	HS6ST3
37680739	22	25512691	2.26E-08	CRYBB2P1
12336264	5	66338634	2.27E-08	LOC124900988
17816792	7	121167725	2.29E-08	CPED1
18917038	8	31292730	2.30E-08	LOC101929492
20065429	8	124381213	2.30E-08	TMEM65
8504407	3	170163461	2.31E-08	PHC3
12249773	5	58860218	2.35E-08	RAB3C
23860072	10	123446067	2.40E-08	LINC02641
3188818	2	5667934	2.56E-08	LOC107985843
29760311	14	67786262	2.57E-08	ZFYVE26
35354592	19	15543858	2.76E-08	CYP4F22
141293	1	8485796	3.05E-08	RERE
4717427	2	117246694	3.56E-08	NA
27214656	12	94776001	3.68E-08	KRT19P2
