# Supplementary material for: Integrating Polygenic Risk and Ocular Phenotyping Reveals an Axial-Length–Dominant Mechanism in High and Extreme High Myopia
Source: Ophthalmol Sci. 2026 Apr 15;6(6):101194. doi: 10.1016/j.xops.2026.101194 (PMC13195619; doi:10.1016/j.xops.2026.101194)
Supplement: Table S2 [file mmc2.pdf]

Table S2. Genome-wide significant loci for extreme high myopia (EHM)

variant.id	chr	pos	pval	gene_symbol
30964596	15	65483586	1.62E-107	DPP8
10691430	4	134050880	2.70E-85	PABPC4L
16214432	7	6083223	3.42E-85	USP42
20949080	9	40196897	1.58E-83	LOC102724431
8732405	3	188512558	2.17E-58	LPP
2538660	1	206501638	3.05E-58	LOC124904493
36437470	20	30812772	3.96E-58	ANKRD20A21P
2487541	1	202420967	4.13E-58	PPP1R12B
27117979	12	86362866	4.13E-58	MGAT4C
25233633	11	81373154	4.36E-58	MIR4300HG
31186132	15	83051877	5.40E-58	BTBD1
22281076	10	9032227	5.74E-58	LINC02676
28034240	13	31007100	6.39E-58	LOC124903147
32081576	16	48720970	6.40E-58	LOC105371240
14462150	6	45234530	6.49E-58	SUPT3H
5060783	2	143555860	6.59E-58	ARHGAP15
7030583	3	57043490	6.61E-58	ARHGEF3
37084678	21	19923228	6.63E-58	LINC01683
38007868	22	48616282	6.81E-58	TAFA5
25330619	11	88787131	6.83E-58	GRM5
16332521	7	13718499	7.58E-58	LOC105375161
17721158	7	112579145	7.60E-58	LINC03076
17721682	7	112621346	7.70E-58	LOC101928012
26685762	12	50652346	7.71E-58	DIP2B
24896508	11	55473867	7.77E-58	OR4C15
21759785	9	111793679	7.85E-58	SHOC1
32728901	17	672175	7.98E-58	TLCD3A
10425264	4	112573167	8.01E-58	ZGRF1
8606165	3	178280786	8.04E-58	LOC105374235
5116481	2	148464801	8.18E-58	MBD5
34502939	18	36794832	8.30E-58	TPGS2
2410409	1	196074066	8.60E-58	LINC01724
31734838	16	15095861	8.62E-58	MIR6511B2
13559785	5	166530848	8.66E-58	LOC105377705
2095333	1	169940911	8.73E-58	KIFAP3
31581005	16	6554725	8.73E-58	RBFOX1
33682003	17	64941035	8.76E-58	LOC107985000
31049891	15	72234089	8.82E-58	PKM
1050816	1	80146080	8.88E-58	LINC01781
23376888	10	84697138	9.03E-58	LOC124902550
10511875	4	119632087	9.17E-58	PDE5A
19188124	8	51473274	9.22E-58	PXDNL
4193426	2	80881111	9.25E-58	CTNNA2

35626307	19	35865173	9.48E-58	KIRREL2
8120523	3	138885133	9.90E-58	LOC124906289
1419906	1	110281555	1.02E-57	KCNC4
6713141	3	30526806	1.05E-57	LINC01985
5746845	2	201165241	1.08E-57	CFLAR
15995229	6	165025799	1.08E-57	MEAT6
24186798	11	10363491	1.10E-57	AMPD3
17765425	7	116476998	1.10E-57	CAV2
37642515	22	23202721	1.19E-57	BCR
36437474	20	30812844	1.21E-57	ANKRD20A21P
2799340	1	227587251	1.23E-57	ZNF678
3551218	2	33906683	1.24E-57	LINC01317
9325733	4	29655168	1.24E-57	LOC105374562
20854450	9	32769927	1.24E-57	LOC105376017
22272335	10	8432477	1.24E-57	LOC105376396
22677887	10	37287479	1.24E-57	ANKRD30A
37098164	21	20770757	1.24E-57	LINC00320
37585931	22	19618033	1.24E-57	LINC00895
37097671	21	20741368	1.24E-57	LINC00320
28865074	13	97703548	1.24E-57	LOC105370324
28559491	13	73257593	1.24E-57	LINC00393
33508169	17	50031758	1.24E-57	PICART1
37646048	22	23413217	1.26E-57	LINC02556
353310	1	22765546	1.26E-57	EPHB2
36437472	20	30812791	1.26E-57	ANKRD20A21P
10000568	4	76659032	1.26E-57	SHROOM3
19913159	8	111595734	1.27E-57	LOC105375707
34952017	18	71695122	1.27E-57	LINC01899
23165853	10	68289663	1.29E-57	PBLD
7734114	3	107499838	1.29E-57	BBX
29810992	14	72036200	1.30E-57	RGS6
37147387	21	24282053	1.30E-57	LINC01689
28171159	13	42061988	1.30E-57	DGKH
9684250	4	53376255	1.31E-57	LOC105377654
5138818	2	150383810	1.31E-57	LINC01818
14102381	6	24077506	1.31E-57	NRSN1
5109590	2	147890341	1.31E-57	ACVR2A
3746671	2	47264023	1.34E-57	LOC107985882
28996846	13	107569369	1.34E-57	NALF1
5377383	2	170045964	1.35E-57	UBR3
9701741	4	54760589	1.35E-57	LOC105377657
29266262	14	29502842	1.35E-57	PRKD1
840337	1	62236593	1.36E-57	KANK4
6949071	3	50280177	1.36E-57	LSMEM2
14985840	6	84233090	1.36E-57	CEP162

2632039	1	214272751	1.37E-57	LOC124904509
1080155	1	82302504	1.38E-57	LOC124904204
4237176	2	84453421	1.38E-57	SUCLG1
25649949	11	113976571	1.40E-57	HTR3A
21263196	9	72666886	1.40E-57	TMC1
36972722	21	10595909	1.41E-57	TPTE
16438135	7	21088249	1.42E-57	LINC01162
23705833	10	111368497	1.42E-57	LOC105378485
32114096	16	51347101	1.42E-57	LOC107984886
10471981	4	116484654	1.43E-57	LOC105377384
37823862	22	36128146	1.43E-57	APOL3
10107383	4	85685632	1.43E-57	ARHGAP24
26277151	12	25395697	1.43E-57	LMNTD1
28884636	13	99088692	1.44E-57	DOCK9-DT
21939794	9	126330304	1.45E-57	MVB12B
834496	1	61828401	1.46E-57	PATJ
17720843	7	112552670	1.46E-57	LINC03076
27168451	12	90904603	1.47E-57	CCER1
23710225	10	111668203	1.47E-57	LOC105378485
3131640	2	1881355	1.47E-57	MYT1L
34913352	18	68815677	1.47E-57	CCDC102B
19027113	8	40545950	1.48E-57	ZMAT4
16242433	7	7685314	1.49E-57	UMAD1
36156419	20	13827934	1.50E-57	NDUFAF5
10371842	4	108214659	1.52E-57	LEF1-AS1
28034734	13	31041515	1.56E-57	LOC124903147
25061869	11	68198437	1.57E-57	KMT5B
29944221	14	82624934	1.57E-57	LINC02301
11541535	5	6178849	1.58E-57	LOC124900933
1514616	1	118201785	1.58E-57	LOC105378930
15751732	6	147050779	1.58E-57	STXBP5-AS1
6935606	3	49090628	1.59E-57	QRICH1
23253624	10	75237120	1.59E-57	COMTD1
9690365	4	53843580	1.62E-57	PDGFRA
10483213	4	117366257	1.65E-57	LINC02263
9702238	4	54804106	1.66E-57	LOC105377657
35248706	19	8582668	1.66E-57	ADAMTS10
9889951	4	68260549	1.67E-57	TMPRSS11B
9075263	4	10916264	1.70E-57	LINC02498
10869111	4	148540538	1.70E-57	LOC124900798
10085753	4	83795049	1.71E-57	LOC105377315
2171882	1	176576474	1.74E-57	PAPPA2
8121077	3	138920559	1.79E-57	LOC124906289
22013183	9	131426337	1.81E-57	PRRC2B
13145475	5	132505971	1.83E-57	IRF1

33742079	17	69399703	1.89E-57	MAP2K6
19319984	8	62012201	1.89E-57	LINC02842
16210650	7	5884714	1.91E-57	OCM
15258333	6	106107677	1.93E-57	ATG5
7592275	3	96136503	1.93E-57	MIR8060
2047585	1	166092032	1.97E-57	FAM78B
35936219	19	55891504	1.98E-57	NLRP13
7976537	3	127128836	2.00E-57	PRR23E
10030883	4	79155019	2.06E-57	LINC01088
4847559	2	127342388	2.08E-57	LOC124906074
455562	1	30858944	2.08E-57	LOC400748
22412397	10	17991125	2.11E-57	SLC39A12
31941710	16	30749204	2.14E-57	PHKG2
25424999	11	96177916	2.14E-57	MAML2
11335693	4	184270771	2.15E-57	LOC105377584
2438418	1	198417749	2.16E-57	LOC105371677
33514101	17	50509536	2.19E-57	MYCBPAP
21713380	9	108211076	2.20E-57	LOC105376214
17943821	7	131597789	2.21E-57	PODXL
13223614	5	139227263	2.22E-57	LOC124901081
27910809	13	22268459	2.23E-57	LOC105370108
13565981	5	166976958	2.30E-57	TENM2
17290648	7	78069539	2.31E-57	MAGI2
8615105	3	179011959	2.39E-57	LOC124906307
21133572	9	62907310	2.54E-57	FAM88B
7430785	3	87053239	2.57E-57	LINC00506
32723397	17	415320	2.61E-57	LOC124903891
2347514	1	191066732	2.74E-57	LINC01680
21784254	9	113653315	2.81E-57	LOC105376223
27062733	12	81762269	2.90E-57	PPFIA2
6670850	3	27238746	3.01E-57	NEK10
18648421	8	13702526	3.08E-57	LOC102725080
35953183	19	56965482	3.12E-57	LOC124904779
10215657	4	94511598	3.12E-57	PDLIM5
27231342	12	95946059	3.51E-57	AMDHD1
29609979	14	55959968	3.66E-57	LOC102723670
1270268	1	98269490	3.86E-57	LINC01776
8679838	3	184520555	4.58E-57	LINC01840
35363375	19	15957222	4.75E-57	OR10H4
10993989	4	158940559	6.03E-57	SPMIP2
5150077	2	151305978	1.34E-52	NMI
29682074	14	61744860	2.16E-52	SNAPC1
34795849	18	60013538	2.39E-52	LOC105372151
8171606	3	143180746	2.44E-52	LOC105374138
11267191	4	179695543	2.62E-52	LOC124900819

31166055	15	81548204	2.66E-52	LOC124903541
23171274	10	68671537	2.67E-52	TET1
29327834	14	34371097	2.69E-52	LOC102724945
12236505	5	57849890	2.73E-52	LINC02225
18851837	8	26346117	2.78E-52	PPP2R2A
9990681	4	75868315	2.84E-52	PPEF2
28529547	13	71021094	2.95E-52	LINC00348
14377447	6	38734283	2.98E-52	DNAH8
22947514	10	51334152	2.99E-52	PRKG1
29244804	14	27711681	2.99E-52	MIR3171HG
29570725	14	52896999	3.02E-52	FERMT2
32100859	16	50255402	3.03E-52	ADCY7
32404095	16	74082528	3.03E-52	PSMD7-DT
12263333	5	59979421	3.24E-52	PDE4D
10450266	4	114647356	3.28E-52	UGT8
8319759	3	155351764	3.29E-52	PLCH1
272191	1	16971210	3.29E-52	CROCC
157517	1	9649101	3.31E-52	PIK3CD
33341829	17	36656103	3.33E-52	LOC105371750
35177723	19	4485883	3.38E-52	HDGFL2
21394789	9	83351323	3.44E-52	FRMD3
13631697	5	172153288	3.57E-52	STK10
6542363	3	17721459	3.57E-52	TBC1D5
33572029	17	55440842	3.59E-52	SMIM36
23011551	10	56055835	3.60E-52	ZWINT
18515639	8	6912547	3.77E-52	LOC101928095
36581200	20	42489008	4.14E-52	PTPRT
24633339	11	45060307	4.15E-52	LOC105376650
31142319	15	79650195	4.15E-52	LOC105370918
23919065	10	127501826	4.24E-52	NPS
29819330	14	72661854	4.29E-52	DPF3
8246621	3	149345435	4.35E-52	TM4SF1-AS1
12805212	5	106272062	4.42E-52	NA
5540270	2	183277773	4.43E-52	LOC124906103
16463484	7	22821405	4.55E-52	TOMM7
27135241	12	87796623	4.58E-52	LINC02258
19925604	8	112630942	4.60E-52	CSMD3
26860032	12	64822675	4.62E-52	TBC1D30
22274313	10	8546841	4.67E-52	LOC105376399
24165843	11	8801689	4.76E-52	DENND2B
4722894	2	117630619	4.76E-52	LOC124907878
5489654	2	179086986	4.76E-52	SESTD1
5698420	2	196702343	4.76E-52	CCDC150
24645926	11	46169560	4.76E-52	LOC101928894
25760596	11	122700497	4.76E-52	UBASH3B

26042566	12	7917439	4.76E-52	SLC2A3
31005913	15	68660976	4.76E-52	CORO2B
31122960	15	78312984	4.76E-52	SKIC8
32577633	16	83551056	4.76E-52	CDH13
22981362	10	53876198	4.80E-52	PCDH15
2055307	1	166672759	4.83E-52	FMO9P
7933761	3	124054357	4.83E-52	KALRN
29608305	14	55838660	4.88E-52	LOC102723670
31681199	16	11635034	4.88E-52	LITAF
32061300	16	46681032	4.88E-52	VPS35
26058567	12	8886010	4.90E-52	A2ML1
4260295	2	86375337	4.92E-52	LOC124900517
23727253	10	113073099	4.93E-52	TCF7L2
6055436	2	226613738	4.95E-52	MIR5702
15014561	6	86408185	4.97E-52	LOC101928842
2315315	1	188593566	4.97E-52	LINC01035
18410251	8	3101055	4.97E-52	CSMD1
33305496	17	33840799	4.97E-52	ASIC2
4730751	2	118195476	4.98E-52	LOC105373578
29354975	14	36391429	5.00E-52	MBIP
26137817	12	14408116	5.01E-52	ATF7IP
21501218	9	91398789	5.07E-52	NFIL3
8360155	3	158730365	5.09E-52	RARRES1
14763503	6	67176321	5.12E-52	LOC124900229
22348393	10	13669057	5.16E-52	FRMD4A
29007498	13	108351494	5.18E-52	LOC105370355
21727370	9	109262452	5.18E-52	EPB41L4B
169925	1	10555642	5.19E-52	PEX14
322651	1	20584384	5.27E-52	CDA
2018665	1	163794192	5.30E-52	LOC124904447
7811183	3	113911173	5.33E-52	GRAMD1C
32583466	16	83864980	5.34E-52	MLYCD
14024197	6	18533517	5.39E-52	MIR548A1HG
13854661	6	6011936	5.39E-52	NRN1
33973630	18	1059759	5.43E-52	LOC105371953
28874366	13	98369353	5.44E-52	LOC105370327
2912932	1	236344559	5.48E-52	EDARADD
28546451	13	72305420	5.51E-52	LOC124900345
6055919	2	226654394	5.56E-52	MIR5702
9968944	4	74189485	5.58E-52	MTHFD2L
21971950	9	128776887	5.59E-52	LOC124902282
27644201	12	128269347	5.60E-52	TMEM132C
36980849	21	10810574	5.62E-52	TPTE
22219904	10	5202939	5.63E-52	AKR1C4
12956303	5	117565686	5.68E-52	LINC00992

28469122	13	66299541	5.71E-52	PCDH9
5507570	2	180543760	5.72E-52	SCHLAP1
33731100	17	68499368	5.75E-52	PRKAR1A
15138182	6	96286372	5.82E-52	UFL1-AS1
20238310	8	137720280	5.86E-52	LOC401478
16585416	7	32374256	5.96E-52	PDE1C
33493030	17	48888313	6.03E-52	LOC105371814
34573148	18	42759712	6.16E-52	RIT2
8071470	3	134665652	6.27E-52	CEP63
13544557	5	165341020	6.31E-52	LINC01938
28500954	13	68920683	6.38E-52	LINC02342
13121696	5	130258668	6.38E-52	CHSY3
7598932	3	96644269	6.44E-52	LOC124909397
18029855	7	138431008	6.49E-52	TRIM24
4622891	2	109719027	6.61E-52	LOC107985774
27938608	13	24072825	6.83E-52	SPATA13
24538278	11	37881179	6.92E-52	LINC02760
26915399	12	69602666	7.01E-52	CCT2
29584780	14	54049041	7.02E-52	LOC105370507
11903873	5	33486491	7.10E-52	TARS1
19680159	8	92126409	7.22E-52	RUNX1T1
29073452	13	112697727	7.33E-52	ATP11A
21675629	9	105161311	7.56E-52	LOC112268038
24204041	11	11699558	7.58E-52	LOC107984311
37258406	21	33045533	7.68E-52	LINC00945
7020733	3	56332152	7.73E-52	ERC2
29332082	14	34655450	7.76E-52	LOC107984628
32102430	16	50389669	7.83E-52	LINC02178
17292828	7	78227850	7.84E-52	MAGI2
30027406	14	89001496	7.99E-52	LOC124903356
5267343	2	160995972	8.14E-52	TANK-AS1
1172713	1	90116283	8.16E-52	ZNF326
37847720	22	37773904	8.28E-52	TRIOBP
24376160	11	25070770	8.35E-52	LUZP2
21776128	9	113006097	8.40E-52	ZNF883
10519941	4	120326353	8.77E-52	MAD2L1
9968903	4	74187800	8.86E-52	MTHFD2L
35798239	19	48059270	8.86E-52	PLA2G4C
37105097	21	21272934	9.11E-52	NCAM2
11607227	5	10943358	9.35E-52	CTNND2
1097390	1	83667466	9.35E-52	LINC01725
1146396	1	87768696	9.93E-52	LOC124904212
559703	1	39187895	1.02E-51	MACF1
33583364	17	56386580	1.05E-51	ANKFN1
15361255	6	114425330	1.05E-51	LOC107986638

32166302	16	55427624	1.08E-51	MMP2
5026671	2	140932886	1.09E-51	LRP1B
26936619	12	71249220	1.09E-51	TSPAN8
22412087	10	17970674	1.28E-51	SLC39A12
32533646	16	81229856	1.31E-51	BCO1
3992687	2	65518069	2.31E-51	LOC124907784
31006791	15	68719050	3.16E-50	CORO2B
5357888	2	168600190	2.36E-46	CERS6
17917093	7	129479491	3.21E-46	STRIP2
34974978	18	73286270	3.60E-46	LINC02864
28021935	13	30055622	8.81E-41	LINC00365
960072	1	72444873	9.67E-40	LOC105378797
28596917	13	76266340	3.89E-38	LOC105370261
34937235	18	70657774	4.99E-38	GTSCR1
13973017	6	14752877	5.20E-38	LOC105374945
24311940	11	20618945	4.81E-37	SLC6A5
5908466	2	214723984	1.39E-36	BARD1
8120327	3	138872770	1.53E-36	LOC124906289
8120516	3	138884904	1.71E-36	LOC124906289
1978243	1	160768683	1.80E-36	LOC105371470
8120942	3	138907533	2.20E-36	LOC124906289
7921894	3	123060552	9.95E-36	PDIA5
1035840	1	79024108	1.57E-35	ADGRL4
28623934	13	78488033	2.08E-35	OBI1-AS1
3591843	2	36572017	2.46E-35	FEZ2
879532	1	65511429	2.50E-35	LEPR
5021734	2	140592935	2.97E-35	LRP1B
15096864	6	92986922	2.99E-35	LOC124901507
6990422	3	53975047	3.14E-35	LOC105377095
3282861	2	12625908	3.17E-35	MIR3681HG
5006192	2	139356053	3.20E-35	LOC105373643
23909971	10	126912727	3.21E-35	DOCK1
2040963	1	165576189	3.22E-35	LRRC52-AS1
9981356	4	75197111	3.23E-35	LOC100506253
7163389	3	66688230	3.33E-35	LOC105377142
24555376	11	39175193	3.34E-35	LOC101928563
27998610	13	28303492	3.35E-35	FLT1
32246044	16	61315301	3.40E-35	LOC105371301
22137400	10	449920	3.40E-35	DIP2C
3914976	2	59267309	3.41E-35	LINC01122
36058483	20	5908014	3.43E-35	CHGB
11708801	5	18768192	3.44E-35	LINC02100
16624176	7	35359752	3.46E-35	LOC401324
6369445	3	5220548	3.47E-35	EDEM1
2903802	1	235637653	3.47E-35	GNG4

9698081	4	54455623	3.48E-35	LINC02283
15095098	6	92861442	3.52E-35	LOC124901507
1458730	1	113449360	3.60E-35	MAGI3
31047461	15	72028218	3.60E-35	MYO9A
25183023	11	77233446	3.66E-35	GDPD4
7178595	3	67802372	3.82E-35	SUCLG2-DT
12780019	5	104198262	3.82E-35	NIHCOLE
35473487	19	23114300	3.84E-35	ZNF730
21860387	9	119680273	3.85E-35	LOC105376250
28380427	13	59621319	3.88E-35	DIAPH3
29756760	14	67501044	3.92E-35	TMEM229B
14206868	6	30382634	4.02E-35	LOC105375012
21332564	9	78336357	4.05E-35	PSAT1
14685488	6	61573792	4.06E-35	KHDRBS2
27313366	12	102691142	4.09E-35	LINC02456
18167941	7	149184790	4.12E-35	ZNF398
21802448	9	115014741	4.13E-35	DELEC1
27618297	12	126514151	4.17E-35	LINC02347
8621274	3	179501298	4.32E-35	GNB4
23512417	10	95648320	4.39E-35	ALDH18A1
10366705	4	107719187	4.42E-35	PAPSS1
6413820	3	8026242	4.43E-35	LOC101927394
5186405	2	154154934	4.47E-35	GALNT13
5767745	2	202969578	4.47E-35	CARF
14419784	6	41980010	4.47E-35	CCND3
17494104	7	94865221	4.47E-35	PPP1R9A
25075616	11	69147791	4.47E-35	LOC338694
35279874	19	10512925	4.47E-35	S1PR5
37285161	21	35110715	4.47E-35	LOC101928269
37429727	21	45123274	4.48E-35	ADARB1
5492714	2	179330791	4.50E-35	SESTD1
32657346	16	87589624	4.50E-35	LOC124903749
28814495	13	93499091	4.51E-35	GPC6
28178519	13	42639949	4.51E-35	TNFSF11
16882408	7	55089939	4.52E-35	EGFR
410138	1	27258529	4.53E-35	WDTC1
37475243	22	11367008	4.53E-35	LOC107984037
31691426	16	12266686	4.54E-35	SNX29
37501322	22	12601391	4.54E-35	LOC105379428
19848417	8	106054334	4.56E-35	ZFPM2-AS1
26140379	12	14606583	4.58E-35	PLBD1-AS1
31606339	16	7479115	4.60E-35	RBFOX1
8733933	3	188624779	4.61E-35	LPP
14102381	6	24077506	4.62E-35	NRSN1
27101585	12	84836709	4.62E-35	SLC6A15

7227718	3	71609961	4.65E-35	EIF4E3
24556916	11	39278019	4.65E-35	LOC101928563
159062	1	9755798	4.66E-35	CLSTN1
9287975	4	26735441	4.67E-35	TBC1D19
25184354	11	77343263	4.76E-35	PAK1
17230833	7	73629974	4.77E-35	MLXIPL
5210304	2	156174804	4.79E-35	LINC01876
9426995	4	37062068	4.81E-35	LOC101928721
7666268	3	102259758	4.84E-35	ZPLD1
20036770	8	121891348	4.85E-35	HAS2-AS1
3421946	2	23661340	4.87E-35	KLHL29
8756213	3	190328118	4.91E-35	CLDN16
23967584	10	130753068	4.91E-35	LOC105378562
20741039	9	24647425	4.95E-35	LOC105375994
14506101	6	48730741	4.96E-35	LOC107986602
18984115	8	37108093	4.96E-35	LOC105379377
7090118	3	61451735	4.97E-35	LOC105377114
16152819	7	2783649	4.98E-35	GNA12
765937	1	56249737	5.00E-35	PLPP3
21256125	9	72066764	5.01E-35	C9orf57
1501724	1	117089260	5.05E-35	TTF2
35154653	19	3180465	5.06E-35	S1PR4
1206015	1	92881326	5.06E-35	DIPK1A
17518112	7	97023023	5.06E-35	DLX5
6727956	3	31687893	5.09E-35	OSBPL10
25936314	12	456435	5.10E-35	B4GALNT3
27221167	12	95239948	5.12E-35	VEZT
30834064	15	55836331	5.12E-35	NEDD4
6940116	3	49464586	5.13E-35	DAG1
827640	1	61319790	5.17E-35	NFIA
25601627	11	109834328	5.20E-35	LOC105369483
15374685	6	115386259	5.21E-35	LOC105377961
25627733	11	112118289	5.24E-35	BCO2
32113860	16	51327863	5.24E-35	LOC107984886
16364143	7	15845901	5.25E-35	LOC105375167
5717822	2	198480202	5.26E-35	LOC105373830
14359047	6	37340029	5.26E-35	TBC1D22B
21890012	9	122274533	5.27E-35	MRRF
26265324	12	24505874	5.27E-35	SOX5
35725852	19	43082880	5.29E-35	PSG2
23516921	10	96040787	5.32E-35	ENTPD1-AS1
10567425	4	124349235	5.36E-35	LOC105377407
7878149	3	119528923	5.38E-35	CD80
24260474	11	16480645	5.42E-35	SOX6
9508781	4	43165325	5.42E-35	GRXCR1

2107042	1	170942368	5.42E-35	MROH9
31290450	15	90522848	5.44E-35	CRTC3
18855780	8	26626482	5.46E-35	DPYSL2
8035914	3	131799919	5.47E-35	CPNE4
29751491	14	67108712	5.56E-35	LOC105370538
24136716	11	6722217	5.59E-35	GVINP1
21707508	9	107754561	5.62E-35	LOC105376208
6946818	3	50066831	5.63E-35	RBM6
30589211	15	36877309	5.64E-35	LOC145845
16452845	7	22013723	5.67E-35	CDCA7L
26256685	12	23839828	5.68E-35	SOX5
21565643	9	96422422	5.77E-35	ZNF367
36781695	20	57539225	5.81E-35	LOC124904941
31633321	16	8772252	5.82E-35	ABAT
20036714	8	121885772	5.84E-35	HAS2-AS1
2959154	1	239602090	5.86E-35	CHRM3
20400172	9	2685997	5.87E-35	LOC105375957
4122443	2	75877688	5.89E-35	LOC105374813
23568904	10	100297702	5.94E-35	PKD2L1
23809910	10	119724900	5.96E-35	INPP5F
10073328	4	82803162	5.98E-35	SCD5
30588954	15	36851886	6.04E-35	LOC124903595
14083661	6	22921428	6.07E-35	LOC105374974
31101918	15	76673952	6.17E-35	SCAPER
23516568	10	96011752	6.19E-35	ENTPD1-AS1
25993001	12	4336446	6.28E-35	TIGAR
18942827	8	33469803	6.32E-35	FUT10
24304369	11	20085195	6.39E-35	NAV2
23669456	10	108526266	6.40E-35	LOC105378477
29641740	14	58386173	6.46E-35	TOMM20L-DT
5760588	2	202338600	6.49E-35	NOP58
19990945	8	118256779	6.61E-35	SAMD12
10472371	4	116509621	6.96E-35	LOC105377384
23321861	10	80441487	6.98E-35	PRXL2A
18019949	7	137642929	6.98E-35	DGKI
16237003	7	7309901	7.11E-35	LINC03016
12693422	5	97377441	7.26E-35	LIX1-AS1
535808	1	37303973	7.35E-35	LOC107984942
17311692	7	79592857	7.44E-35	LOC105375370
10203330	4	93535231	7.46E-35	GRID2
20789679	9	28088203	7.68E-35	LINGO2
35234690	19	7777552	7.70E-35	CLEC4M
5344132	2	167491483	7.97E-35	B3GALT1
5407723	2	172323908	8.08E-35	ITGA6
35016507	18	76256577	8.53E-35	LOC105372209

27950372	13	24764888	9.86E-35	RNF17
27214656	12	94776001	1.33E-34	KRT19P2
14994088	6	84911255	2.15E-34	LOC105377881
3310703	2	14744628	2.54E-34	LRATD1
8015834	3	130127837	2.71E-34	LOC124909433
32547046	16	81930747	2.74E-34	PLCG2
20349202	8	144913678	1.16E-33	ZNF250
28774536	13	90331565	2.48E-33	LOC105370310
17192846	7	70833214	4.20E-33	AUTS2
30540239	15	33152788	6.99E-33	FMN1
13094366	5	127960800	1.02E-29	SLC12A2-DT
18754021	8	19404624	2.84E-28	CSGALNACT1
25642762	11	113381813	3.08E-28	TTC12
22079592	9	135537306	3.24E-28	OBP2A
24339964	11	22652993	5.01E-28	GAS2
1273995	1	98582736	7.59E-28	SNX7
318950	1	20291165	8.31E-28	VWA5B1
23411082	10	87324742	1.51E-26	NUTM2A-AS1
19411888	8	69762118	1.90E-26	SLCO5A1
27107892	12	85464656	3.99E-26	LINC02820
28638684	13	79721355	4.38E-26	LOC124903226
27608743	12	125785709	4.39E-26	LOC124903047
18515398	8	6902909	4.44E-26	LOC101928095
33781511	17	72493583	6.00E-26	LINC00511
37793058	22	33940795	7.72E-26	LARGE1-AS1
5223946	2	157445477	2.25E-25	CYTIP
35187883	19	5051829	5.20E-25	KDM4B
5720070	2	198675171	8.62E-25	LOC105373831
15250741	6	105457378	9.30E-25	LOC105377921
3869903	2	55930946	3.25E-23	EFEMP1
31371545	15	96235686	1.70E-22	NR2F2-AS1
25656439	11	114470582	1.84E-22	REXO2
36977408	21	10717070	2.00E-22	TPTE
1814659	1	147496409	2.25E-22	LINC00624
27417988	12	111394443	2.39E-22	LINC02356
32178154	16	56286695	5.27E-22	GNAO1
33853642	17	77343003	8.17E-22	LOC124904062
36958260	21	9863782	9.11E-22	LINC01667
25233633	11	81373154	9.22E-22	MIR4300HG
2502490	1	203602837	9.39E-22	ATP2B4
33596165	17	57460128	9.45E-22	MSI2
28196292	13	44055228	9.60E-22	NRAD1
7234099	3	72083029	9.69E-22	LINC00877
25501941	11	101734380	9.94E-22	TRPC6
25498043	11	101484595	1.01E-21	TRPC6

443369	1	30011616	1.03E-21	LINC01648
5195852	2	154932653	1.04E-21	LOC105373696
24466359	11	32269142	1.05E-21	WT1
4764931	2	120945957	1.10E-21	GLI2
36487486	20	34589104	1.12E-21	PIGU
23034168	10	57741950	1.13E-21	LOC105378314
2785718	1	226639054	1.16E-21	ITPKB
33923001	17	81422014	1.20E-21	LOC124904083
6258098	2	240710936	1.20E-21	KIF1A
5979626	2	220441872	1.21E-21	LOC105373895
16444445	7	21509471	1.21E-21	SP4
11570260	5	8237875	1.24E-21	LINC02226
24690593	11	49485495	1.24E-21	FOLH1
20420582	9	4068834	1.27E-21	GLIS3
8963932	4	4891540	1.28E-21	MSX1
23457115	10	91128047	1.29E-21	LOC124902479
30608540	15	38404731	1.29E-21	SPRED1
34058511	18	7390510	1.30E-21	LOC112577592
2142824	1	174142030	1.31E-21	RABGAP1L-DT
22254710	10	7317641	1.31E-21	SFMBT2
2202936	1	179348760	1.32E-21	SOAT1
22074721	9	135250683	1.33E-21	LOC107987138
21592756	9	98641129	1.33E-21	GABBR2
31000943	15	68270259	1.36E-21	FEM1B
25493205	11	101076919	1.37E-21	PGR
5605360	2	188777932	1.37E-21	LOC124906107
23330743	10	81137477	1.39E-21	LINC02655
3494264	2	29636337	1.39E-21	ALK
8830873	3	195599129	1.39E-21	APOD
12227159	5	57058713	1.39E-21	MIER3
21941446	9	126470646	1.39E-21	MVB12B
30866894	15	58243452	1.39E-21	ALDH1A2
35904832	19	54482846	1.39E-21	LOC105372460
36620989	20	45640436	1.39E-21	WFDC11
20728451	9	23679191	1.39E-21	LOC101929563
2427358	1	197444906	1.39E-21	CRB1
6288673	3	242367	1.39E-21	CHL1
10077996	4	83178478	1.39E-21	PLAC8
26770157	12	57486889	1.40E-21	ARHGAP9
3765604	2	48590636	1.40E-21	STON1-GTF2A1L
16820108	7	50637718	1.40E-21	GRB10
24214297	11	12504774	1.40E-21	PARVA
19234248	8	55127240	1.41E-21	XKR4
24080931	11	3244495	1.41E-21	MRGPRE
19682520	8	92316765	1.41E-21	LOC105375638

31557756	16	5447378	1.41E-21	RBFOX1
11693342	5	17665349	1.42E-21	H3Y1
22981284	10	53871451	1.42E-21	PCDH15
966781	1	73050541	1.42E-21	LOC105378798
3368202	2	19218734	1.43E-21	LINC01376
2523425	1	205220945	1.43E-21	TMCC2
10075732	4	83000827	1.43E-21	LIN54
4971582	2	136592921	1.44E-21	LOC105373633
1137686	1	87018132	1.44E-21	HS2ST1
5610822	2	189285071	1.45E-21	COL5A2
8743628	3	189429455	1.45E-21	TPRG1
11749612	5	21891572	1.45E-21	CDH12
9359908	4	32155263	1.45E-21	LOC102723846
10810494	4	143725181	1.45E-21	LOC105377459
8830975	3	195603220	1.48E-21	APOD
6434828	3	9556778	1.48E-21	LHFPL4
17639878	7	105771201	1.49E-21	ATXN7L1
3983178	2	64776484	1.49E-21	LOC105374777
9788929	4	60841120	1.49E-21	LINC02496
7457396	3	89046422	1.49E-21	EPHA3
19547273	8	80959248	1.50E-21	PAG1
28461536	13	65728154	1.50E-21	LOC105370241
11091980	4	166106724	1.50E-21	TLL1
24543953	11	38312922	1.51E-21	LOC105376634
8830685	3	195586554	1.51E-21	APOD
24487381	11	33970684	1.52E-21	LOC105376621
3521086	2	31714419	1.53E-21	SRD5A2
29193852	14	23666280	1.54E-21	DHRS2
26720141	12	53334171	1.54E-21	ESPL1
29571139	14	52926411	1.54E-21	FERMT2
18982641	8	36989571	1.54E-21	LOC105379376
9681103	4	53143795	1.54E-21	SCFD2
24934578	11	58106159	1.55E-21	OR9Q1
37911602	22	42639843	1.55E-21	ATP5MGL
6311247	3	1608042	1.55E-21	CNTN6
14812392	6	70902617	1.56E-21	B3GAT2
15057693	6	89887381	1.57E-21	BACH2
11772757	5	23695576	1.58E-21	LOC124901158
23387776	10	85474010	1.58E-21	LINC01520
18859737	8	26912460	1.59E-21	ADRA1A
11281381	4	180681751	1.59E-21	LOC124900820
18093962	7	143430851	1.59E-21	LOC105375547
6489089	3	13519826	1.60E-21	HDAC11
19422653	8	70687312	1.60E-21	XKR9
16011908	6	166231283	1.60E-21	LOC107986668

32944404	17	14736124	1.60E-21	LOC105371545
37696474	22	26665247	1.60E-21	MIAT
3371000	2	19451288	1.61E-21	LOC105373458
964712	1	72874417	1.61E-21	LOC105378798
106151	1	6031724	1.63E-21	KCNAB2
16197035	7	5207768	1.63E-21	WIPI2
5800216	2	205787718	1.63E-21	NRP2
32440865	16	76451599	1.64E-21	CNTNAP4
618245	1	44018597	1.65E-21	SLC6A9
2776349	1	225878885	1.65E-21	TMEM63A
11052736	4	163267837	1.65E-21	NPY1R
8277331	3	151864952	1.67E-21	SUCNR1
18469506	8	5058772	1.67E-21	CSMD1
29850341	14	75055079	1.67E-21	ACYP1
37092483	21	20441659	1.67E-21	LINC02573
32087829	16	49227471	1.68E-21	LOC105371241
29456800	14	44191188	1.68E-21	LINC02307
19192080	8	51743908	1.69E-21	PXDNL
28108711	13	37149185	1.69E-21	CSNK1A1L
6115958	2	231237175	1.70E-21	ARMC9
7110049	3	62908949	1.70E-21	CADPS
479317	1	32687855	1.70E-21	SYNC
21961826	9	128083513	1.71E-21	SLC25A25
24655649	11	47142241	1.72E-21	CSTPP1
2425654	1	197290265	1.73E-21	CRB1
23734167	10	113621314	1.74E-21	NRAP
30356100	15	20350666	1.75E-21	LOC124903442
35395271	19	18045884	1.79E-21	IL12RB1
24102333	11	4376137	1.79E-21	OR52B4
2508559	1	204032071	1.82E-21	LOC105371689
11826954	5	27832327	1.82E-21	LOC124901177
12569194	5	85924787	1.83E-21	LOC105379061
6667928	3	27008026	1.83E-21	NEK10
22464618	10	21415486	1.85E-21	LOC124902390
16213157	7	6013255	1.88E-21	AIMP2
30900048	15	60547625	1.89E-21	RORA
23544178	10	98287110	1.90E-21	LOXL4
14417619	6	41799079	1.98E-21	USP49
1388844	1	107624374	2.02E-21	VAV3
23129806	10	65607570	2.24E-21	LINC01515
28343552	13	56470983	2.27E-21	LOC105370214
8988570	4	6408873	6.11E-21	PPP2R2C
25810008	11	126422296	6.57E-21	ST3GAL4
6001179	2	222178834	1.16E-20	PAX3
8257198	3	150221750	1.74E-20	LOC105374313

27900373	13	21491515	1.95E-20	MIPEPP3
606405	1	43012615	2.11E-20	SLC2A1-DT
37188187	21	27503970	2.19E-20	LOC105372762
10781147	4	141218022	2.76E-20	ZNF330
5409187	2	172435027	2.88E-20	ITGA6
9874123	4	66968066	2.98E-20	LOC105377262
6834152	3	40371794	6.66E-20	ENTPD3-AS1
34695362	18	52439174	7.32E-20	DCC
34818193	18	61727968	7.84E-20	LOC124904314
33653783	17	62459779	2.49E-19	TLK2
19151384	8	48642452	5.90E-19	LOC101929268
19232334	8	54972005	7.20E-19	LOC107986887
981924	1	74332976	8.13E-19	TNNI3K
4959992	2	135683570	1.48E-18	R3HDM1
25061780	11	68191173	3.00E-18	KMT5B
11506685	5	3667030	3.16E-18	IRX1
32114505	16	51378394	3.29E-18	LOC105371256
26735356	12	54681258	3.56E-18	DCD
939890	1	70624068	3.67E-18	LINC01788
597354	1	42237911	4.27E-18	FOXJ3
29354906	14	36382258	4.97E-18	MBIP
23638803	10	106197432	1.39E-17	LINC02624
20434340	9	4806017	1.97E-17	RCL1
19123570	8	46338774	2.22E-17	ASNSP1
32389915	16	73065566	2.30E-17	ZFHX3
25472092	11	99591337	3.11E-17	CNTN5
5863897	2	211130709	3.47E-17	ERBB4
21443122	9	87146773	3.58E-17	LOC105376126
23811528	10	119854540	3.94E-17	MCMBP
4557631	2	104881121	1.01E-16	LINC01159
1420171	1	110307491	1.28E-16	RBM15-AS1
35853817	19	51663137	1.41E-16	SIGLEC14
30050382	14	90749402	1.42E-16	TTC7B
14202336	6	30094812	1.44E-16	LOC124901298
31490198	16	1421168	1.81E-16	UQCC4
13416656	5	154973071	2.34E-16	MRPL22
15185425	6	100342829	3.43E-16	SIM1
15164407	6	98607959	3.46E-16	LOC101927314
27855497	13	18584387	3.73E-16	LINC00349
18177582	7	149907478	4.29E-16	ACTR3C
31884261	16	26344052	4.45E-16	LOC102723536
36170714	20	15051338	4.53E-16	MACROD2
409787	1	27227580	4.61E-16	WDTC1-DT
13409504	5	154416480	4.68E-16	GALNT10
14744234	6	65921174	4.68E-16	LOC105377841

6708772	3	30199926	5.38E-16	LOC124906224
6708799	3	30201231	5.38E-16	LOC124906224
7925592	3	123359331	5.84E-16	ADCY5
29375336	14	38122073	6.03E-16	LOC105370456
9233663	4	22575380	8.62E-16	ADGRA3
23333439	10	81350158	9.38E-16	LOC124902549
2712999	1	220883284	1.02E-15	MTARC1
1855741	1	150811458	1.04E-15	ARNT
36979780	21	10773049	1.09E-15	TPTE
30018960	14	88287674	1.10E-15	KCNK10
34939196	18	70812834	1.64E-15	LOC105372185
26249290	12	23241346	2.41E-15	LINC02955
34993549	18	74570033	2.67E-15	CNDP1
28339352	13	56073873	3.50E-15	LOC105370214
34720147	18	54046507	4.85E-15	MBD2
15436809	6	120501139	5.43E-15	MIR3144
15116972	6	94568546	5.56E-15	LOC105377901
25610244	11	110548524	5.79E-15	ARHGAP20
30996705	15	67959191	5.89E-15	SKOR1
3544493	2	33467980	6.25E-15	RASGRP3
31101918	15	76673952	7.46E-15	SCAPER
8022603	3	130616552	8.52E-15	COL6A6
1388844	1	107624374	9.13E-15	VAV3
28299235	13	52701802	1.17E-14	SUGT1
23210772	10	71584542	1.22E-14	CDH23
2201489	1	179245952	1.51E-14	ABL2
34996174	18	74767820	1.72E-14	ZNF407
18047991	7	139809233	2.30E-14	TBXAS1
36225127	20	19032488	3.40E-14	LOC107985442
30998232	15	68068893	6.06E-14	PIAS1
17897444	7	127908318	6.27E-14	SND1
5227554	2	157799371	6.36E-14	ACVR1
20577091	9	13565159	6.49E-14	LOC124902326
29621845	14	56826238	6.58E-14	OTX2-AS1
6250077	2	240242161	6.75E-14	LOC105373968
19432610	8	71550953	7.20E-14	EYA1
2989156	1	241831871	8.26E-14	LOC124904603
20095704	8	126819449	1.14E-13	LOC105375753
16434408	7	20802489	1.27E-13	SP8
7244676	3	72954399	1.45E-13	GXYLT2
3208698	2	7174967	1.73E-13	LOC101929452
33697291	17	66067016	1.88E-13	CEP112
7923926	3	123222125	1.91E-13	SEC22A
27904148	13	21798755	2.09E-13	LOC124903132
35424025	19	20108731	2.40E-13	ZNF90

17331552	7	81133888	2.64E-13	SEMA3C
8830596	3	195580368	3.56E-13	APOD
22254145	10	7271291	4.49E-13	SFMBT2
2776349	1	225878885	4.93E-13	TMEM63A
14777165	6	68143362	5.66E-13	LOC105377846
5952385	2	218197902	5.88E-13	LOC101928487
27638703	12	127948345	6.68E-13	LINC00508
13219392	5	138830166	7.94E-13	CTNNA1
3769387	2	48862534	1.03E-12	STON1-GTF2A1L
2629262	1	214031034	1.10E-12	PROX1
30579343	15	36084344	1.31E-12	LOC102724214
32340130	16	69224210	1.31E-12	SNTB2
36000210	20	1745046	1.44E-12	LOC124904858
26262983	12	24326791	2.18E-12	SOX5
31158147	15	80839289	2.20E-12	CEMIP
26109129	12	12249671	2.85E-12	ETV6
13273354	5	143515310	3.53E-12	LOC105378208
10978689	4	157621987	3.54E-12	LINC02433
19940578	8	113896592	3.56E-12	LOC105375710
5278102	2	162009513	4.07E-12	DPP4
26151121	12	15507450	6.41E-12	PTPRO
33754257	17	70400523	6.68E-12	LOC124904100
4147423	2	77509231	6.99E-12	LRRTM4
19525347	8	78960238	1.26E-11	LOC105375912
15517958	6	127309480	1.32E-11	ECHDC1
1855741	1	150811458	1.59E-11	ARNT
27632516	12	127525427	1.76E-11	LOC105370067
30549385	15	33809612	1.76E-11	RYR3
11875583	5	31325321	1.86E-11	CDH6
18567570	8	9470581	1.91E-11	LOC124901883
9494304	4	42058100	1.97E-11	SLC30A9
1348754	1	104577157	2.15E-11	THAP3P1
418564	1	28020667	2.17E-11	EYA3
37343113	21	39550915	2.18E-11	B3GALT5-AS1
28884944	13	99108622	2.39E-11	DOCK9-DT
9940361	4	71867311	2.52E-11	GC
10829478	4	145215419	2.55E-11	LOC105377465
25386902	11	93054036	2.62E-11	MTNR1B
7665050	3	102134467	2.68E-11	ZPLD1
1380014	1	106827352	2.89E-11	LINC01661
14726104	6	64603861	2.95E-11	EYS
14513542	6	49307993	3.13E-11	MMUT
8691031	3	185368959	3.18E-11	MAP3K13
12645282	5	92932571	3.34E-11	LINC02058
8862490	3	197239843	3.49E-11	DLG1

15643101	6	137790265	3.50E-11	LINC02539
19332906	8	63014893	4.17E-11	GGH
8844013	3	196070774	4.23E-11	TFRC
12016264	5	42474295	4.40E-11	GHR
34937614	18	70689508	4.41E-11	LOC124904322
2065301	1	167540386	4.51E-11	LOC107985224
30059694	14	91472824	4.54E-11	PPP4R3A
11749925	5	21915822	4.60E-11	CDH12
6933292	3	48894183	4.64E-11	SLC25A20
19949069	8	114555511	4.70E-11	LOC105375710
10932760	4	153927965	4.94E-11	LOC101927947
3074379	1	247601147	4.97E-11	LOC102724446
13253821	5	141996416	5.02E-11	GNPDA1
10464000	4	115825116	5.07E-11	LOC105377383
18914895	8	31125971	5.38E-11	WRN
15500270	6	125485362	5.55E-11	LOC102723341
28367348	13	58582543	5.66E-11	LOC105370218
14182845	6	29657663	6.03E-11	OR2H2
32477556	16	78501193	6.30E-11	WWOX
22042490	9	133498358	6.84E-11	LOC102723855
37409471	21	43931922	6.99E-11	AGPAT3
3726662	2	45862183	7.58E-11	PRKCE
18774926	8	20782458	8.87E-11	LOC105379315
6863100	3	42673787	1.68E-10	LOC124906232
33305496	17	33840799	1.79E-10	ASIC2
21495333	9	90924126	1.83E-10	LOC105379829
23098093	10	63054653	1.88E-10	LOC105378328
33324644	17	35357139	1.90E-10	SLFN11
1114291	1	85110450	1.90E-10	DNAI3
15994557	6	164983378	1.90E-10	MEAT6
20716176	9	22824894	1.93E-10	LOC107987054
5784615	2	204475338	1.93E-10	LOC105373845
29561370	14	52157281	1.93E-10	LINC02319
30004053	14	87140915	1.96E-10	LOC105370608
27457099	12	114635497	2.06E-10	LOC124903026
11695550	5	17826069	2.13E-10	LINC02223
32201546	16	57961631	2.21E-10	CNGB1
37258406	21	33045533	2.37E-10	LINC00945
32950498	17	15156064	2.42E-10	CDRT8
27871362	13	19389309	2.50E-10	TPTE2
6527053	3	16445483	2.51E-10	RFTN1
9060311	4	10020039	2.52E-10	SLC2A9
23764811	10	116205596	2.57E-10	GFRA1
11581451	5	9035959	2.61E-10	SEMA5A
21419931	9	85304300	2.62E-10	LOC124902322

29758394	14	67627256	2.69E-10	ARG2
13576368	5	167815972	2.71E-10	TENM2
35113328	19	1025180	2.72E-10	CNN2
16804345	7	49400543	2.73E-10	LOC124901804
16408186	7	19108729	2.77E-10	TWIST1
26140379	12	14606583	2.79E-10	PLBD1-AS1
7613120	3	98008798	2.92E-10	GABRR3
24563699	11	39763834	3.00E-10	LOC107984362
32758230	17	2121587	3.04E-10	SMG6
11281381	4	180681751	3.12E-10	LOC124900820
10958665	4	156059405	3.12E-10	LOC102724785
9249584	4	23859780	3.16E-10	PPARGC1A
24694943	11	49802544	3.20E-10	OR4C13
10620974	4	128735123	3.45E-10	JADE1
28496734	13	68571149	3.45E-10	LINC00550
20875814	9	34355537	3.51E-10	MYORG
2814717	1	228791447	3.51E-10	LOC124904539
17690657	7	109942686	3.57E-10	EIF3IP1
13218306	5	138748904	3.64E-10	CTNNA1
12210918	5	55733120	3.68E-10	SLC38A9
11392937	4	188044085	3.77E-10	ZFP42
27075920	12	82871681	3.87E-10	TMTC2
2550879	1	207648174	4.22E-10	CR1L
5640531	2	191868029	5.64E-10	CAVIN2-AS1
2155868	1	175225095	6.15E-10	KIAA0040
30064851	14	91871221	6.17E-10	FBLN5
890253	1	66414958	6.74E-10	LOC105378776
3501580	2	30227016	7.21E-10	LBH
26565112	12	40926171	7.31E-10	CNTN1
34965498	18	72660545	7.47E-10	CBLN2
5154938	2	151698064	7.55E-10	NEB
6955081	3	50847882	9.22E-10	DOCK3
20502516	9	8970917	9.82E-10	PTPRD
7170511	3	67195997	9.88E-10	MIR4272
36644745	20	47456863	1.00E-09	LINC01754
23696202	10	110595917	1.08E-09	SMC3
27877811	13	19878517	1.12E-09	ZMYM5
409928	1	27238998	1.16E-09	WDTC1
28493273	13	68297134	1.17E-09	LOC101927072
17475966	7	93172862	1.31E-09	HEPACAM2
12166513	5	52238528	1.32E-09	LOC105378961
7788483	3	112085348	1.34E-09	C3orf52
15128708	6	95537760	1.37E-09	MANEA-DT
24131057	11	6247043	1.46E-09	CNGA4
29646708	14	58817569	1.47E-09	LINC01500

9678148	4	52860621	1.57E-09	RASL11B
18910457	8	30766010	1.64E-09	UBXN8
23639867	10	106262590	1.67E-09	LOC102724439
29671471	14	60903135	1.69E-09	MNAT1
37981178	22	47001299	1.74E-09	TBC1D22A
25990604	12	4155618	1.92E-09	LOC105369612
8340787	3	157172182	1.96E-09	LOC101928236
34609333	18	45818732	2.01E-09	EPG5
5703907	2	197216869	2.04E-09	ANKRD44
16675576	7	39312189	2.06E-09	POU6F2
27007752	12	77149448	2.48E-09	LOC105369853
34954625	18	71863690	2.63E-09	LOC105376873
18920231	8	31559630	2.83E-09	LOC105379360
14387558	6	39487990	2.99E-09	KIF6
20357376	9	277457	3.10E-09	DOCK8
3264818	2	11282607	3.19E-09	ROCK2
24577915	11	40880863	3.52E-09	LRRC4C
28556216	13	73049804	3.86E-09	KLF5
10525245	4	120734391	4.44E-09	PRDM5
22353260	10	13955112	4.78E-09	FRMD4A
12710034	5	98687416	5.14E-09	RGMB
27871070	13	19376911	5.25E-09	LINC00421
34979345	18	73557833	5.26E-09	LOC105372190
1510936	1	117847663	5.30E-09	GDAP2
8688104	3	185113285	5.31E-09	C3orf70
7127524	3	64163931	5.36E-09	PRICKLE2
17445720	7	90459233	5.86E-09	CLDN12
13299132	5	145551400	5.96E-09	PRELID2
20794040	9	28375089	6.25E-09	LINGO2
32464971	16	77962739	6.31E-09	VAT1L
16462622	7	22774537	6.37E-09	TOMM7
34675067	18	50862392	6.40E-09	ME2
10397275	4	110233564	6.46E-09	LOC124900759
24383317	11	25513851	7.03E-09	LINC02699
17508826	7	96192684	7.06E-09	SLC25A13
5770830	2	203258388	7.15E-09	CYP20A1
19234452	8	55144805	7.31E-09	XKR4
30627248	15	39872911	7.93E-09	GPR176
36841084	20	61826962	8.41E-09	CDH4-AS2
35908392	19	54656999	8.51E-09	MIR8061
27216479	12	94909523	8.58E-09	LOC105369915
37882802	22	40489971	9.57E-09	MRTFA
3536248	2	32918608	1.03E-08	LINC00486
14034970	6	19313406	1.09E-08	LOC105374961
33580507	17	56133528	1.10E-08	ANKFN1

11307745	4	182440114	1.38E-08	TENM3
19434919	8	71729055	1.38E-08	LOC107986890
29026123	13	109680197	1.45E-08	LOC107984602
32706297	16	89806347	1.45E-08	FANCA
2428354	1	197536160	1.56E-08	DENND1B
1512084	1	117951508	1.66E-08	WDR3
9962495	4	73636766	1.80E-08	RASSF6
5178921	2	153587232	2.07E-08	GALNT13
3662670	2	41508739	2.10E-08	LOC105374506
815941	1	60357864	2.37E-08	LOC105378761
17670055	7	108226530	2.38E-08	NRCAM
27375069	12	107787555	2.41E-08	ASCL4
26276984	12	25385501	2.50E-08	LMNTD1
5859770	2	210796392	2.56E-08	CPS1
19575870	8	83300340	2.59E-08	LINC01419
9941332	4	71927210	2.63E-08	NPFFR2
2423375	1	197073313	2.67E-08	ASPM
22346165	10	13524644	2.68E-08	BEND7
13907071	6	9710897	2.68E-08	OFCC1
29548697	14	51196767	2.74E-08	TMX1
30883454	15	59384417	2.74E-08	FAM81A
25590956	11	108948870	2.88E-08	DDX10
7342771	3	79852375	2.94E-08	ROBO1
25807885	11	126261155	3.10E-08	FAM118B
4720684	2	117509895	3.23E-08	LOC124907878
37738168	22	29896869	3.23E-08	MTMR3
30797835	15	53415250	3.29E-08	WDR72
36253547	20	21290959	3.65E-08	XRN2
8688247	3	185125384	3.80E-08	C3orf70
7855581	3	117736184	4.08E-08	LSAMP
9525582	4	44460650	4.12E-08	KCTD8
17669721	7	108195620	4.23E-08	NRCAM
