# Supplementary material for: Integrating Polygenic Risk and Ocular Phenotyping Reveals an Axial-Length–Dominant Mechanism in High and Extreme High Myopia
Source: Ophthalmol Sci. 2026 Apr 15;6(6):101194. doi: 10.1016/j.xops.2026.101194 (PMC13195619; doi:10.1016/j.xops.2026.101194)
Supplement: Table S3 [file mmc3.pdf]

Table S3. Analysis-ready dataset for myopia severity and PRS analyses

SampleID	Age	Sex	PRS_Score	AxialLength_OD_mm	AxialLength_OS_mm
24BY04532	8	Female	13.73529412	23.47	22.97
24BY04534	11	Female	15.45833333	25.79	25.56
24BY04536	13	Female	10.67346939	25.19	25.17
24BY04539	10	Female	11.95121951	23.52	23.62
24BY04540	13	Female	11.15217391	26.74	27.11
24BY04542	13	Female	26.8	24.93	23.96
24BY04627	8	Male	12.15	25.54	24.98
24BY04629	8	Female	12.89473684	24.88	24.97
24BY04636	13	Male	15.96	26.79	26.71
24BY04640	4	Male	13.875	22.07	22.07
24BY05001	13	Male	26	27.27	27.15
24BY05012	14	Female	14.09677419	26.91	26.68
24BY05016	23	Female	13.85714286	26.92	26.63
24BY05247	8	Female	15.96	24.73	24.76
24BY06022	7	Male	1.004518072	22.65	22.81
24BY06024	11	Male	8.578125	26	25.89
24BY06097	5	Male	26.8	22.12	22.18
24BY06099	5	Male	15.82608696	23.03	22.94
24BY06166	6	Female	8.378787879	23.52	23.47
24BY06168	8	Male	7.929577465	24.51	24.49
24BY06171	14	Female	11.92857143	NA	NA
24BY06172	9	Male	13.60606061	24.06	23.57
24BY06175	11	Female	15.96	25.96	26.01
24BY06177	7	Male	12.38461538	24.17	23.72
24BY06180	8	Male	10.77083333	26.34	26.34
24BY06182	8	Female	14.26666667	23.48	23.48
24BY06184	8	Male	15.45833333	24.31	24.45
24BY06186	13	Female	13.73529412	23.99	23.77
24BY06191	7	Male	13.60606061	22.52	21.39
24BY06201	7	Male	15.96	23.26	23.32
24BY06203	6	Female	9.814814815	23.39	23.51
24BY06207	13	Male	13.875	25.55	25.73
24BY06209	8	Female	15.82608696	23.53	23.34
24BY06211	8	Male	9.24137931	24.01	24.02
24BY06213	10	Female	14.26666667	23.02	22.35
24BY06215	6	Female	11.15217391	23.12	23.23
24BY06219	14	Male	14.04545455	25.62	25.76
24BY06222	10	Female	9.814814815	25.65	25.11
24BY06223	6	Male	10.48	22.87	22.78
24BY06226	4	Female	15.07407407	21.88	21.85
24BY06229	3	Male	24	22.55	22.7
24BY06230	8	Male	13.85	23.74	24.28
24BY06234	6	Female	12.38461538	23.14	23.07

24BY06237	14	Male	7.929577465	27.11	27.18
24BY06240	9	Male	10.48	25.2	25.22
24BY06244	10	Male	24	25.63	25.62
24BY06247	7	Female	23.66666667	23.32	23.5
24BY06324	8	Female	11.31111111	23.23	23.36
24BY06342	15	Male	27.1125	27.17	26.93
24BY06348	6	Female	15.45833333	22.97	22.96
24BY06349	6	Female	32	24.83	25.36
24BY06350	17	Female	12.15	25.19	25.97
24BY06351	12	Male	14.26666667	26.38	26.35
24BY06352	9	Male	10.77083333	24.37	24.27
24BY06353	12	Female	15.82608696	23.95	23.81
24BY06354	11	Female	13.36111111	23.56	23.13
24BY06355	8	Female	16.3	24.29	24.09
24BY06356	11	Female	7.1125	24.66	24.72
24BY06357	12	Male	11.79069767	24.62	24.67
24BY06358	1	Male	9.083333333	26.16	27.23
24BY06359	9	Female	14.04545455	24.21	24.21
24BY06360	6	Male	11.92857143	24.81	24.66
24BY06361	10	Female	1.016793893	23.58	23.51
24BY06362	12	Male	11.15217391	23.56	23.43
24BY06363	10	Female	11.95121951	24.72	25.06
24BY06366	10	Female	15.96	23.23	23.14
24BY06374	6	Female	13.73529412	22.93	22.82
24BY06375	13	Female	26.8	25.64	25.58
24BY06376	8	Male	1.464530892	24.91	25.14
24BY06377	7	Male	9.814814815	23.64	23.83
24BY06378	8	Female	15.82608696	25.08	25.33
24BY06379	9	Male	14.04545455	23.53	23.4
24BY06398	11	Female	13.60606061	25.61	25.81
24BY06399	11	Male	8.378787879	26.43	26.38
24BY06468	10	Male	15.45833333	25.05	25.1
24BY06471	11	Female	13.60606061	26.04	30.67
24BY06473	11	Male	13.875	26.45	26.17
24BY06475	12	Male	10.67346939	25.04	24.61
24BY06477	7	Female	24	24.96	25
24BY06479	8	Female	9.083333333	23.44	23.36
24BY06481	8	Female	15.96	25.21	25.32
24BY06485	8	Male	11.15217391	24.12	24.38
24BY06488	13	Female	15.96	25.92	26.22
24BY06713	10	Male	15.45833333	26.08	25.93
24BY06715	11	Male	11.95121951	24.82	24.72
24BY06717	12	Female	9.814814815	25.85	25.96
24BY06719	13	Male	11.15217391	26.08	25.8
24BY06721	9	Female	14.09677419	23.42	23.39

24BY06723	7	Female	7.1125	24.58	24.54
24BY06726	8	Female	15.96	23.2	23.07
24BY06727	6	Male	12.89473684	24.25	24.39
24BY06730	11	Male	9.814814815	27.7	26.29
24BY06733	1	Male	13.875	27.89	27.61
24BY06736	9	Male	8.983606557	22.87	22.91
24BY06739	6	Female	13.875	22.35	22.26
24BY06741	8	Male	1.01993865	24.72	25.17
24BY06743	15	Female	11.15217391	25.13	25.12
24BY06746	12	Male	8.983606557	28.39	27.96
24BY06748	6	Male	10.67346939	23.24	23.33
24BY06750	12	Female	9.403508772	24.1	24.32
24BY06752	10	Female	15.14285714	23.91	24.21
24BY06754	10	Male	15.82608696	26.15	25.95
24BY06758	7	Female	13.60606061	22.55	22.48
24BY06760	5	Male	1.204797048	23.08	23.15
24BY06763	9	Male	10.9787234	25.75	25.45
24BY06768	13	Female	15.61538462	25.53	24.53
24BY06769	7	Female	14.04545455	23.24	22.77
24BY06772	9	Female	13.29411765	24.96	24.84
24BY06774	7	Male	14.04545455	24.02	24.04
24BY06776	9	Male	13.60606061	23.13	23.32
24BY06777	14	Female	9.24137931	25.82	25.43
24BY06783	7	Female	21	22.78	22.77
24BY06784	9	Male	15.14285714	23.91	23.77
24BY06787	3	Male	15.96	21.29	21.06
24BY06788	7	Female	22.71428571	23.6	23.58
24BY06792	13	Female	14.09677419	24.05	24.03
24BY06794	8	Female	13.29411765	24.31	24.24
24BY06796	8	Female	15.61538462	23.06	23.08
24BY06798	11	Female	9.905660377	24.23	24.04
24BY06939	7	Male	1.905775076	24.18	24.05
24BY06942	7	Male	14.68965517	23.15	23.2
24BY06944	6	Male	14.04545455	24.08	24
24BY06947	8	Male	15.14285714	23.67	23.41
24BY06950	7	Male	15.07407407	24.01	23.86
24BY06953	6	Male	11.95121951	26.19	26.13
24BY06955	8	Female	13.36111111	25.38	25.6
24BY06958	8	Female	26.8	23.64	23.64
24BY06960	5	Female	15.96	22.66	22.56
24BY06963	7	Male	34.5	23.14	23.25
24BY06965	4	Male	26.8	22.81	22.77
24BY06967	8	Male	15.82608696	25.18	26.24
24BY07187	10	Female	15.45833333	28.28	26.57
24BY07189	15	Female	16.3	25.43	25.55

24BY07191	3	Male	13.4	22.45	22.52
24BY07192	10	Male	15.45833333	25.88	26.12
24BY07193	12	Female	15.82608696	26.17	26.02
24BY07197	7	Male	1	24.41	24.25
24BY07230	6	Male	12.89473684	24.56	24.69
24BY07231	5	Male	1	23.22	23.15
24BY07233	10	Female	28	27.81	27.89
24BY07235	5	Male	13.73529412	24.14	24.1
24BY07237	9	Female	13.36111111	24.91	24.97
24BY07243	12	Male	15.82608696	25.62	25.45
24BY07245	11	Male	1.142114385	23.21	23.35
24BY07247	8	Male	1.178378378	24.43	24.43
24BY07248	9	Male	13.60606061	24.21	23.79
24BY07252	5	Male	8.983606557	25.46	25.47
24BY07255	7	Male	13.875	23.54	23.36
24BY07256	10	Male	14.04545455	26.25	26.44
24BY07259	4	Female	15.96	23.57	23.47
24BY07301	9	Male	11.92857143	23.74	23.94
24BY07303	12	Male	10.77083333	25.43	25.66
24BY07308	12	Female	9.905660377	25.69	26.33
24BY07310	7	Male	1	23.12	23.19
24BY07311	12	Female	14.26666667	25.11	25.04
24BY07326	10	Female	13.05405405	23.68	23.47
24BY07329	10	Female	7.243589744	24.1	24.01
24BY07333	13	Female	14.26666667	25.29	25.42
24BY07334	6	Female	13.60606061	22.33	22.47
24BY07336	13	Male	13.8	26.9	26.8
24BY07342	6	Female	7.942857143	23.73	23.71
24BY07345	6	Female	8.378787879	23.78	23.72
24BY07346	11	Male	11.52272727	23.79	23.67
24BY07350	4	Male	2.193661972	22.91	22.79
24BY07354	7	Female	14.21428571	22.65	22.47
24BY07355	13	Female	9.535714286	25.32	25.21
24BY07358	6	Female	26.8	23.19	23.19
24BY07359	7	Female	13.85714286	23.31	23.14
24BY07363	4	Male	15.82608696	24.67	24.67
24BY07366	7	Female	11.92857143	24.68	24.81
24BY07369	8	Female	13.8	25.11	24.25
24BY07372	9	Male	7.929577465	25.58	25.78
24BY07373	21	Male	15.14285714	26.73	25.86
24BY07376	10	Female	13.73529412	24.73	24.85
24BY07379	7	Female	15.82608696	23.08	23.23
24BY07381	8	Male	13.60606061	23.64	23.66
24BY07384	5	Female	8.578125	22.16	22.22
24BY07385	5	Female	24	23.27	23.33

24BY07389	10	Male	9.535714286	24.82	24.69
24BY07392	9	Male	13.8	24.65	24.54
24BY07393	10	Male	9.905660377	25.56	25.61
24BY07400	6	Female	13.60606061	23.86	23.82
24BY07702	14	Female	13.875	25.4	25.71
24BY07705	3	Female	10.9787234	23.69	23.49
24BY07706	3	Male	22.71428571	24.09	24
24BY07711	7	Female	15.82608696	24.22	24.38
24BY07713	10	Male	8.983606557	24.83	24.99
24BY07715	8	Female	10.67346939	22.86	23.35
24BY07716	9	Male	24	24.91	24.89
24BY07720	8	Male	13.73529412	25.22	25.35
24BY07723	14	Female	18	27.35	26.69
24BY08635	7	Female	16.52272727	25.77	25.67
24BY08638	7	Female	11.31111111	22.84	22.98
24BY08641	7	Male	14.68965517	22.62	22.59
24BY08644	6	Male	14.04545455	23.52	23.47
24BY08645	13	Male	15.45833333	26.35	26.55
24BY08647	10	Female	13.29411765	24.54	23.85
24BY08655	8	Male	7.819444444	23.51	23.39
24BY08659	8	Female	14.26666667	24.4	24.36
24BY08661	6	Male	13.85	23.92	24.04
24BY08665	8	Male	15.96	24.25	24.15
24BY08668	6	Female	9.24137931	22.31	22.46
24BY08670	6	Female	13.60606061	22.8	22.6
24BY08702	7	Male	9.403508772	23.7	23.82
24BY08705	8	Male	9.083333333	25.91	25.98
24BY08707	6	Female	15.96	23.97	24.14
24BY08711	7	Female	14.68965517	23.21	23.15
24BY08716	12	Female	7.942857143	24.03	24.21
24BY08719	17	Male	15.82608696	25.8	26.03
24BY08721	13	Male	24	30.13	30.16
24BY08723	6	Male	8.983606557	23.83	23.91
24BY08726	10	Male	12.89473684	25.7	25.72
24BY08729	16	Male	13.73529412	25.7	25.46
24BY08730	10	Male	11.31111111	25.32	25.36
24BY08732	10	Male	11.79069767	24.62	24.46
24BY08734	6	Female	15.96	23.86	23.79
24BY08736	8	Male	15.61538462	25.12	25.19
24BY08739	7	Male	22.83333333	24.71	24.72
24BY08741	10	Female	10.9787234	23.03	23.02
24BY08744	9	Male	22	23.99	23.97
24BY08747	9	Female	15.96	25.09	25.12
24BY08749	5	Female	15.30769231	23.47	23.37
24BY08750	7	Male	9.535714286	23.4	23.26

24BY13502	9	Female	15.82608696	25.71	25.89
24BY13504	8	Female	14.26666667	24.18	24.31
24BY13506	6	Female	11.15217391	24.9	24.96
24BY16011	9	Male	15.07407407	24.2	24.56
24BY16065	8	Male	14.09677419	25.26	25.2
24BY16089	3	Male	13.8	24	22.51
24BY17003	9	Female	12.89473684	23.76	23.79
24BY17004	8	Female	9.814814815	24.04	23.79
24BY17008	7	Female	14.04545455	23.4	23.24
24BY17010	9	Female	15.61538462	24.22	24.22
24BY17016	8	Female	5.586538462	23.7	23.65
24BY17019	14	Female	24.04545455	26.75	26.78
24BY17021	23	Female	22	28.71	28.03
24BY17024	7	Male	1.5599022	26.27	26.51
24BY17101	11	Male	24	26.49	25.4
24BY17106	7	Female	9.814814815	22.75	22.74
24BY17107	4	Male	15.82608696	22.03	22.09
24BY17112	4	Male	15.82608696	21.7	21.47
24BY17115	14	Male	24	25.67	24.73
24BY17118	7	Male	9.814814815	23.98	24.05
24BY17120	14	Female	28.48	27.27	27.1
24BY17122	12	Female	24.04545455	25.94	26.25
24BY17125	11	Female	14.04545455	26.51	26.32
24BY17131	8	Male	22	24.39	24.4
24BY17135	9	Male	12.89473684	26.65	26.33
24BY17139	9	Female	12.89473684	25.12	25.19
24BY17141	11	Female	13.875	26.23	26.97
24BY17144	6	Male	11.79069767	25.43	25.26
24BY17147	7	Female	22	23.55	23.5
24BY17149	9	Female	13.85	25.31	24.26
24BY17152	11	Female	23.36111111	26.45	26.03
24BY17155	10	Male	14.04545455	25.12	25.31
24BY17158	8	Female	13.85	22.98	22.94
24BY17160	4	Male	13.85714286	NA	NA
24BY17163	12	Male	12.38461538	26.88	26.93
24BY17165	11	Male	14.04545455	26.17	26.84
24BY17167	6	Female	7.712328767	23.32	23.53
24BY17170	7	Female	11.92857143	24.07	24.1
24BY17173	6	Female	13.8	21.99	21.89
24BY17176	10	Male	22.36111111	26.32	26.28
24BY17179	5	Female	7.608108108	NA	NA
24BY17182	12	Male	11.92857143	24.38	24.32
24BY17299	6	Male	8.043478261	22.59	22.64
24BY17384	8	Female	14.09677419	24.05	27.26
24BY17386	4	Female	13.73529412	23.98	23.99

24BY17387	5	Male	15.61538462	23.73	24.05
24BY17701	9	Female	15.45833333	25.16	25.23
24BY17703	6	Male	13.36111111	23.47	23.45
24BY17706	9	Male	13.85714286	25.61	24.98
24BY17709	7	Female	6.93902439	21.74	21.98
24BY17712	8	Female	15.82608696	25.23	25.11
24BY17714	9	Male	14.04545455	24.49	24.37
24BY17716	6	Male	15.07407407	23.86	23.88
24BY17717	13	Male	14.26666667	25.99	25.92
24BY17719	8	Male	14.26666667	24.15	24.16
24BY17721	13	Male	15.96	26.29	25.52
24BY17723	5	Male	12.38461538	21.58	21.6
24BY17726	9	Female	11.95121951	25.54	25.55
24BY17729	9	Female	7.506666667	25.27	25.31
24BY17731	11	Female	14.04545455	26.1	26.01
24BY17734	7	Female	21	24.49	24.39
24BY17736	16	Male	14.04545455	27.41	27.7
24BY17738	11	Male	15.45833333	26.31	26.36
24BY17741	11	Male	12.15	27.92	27.44
24BY17743	13	Female	18.71052632	26.35	26.46
24BY17746	13	Female	22.79069767	27.56	27.54
24BY17814	4	Male	12.89473684	22.48	25.6
24BY17815	4	Female	16.63636364	29.11	27.75
24BY17819	4	Female	11.92857143	23.61	23.58
24BY17822	13	Male	23.36111111	29.06	29.23
24BY17825	7	Female	10.77083333	23.25	22.48
24BY17828	3	Female	15.96	22.12	25.22
24BY17832	9	Female	14.04545455	23.86	24.27
24BY17834	4	Female	15.07407407	23.07	22.62
24BY17837	3	Male	13.4	21.65	21.51
24BY17841	8	Female	15.82608696	25.5	25.52
24BY17843	12	Male	13.4	24.01	24.69
24BY17845	9	Male	9.905660377	23.99	24.43
24BY17849	14	Male	9.905660377	26.74	26.43
24BY17852	6	Female	13.85714286	23.75	23.79
24BY17855	10	Female	11.79069767	24.3	24.8
24BY17858	9	Male	9.24137931	25.25	25.47
24BY17859	15	Female	18.13235294	27.17	26.92
24BY17860	11	Female	10.2745098	23.77	23.71
24BY17865	9	Female	13.85	24.07	23.76
24BY17867	15	Male	14.04545455	24.96	25.06
24BY17884	9	Male	13.60606061	25.13	25.04
25BY61001	12	Female	15.96	24.02	23.59
25BY61006	11	Male	13.85	21.93	23.7
25BY61008	11	Female	15.07407407	24.64	24.65

25BY61009	7	Female	9.24137931	23.12	23.34
25BY61011	6	Female	11.95121951	22.97	23.12
25BY61019	9	Female	8.043478261	24.88	24.66
25BY61297	5	Male	10.77083333	23.7	23.38
25BY61807	9	Female	9.220338983	25.08	25.27
25BY61811	9	Female	7.1125	25.92	27.24
25BY61813	8	Female	13.875	23.53	23.57
25BY61815	14	Female	13.4	24.83	24.74
25BY61817	9	Male	13.4	24.24	24.3
25BY61818	11	Female	14.26666667	25.4	25.42
25BY61823	7	Male	13.73529412	23.57	23.35
25BY61825	8	Male	7.929577465	24.58	24.54
25BY61826	7	Female	9.814814815	23.41	25.29
25BY61829	12	Male	14.9	27.04	27.31
25BY61830	16	Female	14.04545455	NA	NA
25BY61832	16	Female	12.89473684	26.67	27.1
25BY61834	7	Male	23.66666667	23.16	23
25BY61837	14	Male	15.82608696	26.1	26.28
25BY61839	8	Female	15.45833333	25.21	25.28
25BY61842	10	Male	23.66666667	25.99	26.03
25BY61844	5	Male	9.220338983	23.8	23.62
25BY61845	10	Male	24	30.23	29.51
25BY61850	10	Male	15.45833333	26.77	26.54
25BY61852	9	Male	15.82608696	28.54	27.95
25BY61854	7	Male	1.728767123	24.32	23.76
25BY61857	12	Male	8.983606557	25.33	25.39
25BY61860	12	Male	24	27.9	28.18
25BY61861	6	Male	7.929577465	25.13	25.02
25BY61865	7	Female	15.45833333	23.94	23.93
25BY61868	3	Male	15.96	21.46	21.37
25BY61871	7	Male	15.45833333	24.39	24.51
25BY61874	7	Male	10.77083333	24.08	24.07
25BY61877	17	Female	17.94285714	27.43	27.49
25BY61879	12	Female	15.45833333	29.22	29.1
25BY61884	13	Male	14.04545455	27.13	26.96
25BY61887	10	Female	15.82608696	24.52	24.08
25BY61889	6	Female	9.905660377	24.26	24.24
25BY61893	7	Female	15.96	24.3	24.37
25BY61895	11	Male	15.07407407	27.04	26.07
25BY61897	27	Female	12.72222222	26.48	26.1
25BY61900	5	Male	15.96	25.54	24.77
25BY61918	7	Female	13.8	23.03	22.97
25BY61921	6	Male	12.15	25.04	24.76
25BY61923	5	Female	11.31111111	23.33	23.46
25BY61927	6	Female	14.09677419	23.54	23.46

25BY61930	7	Male	10.77083333	23.85	23.86
25BY61937	4	Male	13.4	22.84	22.98
25BY61939	8	Male	10.9787234	24.4	24.47
25BY61940	10	Female	14.04545455	25.61	25.53
25BY61943	8	Female	15.61538462	22.78	22.76
25BY61946	4	Female	11.92857143	23.7	23.75
25BY61948	11	Male	14.26666667	24.5	24.57
25BY61950	6	Female	15.82608696	23.27	23.19
25BY61953	6	Male	13.875	26.68	26.84
25BY61954	7	Female	9.905660377	22.32	22.34
25BY61959	11	Male	14.26666667	24.25	24.11
25BY61961	6	Male	13.73529412	25	24.92
25BY61964	8	Male	6.93902439	22.57	22.62
25BY61965	11	Male	11.79069767	25.9	25.32
25BY61969	9	Female	14.04545455	23.76	23.48
25BY61973	7	Female	15.82608696	23.75	23.83
25BY61978	11	Female	15.82608696	26.74	26.68
25BY61981	12	Female	22.71428571	25.2	25.18
25BY61985	5	Female	11.92857143	25.38	25.68
25BY61987	8	Male	15.96	25.19	25.91
25BY61990	8	Female	15.96	23.1	23.06
25BY61993	13	Male	11.92857143	25.91	24.82
25BY61995	9	Male	7.942857143	23.53	23.87
25BY61998	9	Female	14.04545455	24.47	24.62
25BY62000	12	Male	14.04545455	26.19	26.17
25BY63201	11	Female	11.31111111	24.68	24.67
25BY63203	10	Female	15.82608696	23.2	23.18
25BY63206	12	Male	9.814814815	24.6	24.35
25BY63208	11	Female	10.67346939	26.89	27.19
25BY63210	12	Female	22.71428571	26	25.99
25BY63212	8	Male	14.04545455	25.31	25.3
25BY63215	9	Female	12.72222222	23.4	23.55
PSI2550065	10	Female	8.578125	23.22	23.36
PSI2550067	9	Male	14.04545455	26.52	25.97
PSI2550068	31	Female	15.96	28.26	27.41
PSI2550071	6	Male	11.95121951	23.74	23.94
PSI2550072	16	Male	15.61538462	27.22	26.87
PSI2550074	7	Female	16.3	23.92	23.98
PSI2550077	5	Male	9.905660377	22.71	22.54
PSI2550079	10	Female	24	24.85	24.86
PSI2550080	15	Male	11.15217391	25.2	25.36
PSI2550082	10	Female	13.85714286	23.64	23.42
PSI2550085	6	Male	10.67346939	23.83	23.86
PSI2550088	11	Female	11.15217391	25.34	24.93
PSI2550090	8	Male	15.96	24.13	24.01

PSI2550092	7	Female	15.96	22.88	22.83
PSI2550095	8	Male	15.82608696	23.9	23.83
PSI2550097	8	Male	13.73529412	23.15	23.3
PSI2550100	8	Female	15.82608696	22.37	22.56
PSI2550133	11	Female	14.09677419	24.85	25.21
PSI2550136	17	Male	15.07407407	26.92	26.78
PSI2550137	23	Male	12.89473684	25.58	25.74
PSI2550140	5	Female	13.73529412	22.87	24.61
PSI2550143	14	Male	9.905660377	26.67	26.4
PSI2550144	14	Male	15.82608696	28.62	28.63
PSI2550147	15	Female	22.83333333	26.88	27.73
PSI2550150	9	Female	14.09677419	24.47	24.52
PSI2550153	4	Male	15.31818182	23.2	21.74
PSI2550156	10	Male	13.60606061	24.83	24.82
PSI2550159	6	Female	12.15	22.67	22.46
PSI2550162	8	Female	11.79069767	24.6	23.98
PSI2550165	6	Male	13.875	23.96	24.13
PSI2550167	6	Male	14.04545455	23.66	23.56
PSI2550170	14	Female	8.983606557	27.68	27.88
PSI2550173	7	Male	13.85714286	25.53	25.26
PSI2550176	6	Male	16.63636364	24.41	24.37
PSI2550178	5	Male	14.09677419	23.22	22.95
PSI2550181	9	Male	13.85714286	24.73	24.9
PSI2550183	6	Female	12.72222222	22.4	22.23
PSI2550186	11	Female	7.243589744	25.77	26.17
PSI2550188	16	Female	11.79069767	26.85	26.24
PSI2550190	11	Male	13.73529412	26.92	26.37
PSI2550194	5	Female	13.85714286	23.71	23.62
PSI2550196	17	Male	10.9787234	25.74	25.63
PSI2550198	2	Female	1.547215496	21.91	21.85
PSI2550201	8	Female	15.82608696	23.58	23.82
PSI2550203	8	Female	10.67346939	23.91	23.99
PSI2550205	12	Male	9.535714286	25.85	25.95
PSI2550208	12	Male	23.66666667	NA	NA
PSI2550209	10	Female	15.96	26.06	26.4
PSI2550212	12	Female	15.14285714	25.92	25.9
PSI2550215	7	Female	11.92857143	22.4	22.42
PSI2550218	8	Female	13.4	22.96	22.76
PSI2550221	8	Male	14.04545455	24.01	23.79
PSI2550224	7	Male	12.38461538	24.19	24.57
PSI2550226	15	Male	11.92857143	25.03	25.04
PSI2550228	7	Male	15.82608696	25.04	25.04
PSI2550232	8	Male	9.905660377	24.77	24.99
PSI2550236	15	Male	14.09677419	25.54	25.93
PSI2550238	11	Female	14.04545455	25.72	25.85

PSI2550241	4	Male	13.73529412	22.19	23.38
PSI2550244	6	Female	10.67346939	25.01	25.06
PSI2550245	7	Male	34.5	23.38	23.54
PSI2550247	14	Female	11.92857143	25	25.06
PSI2550250	11	Female	14.26666667	24.71	24.81
PSI2550252	11	Male	13.60606061	24.98	24.94
PSI2550255	8	Male	13.60606061	24.02	24.19
PSI2550256	7	Female	9.220338983	24.49	24.56
PSI2550259	8	Male	15.82608696	23.67	23.77
PSI2550262	8	Male	12.89473684	23.24	23.26
PSI2550267	14	Female	26.8	24.89	24.43
PSI2550270	9	Male	15.07407407	24.48	24.6
PSI2550271	13	Female	14.26666667	26.83	26.83
PSI2550277	8	Male	13.8	23.98	23.93
PSI2550279	7	Female	7.506666667	22.67	22.67
PSI2550281	4	Female	15.82608696	23.06	23.19
PSI2550284	28	Female	8.983606557	26.69	27.17
PSI2550287	6	Male	14.68965517	23.97	23.64
PSI2550292	9	Male	15.96	24.06	23.9
PSI2550295	9	Male	14.04545455	23.93	24.03
PSI2550300	8	Female	9.24137931	24.88	24.87
PSI2550304	5	Male	15.96	22.67	22.66
PSI2550307	8	Male	13.73529412	24.18	24.36
PSI2550309	10	Female	13.875	24.25	24.32
PSI2550311	14	Male	13.60606061	24.34	24.34
PSI2550314	9	Male	13.36111111	25.1	25.04
PSI2550316	11	Male	13.85	25.28	25.3
PSI2550318	13	Male	13.05405405	25.68	25.76
PSI2550321	7	Female	10.77083333	24.84	24.86
PSI2550323	9	Female	15.82608696	23.69	23.71
PSI2550325	5	Female	13.85714286	25.07	25.26
PSI2550328	13	Female	15.82608696	24.73	24.74
PSI2550329	12	Male	12.89473684	26.21	26.02
PSI2550332	7	Female	14.04545455	24.56	24.68
PSI2550334	7	Male	9.083333333	23.94	24.03
PSI2550337	11	Female	13.85714286	25.55	25.95
PSI2550339	10	Female	16.63636364	24.73	25.16
PSI2550341	5	Male	11.15217391	22.67	22.57
PSI2550344	11	Male	14.04545455	24.97	25.27
PSI2550346	11	Male	12.89473684	24.06	24.55
PSI2550347	16	Male	12.89473684	27.26	27.42
PSI2550350	15	Male	15.07407407	26.28	25.48
PSI2550353	3	Male	13.85714286	22.27	22.22
PSI2550356	6	Female	13.60606061	23.57	23.46
PSI2550359	8	Male	1.105175292	23.93	23.85

PSI2550362	11	Male	15.96	24.66	24.82
PSI2550363	11	Male	16.3	25.07	25.65
PSI2550368	8	Female	22.71428571	23.84	23.86
PSI2550371	9	Male	22.71428571	25.21	25.23
PSI2550373	9	Male	12.71052632	24.63	24.59
PSI2550375	10	Female	12.72222222	25.5	26.2
PSI2550376	7	Female	14.26666667	23.31	23.4
PSI2550380	7	Female	13.85	23.21	22.5
PSI2550382	13	Male	15.96	26.3	26.74
PSI2550384	14	Male	15.82608696	25.57	25.64
PSI2550386	10	Female	15.96	26.51	26.12
PSI2550389	7	Male	13.36111111	21.51	21.52
PSI2550392	8	Female	13.73529412	24.44	23.94
PSI2550395	7	Female	15.14285714	22.04	22.06
PSI2550397	13	Male	4.13986014	22.41	22.85
PSI2550399	7	Male	15.45833333	22.75	22.67
PSI2550483	8	Female	9.083333333	22.32	22.34
PSI2550486	6	Male	15.45833333	23.6	23.5
PSI2550488	7	Female	13.85	23.38	23.46
PSI2550491	7	Female	13.85	24.43	23.99
PSI2550493	9	Female	15.61538462	22.91	22.81
PSI2550495	7	Female	24	22.28	22.32
PSI2550498	12	Female	21	24.5	24.76
PSI2555101	5	Female	12.15	24.43	24.04
PSI2555104	8	Female	11.15217391	24.67	24.61
PSI2555106	10	Male	32.89473684	27.09	27.5
PSI2555109	18	Female	15.14285714	27.4	27.01
PSI2555110	6	Female	12.71052632	22.72	22.69
PSI2555112	6	Female	10.77083333	23.71	23.68
PSI2555114	9	Female	15.96	24	23.92
PSI2555117	9	Male	13.85	24.45	23.86
PSI2555120	8	Male	15.96	24.9	24.8
PSI2555123	5	Male	14.09677419	22.64	22.53
PSI2555125	9	Female	13.85	25.23	25.4
PSI2555128	4	Male	13.60606061	21.89	21.63
PSI2555131	6	Male	14.26666667	23.48	23.16
PSI2555133	4	Female	13.875	21.7	21.53
PSI2555135	9	Female	24	25.19	25.15
PSI2555139	12	Male	15.61538462	27.87	27.48
PSI2555141	7	Male	9.814814815	22.82	22.78
PSI2555144	5	Female	11.79069767	23.25	23.25
PSI2555147	7	Male	13.4	23.91	23.76
PSI2555150	5	Male	15.96	24.14	24.1
PSI2555153	9	Male	8.132352941	24.46	25.38
PSI2555155	3	Male	13.85714286	25.45	25.39

PSI2555158	3	Male	12.38461538	24.78	24.24
PSI2555161	4	Male	14.26666667	24.79	24.87
PSI2555164	8	Male	23.66666667	25.42	24.84
PSI2555166	14	Male	26.8	25.33	25.53
PSI2555168	6	Female	7.819444444	23.51	23.52
PSI2555170	8	Female	13.05405405	24	24.29
PSI2555173	5	Male	9.24137931	22.93	22.93
PSI2555176	8	Female	13.4	23.6	23.98
PSI2555179	13	Male	16.33333333	25.61	25.44
PSI2555181	8	Male	14.04545455	22.41	22.82
PSI2555184	9	Female	15.96	25.3	25.31
PSI2555188	14	Female	7.407894737	26.7	26.69
PSI2555191	8	Female	13.875	24.5	24.51
PSI2555193	11	Male	14.04545455	26.43	26.33
PSI2555195	7	Male	11.15217391	24.19	24.14
PSI2555197	5	Male	13.875	23.06	23.06
PSI2555200	8	Female	16.33333333	23.32	23.36
PSI2555203	13	Female	10.67346939	26.94	27.26
PSI2555204	12	Female	12.38461538	26.13	25.94
PSI2555207	6	Female	9.814814815	24.01	24.16
PSI2555210	10	Female	9.814814815	23.55	23.95
PSI2555212	9	Female	11.92857143	23.22	23.13
PSI2555216	6	Female	16.63636364	23.23	23.05
PSI2555219	6	Female	13.4	21.75	21.85
PSI2555220	11	Female	16.63636364	24.21	25.61
PSI2555223	8	Female	15.96	23.88	24.21
PSI2555225	9	Female	21	24.12	23.81
PSI2555228	6	Male	11.95121951	24.24	24.11
PSI2555230	7	Male	15.07407407	23.51	23.73
PSI2555233	10	Female	9.905660377	25.34	25.71
PSI2555235	12	Male	11.95121951	27.62	27.73
PSI2555236	18	Female	22.15	28.66	28.09
PSI2555239	9	Female	1.004518072	24.13	24.36
PSI2555241	6	Female	15.82608696	23.47	23.65
PSI2555245	5	Male	11.92857143	24.57	24.54
PSI2555247	13	Male	9.814814815	26.33	25.62
PSI2555249	6	Male	23.66666667	23.97	23.75
PSI2555254	5	Male	14.09677419	23.61	23.59

SE_OD	SE_OS	Sphere_OS	Cylinder_OS	Sphere_OD	Cylinder_OD	SE_min	SE_max
-3.25	-1.875	-1.25	-1.25	-2.75	-1	-3.25	-1.875
-4.5	-4.125	-3.75	-0.75	-4.25	-0.5	-4.5	-4.125
-3	-3	-3	NA	-3	NA	-3	-3
-1.625	-1.5	-1.25	-0.5	-1.25	-0.75	-1.625	-1.5
-6.375	-7.25	-6.75	-1	-5.75	-1.25	-7.25	-6.375
-5	-2.5	-2.5	NA	-5	NA	-5	-2.5
-2.625	-2.25	-1.75	-1	-2.25	-0.75	-2.625	-2.25
-3.5	-4	-3	-2	-3	-1	-4	-3.5
-7.375	-7.125	-5.75	-2.75	-6.25	-2.25	-7.375	-7.125
1	1.25	1.25	NA	1	NA	1	1.25
-7.375	-7.5	-6	-3	-6.25	-2.25	-7.5	-7.375
-11.5	-11	-9.75	-2.5	-10.25	-2.5	-11.5	-11
-7.875	-7	-6.75	-0.5	-7.5	-0.75	-7.875	-7
-5.75	-5.625	-5	-1.25	-5	-1.5	-5.75	-5.625
0.5	0.5	0.5	NA	0.5	NA	0.5	0.5
-7.5	-6.5	-6.5	NA	-7	-1	-7.5	-6.5
1	1.25	1.25	NA	1	NA	1	1.25
0	0	0	NA	0	NA	0	0
-1.5	-1	-1	NA	-1.5	NA	-1.5	-1
-2.75	-2.75	-2.75	NA	-2.75	NA	-2.75	-2.75
-3.375	-3.75	-3.5	-0.5	-3.25	-0.25	-3.75	-3.375
-2.5	-1.5	-1.5	NA	-2.5	NA	-2.5	-1.5
-3.75	-3.75	-3.75	NA	-3.75	NA	-3.75	-3.75
-1.875	2.5	0.75	3.5	-0.25	-3.25	-1.875	2.5
-4.25	-4.25	-3.75	-1	-4.25	NA	-4.25	-4.25
-2	-2	-2	NA	-2	NA	-2	-2
-1.375	-2.125	-1.75	-0.75	-1.25	-0.25	-2.125	-1.375
-3.25	-3	-2.25	-1.5	-3	-0.5	-3.25	-3
-8	-3.375	-2.5	-1.75	-6	-4	-8	-3.375
-3	-3.25	-3	-0.5	-2.75	-0.5	-3.25	-3
-1.875	-1.625	-1.25	-0.75	-1.75	-0.25	-1.875	-1.625
-6.5	-6.125	-5.25	-1.75	-5.75	-1.5	-6.5	-6.125
-3.125	-2.875	-2.5	-0.75	-2.75	-0.75	-3.125	-2.875
-0.375	-0.25	0	-0.5	0	-0.75	-0.375	-0.25
-1.75	-0.5	0	-1	-1.5	-0.5	-1.75	-0.5
-1.25	-1.375	-1	-0.75	-1	-0.5	-1.375	-1.25
-4.375	-4.25	-3.5	-1.5	-4	-0.75	-4.375	-4.25
-9.5	-8.25	-7.25	-2	-8.5	-2	-9.5	-8.25
0.25	0.25	0.5	-0.5	0.5	-0.5	0.25	0.25
1	1	1	NA	1	NA	1	1
0.25	-0.5	0.25	-1.5	0.75	-1	-0.5	0.25
-3	-4.5	-4	-1	-2	-2	-4.5	-3
-1.625	-1.5	-1.5	NA	-1.5	-0.25	-1.625	-1.5

-1.125	-1.125	-0.75	-0.75	-0.75	-0.75	-1.125	-1.125
-3.625	-3.5	-3	-1	-3.25	-0.75	-3.625	-3.5
-6	-5.75	-5.5	-0.5	-5.75	-0.5	-6	-5.75
-0.125	-0.125	0	-0.25	0	-0.25	-0.125	-0.125
-1.125	-1.375	-1	-0.75	-1	-0.25	-1.375	-1.125
-6.25	-6.125	-5.5	-1.25	-6	-0.5	-6.25	-6.125
-2	-2.125	-1.75	-0.75	-1.75	-0.5	-2.125	-2
-6.125	-8.75	-6.75	-4	-4.75	-2.75	-8.75	-6.125
-6.625	-7.625	-6.5	-2.25	-5.5	-2.25	-7.625	-6.625
-5.5	-5.5	-5.5	NA	-5.5	NA	-5.5	-5.5
-1.75	-1.375	-1	-0.75	-1.75	NA	-1.75	-1.375
-3.75	-4	-4	NA	-3.75	NA	-4	-3.75
-2.39	-0.5	-0.5	NA	-2.25	-0.28	-2.39	-0.5
-3.25	-3.125	-2.25	-1.75	-2.75	-1	-3.25	-3.125
-3.125	-3.125	-2.5	-1.25	-2.75	-0.75	-3.125	-3.125
-3	-3	-2.75	-0.5	-2.75	-0.5	-3	-3
-4.75	-6.875	-6.25	-1.25	-4	-1.5	-6.875	-4.75
-2.375	-2.25	-2	-0.5	-2	-0.75	-2.375	-2.25
-3.25	-3	-3	NA	-3.25	NA	-3.25	-3
-1.75	-1.5	-0.5	-2	-0.5	-2.5	-1.75	-1.5
-0.5	-0.25	-0.25	NA	-0.5	NA	-0.5	-0.25
-2	-2.5	-2.25	-0.5	-1.75	-0.5	-2.5	-2
-1.5	-1.125	-0.5	-1.25	-1	-1	-1.5	-1.125
-1.125	-0.625	1	-3.25	0.5	-3.25	-1.125	-0.625
-6.375	-6.5	-5.5	-2	-5.75	-1.25	-6.5	-6.375
-2	-2.25	-2.25	NA	-2	NA	-2.25	-2
0.25	0.25	0.25	NA	0.25	NA	0.25	0.25
-5.625	-6.625	-6	-1.25	-5	-1.25	-6.625	-5.625
-1	-0.5	-0.5	NA	-1	NA	-1	-0.5
-4.75	-4.875	-4	-1.75	-4.25	-1	-4.875	-4.75
-7.25	-7.75	-7.75	NA	-7.25	NA	-7.75	-7.25
-1.625	-1.625	-1.25	-0.75	-1.25	-0.75	-1.625	-1.625
-5.75	-17.5	-16	-3	-4.25	-3	-17.5	-5.75
-5	-4	-3.5	-1	-4.5	-1	-5	-4
-3.125	-2.875	-2.25	-1.25	-2.75	-0.75	-3.125	-2.875
-4.125	-3.875	-3.25	-1.25	-3.5	-1.25	-4.125	-3.875
-0.5	-0.5	-0.25	-0.5	-0.25	-0.5	-0.5	-0.5
-5.125	-5.125	-4.5	-1.25	-4.5	-1.25	-5.125	-5.125
-2.5	-2.5	-2.5	NA	-2.5	NA	-2.5	-2.5
-6.625	-7.125	-6.75	-0.75	-6.25	-0.75	-7.125	-6.625
-6.375	-6.125	-5.5	-1.25	-5.75	-1.25	-6.375	-6.125
-1.875	-2	-1.75	-0.5	-1.5	-0.75	-2	-1.875
-4.25	-4.25	-4.25	NA	-4.25	NA	-4.25	-4.25
-7	-6.5	-6.25	-0.5	-6.75	-0.5	-7	-6.5
-0.25	-0.25	0	-0.5	0	-0.5	-0.25	-0.25

-1.25	-1.25	-1	-0.5	-1	-0.5	-1.25	-1.25
-2.5	-1.875	-1.5	-0.75	-2	-1	-2.5	-1.875
-6.5	-6.875	-6	-1.75	-5.75	-1.5	-6.875	-6.5
-9.25	-7	-6.25	-1.5	-9	-0.5	-9.25	-7
-7.125	-7.125	-6.25	-1.75	-6.5	-1.25	-7.125	-7.125
-1.5	-1.5	-1.5	NA	-1.5	NA	-1.5	-1.5
0.25	0.25	0.25	NA	0.25	NA	0.25	0.25
0.5	-0.125	0.25	-0.75	1	-1	-0.125	0.5
-5	-4.75	-4.5	-0.5	-5	NA	-5	-4.75
-8.75	-8.25	-7.5	-1.5	-8.5	-0.5	-8.75	-8.25
-1.625	-1.875	-1.5	-0.75	-1.25	-0.75	-1.875	-1.625
-2.25	-3	-2.25	-1.5	-1.75	-1	-3	-2.25
-4	-3.875	-3	-1.75	-2.75	-2.5	-4	-3.875
-2.5	-2.25	-2.25	NA	-2.5	NA	-2.5	-2.25
-1.75	-1.375	-0.75	-1.25	-1	-1.5	-1.75	-1.375
0.25	0.5	3	-5	2.75	-5	0.25	0.5
-4.5	-3.625	-3.25	-0.75	-4.5	NA	-4.5	-3.625
-6.5	-4.375	-3.75	-1.25	-6	-1	-6.5	-4.375
-1.25	-0.25	NA	-0.5	-1	-0.5	-1.25	-0.25
-4.5	-4	-3.5	-1	-4.25	-0.5	-4.5	-4
-0.5	-0.25	-0.25	NA	-0.5	NA	-0.5	-0.25
0.5	0.5	0.75	-0.5	0.75	-0.5	0.5	0.5
-8	-6	-5	-2	-7.25	-1.5	-8	-6
0	0	0	NA	0	NA	0	0
-0.5	-0.5	-0.25	-0.5	-0.25	-0.5	-0.5	-0.5
5.25	6	6	NA	5.25	NA	5.25	6
-0.125	-0.125	0	-0.25	0	-0.25	-0.125	-0.125
-3.25	-2.75	-2.25	-1	-2.75	-1	-3.25	-2.75
-1.5	-1	-1	NA	-1.5	NA	-1.5	-1
-0.25	-0.375	0	-0.75	0	-0.5	-0.375	-0.25
-3	-2.75	-2.5	-0.5	-2.75	-0.5	-3	-2.75
-3.25	-2.75	-2.5	-0.5	-3	-0.5	-3.25	-2.75
-1.25	-1	-1	NA	-1	-0.5	-1.25	-1
0.75	0.75	0.75	NA	0.75	NA	0.75	0.75
-1.125	-0.875	-0.75	-0.25	-1	-0.25	-1.125	-0.875
-0.5	-0.25	0	-0.5	-0.25	-0.5	-0.5	-0.25
-6	-6	-5.25	-1.5	-5.25	-1.5	-6	-6
-6.25	-6.5	-5.5	-2	-5	-2.5	-6.5	-6.25
-2.875	-2.625	-2.5	-0.25	-2.75	-0.25	-2.875	-2.625
-1.25	-1.5	-1.5	NA	-1.25	NA	-1.5	-1.25
-0.875	-1	-0.75	-0.5	-0.75	-0.25	-1	-0.875
0.25	0.25	0.25	NA	0.25	NA	0.25	0.25
-3.5	-5.5	-5	-1	-2.75	-1.5	-5.5	-3.5
-11.375	-7.125	-6.25	-1.75	-10	-2.75	-11.375	-7.125
-5.5	-5.875	-5.5	-0.75	-5.5	NA	-5.875	-5.5

1	1	1	NA	1	NA	1	1
-5.375	-6.375	-6	-0.75	-5	-0.75	-6.375	-5.375
-5.25	-5.25	-4.75	-1	-5.25	NA	-5.25	-5.25
-1.75	-1.75	-1.75	NA	-1.75	NA	-1.75	-1.75
-2.75	-2.75	-2.5	-0.5	-2.5	-0.5	-2.75	-2.75
0.5	0.25	0.25	NA	0.5	NA	0.25	0.5
-8.25	-8	-7.75	-0.5	-8	-0.5	-8.25	-8
-3	-3	-3	NA	-2.75	-0.5	-3	-3
-2.25	-2.25	-2	-0.5	-2.25	NA	-2.25	-2.25
-6.25	-5.25	-4.75	-1	-5.75	-1	-6.25	-5.25
1.25	1	1	NA	1.25	NA	1	1.25
-0.75	-0.5	-0.25	-0.5	-0.5	-0.5	-0.75	-0.5
-1	-0.5	0	-1	-0.5	-1	-1	-0.5
-10	-9.75	-9.25	-1	-9.75	-0.5	-10	-9.75
-1.25	-1.125	-0.75	-0.75	-1	-0.5	-1.25	-1.125
-3	-3.25	-3.25	NA	-3	NA	-3.25	-3
-0.5	-0.75	0	-1.5	0	-1	-0.75	-0.5
-1.5	-1.875	-1.75	-0.25	-1.25	-0.5	-1.875	-1.5
-3.875	-4.375	-4	-0.75	-3.5	-0.75	-4.375	-3.875
-6	-7.5	-7	-1	-5.5	-1	-7.5	-6
0.375	0.25	-0.25	1	0	0.75	0.25	0.375
-7.75	-7.375	-6.75	-1.25	-7.25	-1	-7.75	-7.375
-0.875	-0.375	0	-0.75	-0.5	-0.75	-0.875	-0.375
-2.25	-2.125	-2	-0.25	-2.25	NA	-2.25	-2.125
-8.125	-9.375	-7.5	-3.75	-6.75	-2.75	-9.375	-8.125
-0.875	-1.5	-0.75	-1.5	0	-1.75	-1.5	-0.875
-7.625	-7.25	-6.25	-2	-6.5	-2.25	-7.625	-7.25
-1	-1	-1	NA	-1	NA	-1	-1
-2.5	-2.5	-2.25	-0.5	-2.25	-0.5	-2.5	-2.5
-0.75	-0.25	-0.25	NA	-0.75	NA	-0.75	-0.25
1.5	1.5	1.5	NA	1.5	NA	1.5	1.5
-0.625	-0.625	-0.5	-0.25	-0.5	-0.25	-0.625	-0.625
-4.6	-4.375	-4	-0.75	-4	-1.2	-4.6	-4.375
0.25	0.25	0.25	NA	0.25	NA	0.25	0.25
-1.5	-1.25	-1.25	NA	-1.5	NA	-1.5	-1.25
-2	-2	-3	2	-3	2	-2	-2
-3.125	-3.375	-3	-0.75	-3	-0.25	-3.375	-3.125
-4.75	-2.75	-1.25	-3	-4.25	-1	-4.75	-2.75
-1.5	-1.5	-1.5	NA	-1.5	NA	-1.5	-1.5
-9.25	-7.375	-6.75	-1.25	-8.5	-1.5	-9.25	-7.375
-2.25	-2	-2	NA	-2.25	NA	-2.25	-2
-1.75	-2	-1.75	-0.5	-1.5	-0.5	-2	-1.75
0	0.25	0.25	NA	0	NA	0	0.25
0	0	0	NA	0	NA	0	0
0.5	0.5	0.5	NA	0.5	NA	0.5	0.5

-3.25	-2.875	-2.25	-1.25	-2.75	-1	-3.25	-2.875
-1.5	-1.5	-1.5	NA	-1.5	NA	-1.5	-1.5
-4.875	-4.875	-4.5	-0.75	-4.75	-0.25	-4.875	-4.875
-2	-2	-2	NA	-2	NA	-2	-2
-6.75	-7.25	-5.75	-3	-5.25	-3	-7.25	-6.75
-1	-1	-1	NA	-1	NA	-1	-1
-0.75	-0.25	0	-0.5	0	-1.5	-0.75	-0.25
-2.5	-2.75	-2.5	-0.5	-2.5	NA	-2.75	-2.5
-2	-2.5	-2	-1	-1.5	-1	-2.5	-2
-2	-2.75	-2.5	-0.5	-2	NA	-2.75	-2
-2.125	-2.5	-2	-1	-1.75	-0.75	-2.5	-2.125
-2.75	-3.25	-3	-0.5	-2.5	-0.5	-3.25	-2.75
-5	-4	-4	NA	-5	NA	-5	-4
-6.5	-5.875	-5	-1.75	-6	-1	-6.5	-5.875
-0.75	-1.25	-1	-0.5	-0.5	-0.5	-1.25	-0.75
-1.5	-1.5	-1	-1	-1	-1	-1.5	-1.5
-1.75	-2	-1.75	-0.5	-1.5	-0.5	-2	-1.75
-5.875	-6	-5.75	-0.5	-5.5	-0.75	-6	-5.875
-0.75	0	0	NA	-0.75	NA	-0.75	0
-0.5	-0.5	-0.5	NA	-0.5	NA	-0.5	-0.5
-1.75	-1.5	-1.5	NA	-1.75	NA	-1.75	-1.5
-0.125	-0.625	-0.5	-0.25	0	-0.25	-0.625	-0.125
-3.75	-3.75	-3.25	-1	-3.5	-0.5	-3.75	-3.75
-0.25	-0.25	0.75	-2	1.25	-3	-0.25	-0.25
-2.25	-1.75	-1.25	-1	-1.75	-1	-2.25	-1.75
-3	-3.25	-3.25	NA	-3	NA	-3.25	-3
-5	-4.75	-4.75	NA	-4.75	-0.5	-5	-4.75
-1.25	-1.25	-0.25	-2	-0.25	-2	-1.25	-1.25
-3.25	-3.25	-3	-0.5	-3.25	NA	-3.25	-3.25
-2.25	-2.625	-1.5	-2.25	-1	-2.5	-2.625	-2.25
-4.375	-6.25	-5	-2.5	-3.75	-1.25	-6.25	-4.375
-14	-14	-14	NA	-14	NA	-14	-14
0	-2.25	-2.25	NA	0	NA	-2.25	0
-1.5	-1.25	-1.25	NA	-1.5	NA	-1.5	-1.25
-4.25	-3.5	-3.5	NA	-4.25	NA	-4.25	-3.5
-7	-7	-7	NA	-7	NA	-7	-7
-1.5	-1.25	-1	-0.5	-1.5	NA	-1.5	-1.25
-1	-1	-1	NA	-1	NA	-1	-1
-1.5	-1.5	-1.5	NA	-1.5	NA	-1.5	-1.5
-2.5	-2.75	-2.25	-1	-2.25	-0.5	-2.75	-2.5
-1.75	-1.5	-1.5	NA	-1.75	NA	-1.75	-1.5
-2	-2	-2	NA	-2	NA	-2	-2
-4.25	-4.25	-3.25	-2	-3.25	-2	-4.25	-4.25
-1.5	-1.25	-1	-0.5	-1.25	-0.5	-1.5	-1.25
-1.25	-1.25	-1.25	NA	-1.25	NA	-1.25	-1.25

-6.75	-6.5	-5.25	-2.5	-5.5	-2.5	-6.75	-6.5
-3	-3.25	-3.25	NA	-3	NA	-3.25	-3
-3.5	-3	-2.25	-1.5	-2.75	-1.5	-3.5	-3
-1.875	-2.5	-2	-1	-1	-1.75	-2.5	-1.875
-3.5	-3.25	-3.25	NA	-3.25	-0.5	-3.5	-3.25
-5.75	-2	-1.5	-1	-5.5	-0.5	-5.75	-2
-3.25	-2.75	-2.25	-1	-2.75	-1	-3.25	-2.75
-2	-1.25	-1.25	NA	-2	NA	-2	-1.25
0.25	0.25	0.5	-0.5	0.25	NA	0.25	0.25
-2	-2	-2	NA	-2	NA	-2	-2
-1.75	-2.25	-2.25	NA	-1.75	NA	-2.25	-1.75
-9.125	-9.25	-8.75	-1	-8.75	-0.75	-9.25	-9.125
-9.25	-8.75	-8.75	NA	-9.25	NA	-9.25	-8.75
-8.5	-8.75	-7.25	-3	-7.25	-2.5	-8.75	-8.5
-5.75	-2.75	-2.5	-0.5	-5.5	-0.5	-5.75	-2.75
-0.5	-0.75	-0.75	NA	-0.5	NA	-0.75	-0.5
0	0.375	0	0.75	-0.5	1	0	0.375
-1.5	-1.25	-0.5	-1.5	-0.5	-2	-1.5	-1.25
-6.75	-4.5	-3	-3	-6	-1.5	-6.75	-4.5
-2.25	-2.25	-2.25	NA	-2.25	NA	-2.25	-2.25
-11.875	-11.875	-11.25	-1.25	-11.25	-1.25	-11.875	-11.875
-10	-11	-9	-4	-7.75	-4.5	-11	-10
-7	-7.625	-8.5	1.75	-7.75	1.5	-7.625	-7
-2.25	-2	-2	NA	-2.25	NA	-2.25	-2
-6.25	-5.75	-5.25	-1	-6	-0.5	-6.25	-5.75
-5.5	-6.375	-5.75	-1.25	-5	-1	-6.375	-5.5
-7.25	-8.75	-7.75	-2	-6.5	-1.5	-8.75	-7.25
-7.75	-7.875	-7	-1.75	-6.25	-3	-7.875	-7.75
-2.5	-2.375	-2.25	-0.25	-2.25	-0.5	-2.5	-2.375
-4.25	-1.875	-1.25	-1.25	-3.75	-1	-4.25	-1.875
-7.25	-6.5	-5.5	-2	-7	-0.5	-7.25	-6.5
-2.75	-2.875	-2.25	-1.25	-2	-1.5	-2.875	-2.75
-2.25	-2	-1.75	-0.5	-2	-0.5	-2.25	-2
-3.75	-3.5	-3.25	-0.5	-3.5	-0.5	-3.75	-3.5
-0.75	-0.75	-0.75	NA	-0.75	NA	-0.75	-0.75
-5.75	-5.75	-5.5	-0.5	-5.5	-0.5	-5.75	-5.75
-1	-1.5	-1.25	-0.5	-0.75	-0.5	-1.5	-1
-4.75	-4.75	-4.75	NA	-4.5	-0.5	-4.75	-4.75
0.75	1	1	NA	0.5	0.5	0.75	1
-6.75	-6.75	-7.75	2	-7.75	2	-6.75	-6.75
-4.25	-4.25	-4	-0.5	-4	-0.5	-4.25	-4.25
0.25	0.25	0.25	NA	0.25	NA	0.25	0.25
0.75	0.75	0.75	NA	0.75	NA	0.75	0.75
-2.75	-11.375	-11	-0.75	-2.5	-0.5	-11.375	-2.75
-1.75	-1.75	-1.5	-0.5	-1.5	-0.5	-1.75	-1.75

-3.25	-3.625	-2.5	-2.25	-2	-2.5	-3.625	-3.25
-3	-2.625	-3	0.75	-3.25	0.5	-3	-2.625
0.5	0.5	0.5	NA	0.5	NA	0.5	0.5
-3.75	-2.25	-2	-0.5	-3.25	-1	-3.75	-2.25
-1.625	-1.375	-0.75	-1.25	-0.5	-2.25	-1.625	-1.375
-1.75	-1.5	-1.25	-0.5	-1.5	-0.5	-1.75	-1.5
-1.25	-1	-1	NA	-1.25	NA	-1.25	-1
-1.75	-2	-2	NA	-1.75	NA	-2	-1.75
-4.625	-4.375	-3.75	-1.25	-4.25	-0.75	-4.625	-4.375
-3	-2.75	-2.5	-0.5	-2.75	-0.5	-3	-2.75
-4.625	-3.5	-1.5	-4	-3.25	-2.75	-4.625	-3.5
-0.25	-0.25	-1.25	2	-1	1.5	-0.25	-0.25
-5.875	-5.75	-5.75	NA	-5.5	-0.75	-5.875	-5.75
-5.625	-5.75	-5.25	-1	-5.25	-0.75	-5.75	-5.625
-5.75	-5.25	-5.25	NA	-5.5	-0.5	-5.75	-5.25
-2.25	-2.5	-2	-1	-1.75	-1	-2.5	-2.25
-7.25	-8	-7.5	-1	-6.5	-1.5	-8	-7.25
-5.625	-6	-6.25	0.5	-6.25	1.25	-6	-5.625
-8.25	-8	-7.5	-1	-8	-0.5	-8.25	-8
-7.25	-8.75	-7.75	-2	-6.5	-1.5	-8.75	-7.25
-12.625	-12.75	-11	-3.5	-11.25	-2.75	-12.75	-12.625
-1	-12.75	-11.5	-2.5	-0.5	-1	-12.75	-1
-8.5	-8	-6.5	-3	-7.5	-2	-8.5	-8
-1.5	-1.5	-1.75	0.5	-1.5	NA	-1.5	-1.5
-13.75	-13.25	-11	-4.5	-11.75	-4	-13.75	-13.25
-4.5	-4.5	-6	3	-5.25	1.5	-4.5	-4.5
-1.125	-11.125	-10.5	-1.25	-0.5	-1.25	-11.125	-1.125
-16.75	-16.5	-16	-1	-16	-1.5	-16.75	-16.5
-3	-1.625	-3	2.75	-4.5	3	-3	-1.625
0.25	1	0.75	0.5	0	0.5	0.25	1
-3.5	-3.5	-3.5	NA	-3.25	-0.5	-3.5	-3.5
-2.5	-3.75	-3.5	-0.5	-2.25	-0.5	-3.75	-2.5
-1.875	-3.125	-2.5	-1.25	-1	-1.75	-3.125	-1.875
-7.75	-7.25	-6.5	-1.5	-7	-1.5	-7.75	-7.25
-2.625	-2.875	-2.5	-0.75	-2.25	-0.75	-2.875	-2.625
-5.375	-7.875	-7.25	-1.25	-5	-0.75	-7.875	-5.375
-4.25	-4.625	-4	-1.25	-3.75	-1	-4.625	-4.25
-10.125	-9.375	-8.25	-2.25	-9	-2.25	-10.125	-9.375
-5.375	-4.75	-4.75	NA	-5	-0.75	-5.375	-4.75
-1.375	-1.125	0	-2.25	-0.5	-1.75	-1.375	-1.125
-3.25	-3.5	-3	-1	-3	-0.5	-3.5	-3.25
-4.875	-4.375	-4.25	-0.25	-4.75	-0.25	-4.875	-4.375
-2.375	-1.125	-0.75	-0.75	-2	-0.75	-2.375	-1.125
5.125	0.75	1	-0.5	5.75	-1.25	0.75	5.125
-2.5	-2.25	-2	-0.5	-2.5	NA	-2.5	-2.25

-2.25	-2.5	-2	-1	-1.75	-1	-2.5	-2.25
-0.5	-0.625	-0.25	-0.75	0	-1	-0.625	-0.5
-1.625	-1.25	0	-2.5	-0.5	-2.25	-1.625	-1.25
-0.25	-0.25	-0.25	NA	-0.25	NA	-0.25	-0.25
-3.125	-3.5	-2.5	-2	-2.5	-1.25	-3.5	-3.125
-5.75	-9.5	-8.75	-1.5	-5.25	-1	-9.5	-5.75
-0.75	-0.75	-0.75	NA	-0.75	NA	-0.75	-0.75
-5.75	-5.625	-5	-1.25	-5.25	-1	-5.75	-5.625
-1.5	-1.75	-1.75	NA	-1.5	NA	-1.75	-1.5
-4.125	-4.125	-3.5	-1.25	-3.5	-1.25	-4.125	-4.125
-0.25	0	0	NA	-0.25	NA	-0.25	0
-0.375	-0.375	0	-0.75	0	-0.75	-0.375	-0.375
-3.125	-8.75	-6.75	-4	-1.75	-2.75	-8.75	-3.125
-9.375	-10.25	-10	-0.5	-9	-0.75	-10.25	-9.375
-9.75	-11.25	-10.75	-1	-9.5	-0.5	-11.25	-9.75
-7.5	-8.5	-7	-3	-6.25	-2.5	-8.5	-7.5
-1	-0.625	-0.5	-0.25	-1	NA	-1	-0.625
-5.25	-5.75	-5.75	NA	-5.25	NA	-5.75	-5.25
-4.625	-4.75	-4.25	-1	-4.25	-0.75	-4.75	-4.625
-7	-7.125	-6.5	-1.25	-6.5	-1	-7.125	-7
-1.5	-0.875	0	-1.75	0.25	-3.5	-1.5	-0.875
-14.375	-13.375	-12.5	-1.75	-13.5	-1.75	-14.375	-13.375
-5.5	-5	-4.75	-0.5	-5.25	-0.5	-5.5	-5
-8.625	-6.75	-5.25	-3	-7	-3.25	-8.625	-6.75
-1.75	-1	-0.5	-1	-1.5	-0.5	-1.75	-1
-5.875	-5.75	-5.25	-1	-5.75	-0.25	-5.875	-5.75
-6.75	-7	-6.75	-0.5	-6.75	NA	-7	-6.75
-3.875	-3.625	-3	-1.25	-3	-1.75	-3.875	-3.625
-2.5	-2.375	-3	1.25	-3	1	-2.5	-2.375
0	-0.75	0	-1.5	0.25	-0.5	-0.75	0
2.625	2.125	2	0.25	2.5	0.25	2.125	2.625
-3.75	-3.75	-3.75	NA	-3.75	NA	-3.75	-3.75
-11.25	-11.875	-11	-1.75	-10.25	-2	-11.875	-11.25
-10.875	-10.875	-9.5	-2.75	-9.5	-2.75	-10.875	-10.875
-7.75	-7	-6.5	-1	-7	-1.5	-7.75	-7
-5.625	-4.375	-3.75	-1.25	-5	-1.25	-5.625	-4.375
-2.25	-2.5	-2.25	-0.5	-2.25	NA	-2.5	-2.25
-2.375	-2.375	-2.75	0.75	-2.75	0.75	-2.375	-2.375
-7.125	-4.625	-3.75	-1.75	-6.25	-1.75	-7.125	-4.625
-5	-4.25	-4.25	NA	-5	NA	-5	-4.25
-1.5	0	0	NA	-1.5	NA	-1.5	0
-1	-0.75	-0.5	-0.5	-1	NA	-1	-0.75
-1	-1	-1	NA	-1	NA	-1	-1
-1.125	-1.25	-1.25	NA	-1	-0.25	-1.25	-1.125
-1.375	-1.125	-0.75	-0.75	-1	-0.75	-1.375	-1.125

-2.25	-1.75	-1.75	NA	-2	-0.5	-2.25	-1.75
0	0	0	NA	0	NA	0	0
-1	-1.25	-0.5	-1.5	-0.5	-1	-1.25	-1
-6.125	-6	-4	-4	-4.25	-3.75	-6.125	-6
-3.75	-3.625	-2.75	-1.75	-3	-1.5	-3.75	-3.625
-1.25	-1.125	-1	-0.25	-1	-0.5	-1.25	-1.125
-3.5	-3.375	-2.75	-1.25	-3	-1	-3.5	-3.375
0	0	0	NA	0	NA	0	0
-11.25	-12.25	-10.25	-4	-9.25	-4	-12.25	-11.25
0	0	0	NA	0	NA	0	0
-2.625	-2	-1.5	-1	-2	-1.25	-2.625	-2
-3.5	-3.25	-2.25	-2	-2.5	-2	-3.5	-3.25
-0.375	-0.5	0	-1	0	-0.75	-0.5	-0.375
-6	-4.25	-3.5	-1.5	-5.5	-1	-6	-4.25
-3.375	-2.375	-1.25	-2.25	-1.75	-3.25	-3.375	-2.375
-1.75	-2	-2	NA	-1.75	NA	-2	-1.75
-7.75	-7.875	-7	-1.75	-7	-1.5	-7.875	-7.75
-6.125	-6.625	-5.25	-2.75	-5	-2.25	-6.625	-6.125
-5.75	-6.25	-5	-2.5	-4	-3.5	-6.25	-5.75
-4.25	-6.125	-4.5	-3.25	-2.5	-3.5	-6.125	-4.25
-1	-0.625	-0.25	-0.75	-1	NA	-1	-0.625
-7.125	-4.75	-3.5	-2.5	-6.25	-1.75	-7.125	-4.75
-0.375	-1.125	-1	-0.25	-0.25	-0.25	-1.125	-0.375
-2.375	-2.875	-2.25	-1.25	-2	-0.75	-2.875	-2.375
-4.125	-3.875	-3	-1.75	-3.25	-1.75	-4.125	-3.875
-2.25	-2.25	-2.25	NA	-2.25	NA	-2.25	-2.25
-1.25	-1.125	-0.75	-0.75	-1.25	NA	-1.25	-1.125
-2	-1.5	-1.5	NA	-2	NA	-2	-1.5
-7.625	-8.375	-7.5	-1.75	-6.5	-2.25	-8.375	-7.625
-8.25	-7.5	-7	-1	-7.75	-1	-8.25	-7.5
-2.875	-3	-2.5	-1	-2.5	-0.75	-3	-2.875
-4.5	-5	-4.75	-0.5	-4.5	NA	-5	-4.5
-1.875	-2.125	-2	-0.25	-1.75	-0.25	-2.125	-1.875
-7.5	-6	-5.25	-1.5	-7.25	-0.5	-7.5	-6
-10.75	-9	-8.75	-0.5	-10.75	NA	-10.75	-9
-0.375	-0.75	-0.5	-0.5	0	-0.75	-0.75	-0.375
-10.875	-9.75	-9	-1.5	-10	-1.75	-10.875	-9.75
-1.5	-1.25	-1.25	NA	-1.5	NA	-1.5	-1.25
-0.625	-0.5	0	-1	0	-1.25	-0.625	-0.5
-3.75	-4	-3	-2	-3	-1.5	-4	-3.75
-2	-2.375	-2	-0.75	-1.5	-1	-2.375	-2
-2	-1.75	-1.25	-1	-1.5	-1	-2	-1.75
-4.125	-3.75	-3.25	-1	-3.5	-1.25	-4.125	-3.75
-4	-3.125	-2.25	-1.75	-3.75	-0.5	-4	-3.125
-1.5	-0.875	-0.75	-0.25	-1.25	-0.5	-1.5	-0.875

-0.5	-0.75	-0.75	NA	-0.5	NA	-0.75	-0.5
-1	-1	-1	NA	-1	NA	-1	-1
-0.75	-1.125	-0.75	-0.75	-0.25	-1	-1.125	-0.75
0.25	-0.5	-0.25	-0.5	0	0.5	-0.5	0.25
-6	-6.5	-5.75	-1.5	-5	-2	-6.5	-6
-9.125	-8.5	-7.75	-1.5	-8	-2.25	-9.125	-8.5
-5.5	-5.25	-5	-0.5	-5	-1	-5.5	-5.25
0.25	-5.25	-5	-0.5	0	0.5	-5.25	0.25
-8.5	-7.75	-7.25	-1	-8.25	-0.5	-8.5	-7.75
-11	-11	-9	-4	-9.25	-3.5	-11	-11
-7.5	-10	-8.25	-3.5	-6.75	-1.5	-10	-7.5
-4.25	-4.25	-4.5	0.5	-4.25	NA	-4.25	-4.25
6.75	4.75	4.5	0.5	6.5	0.5	4.75	6.75
-4.5	-4.75	-4.5	-0.5	-4.5	NA	-4.75	-4.5
-2	-1.25	-1	-0.5	-2	NA	-2	-1.25
-4.125	-2.625	-2	-1.25	-3.75	-0.75	-4.125	-2.625
-1.875	-2	-1.25	-1.5	-1	-1.75	-2	-1.875
-1	-0.75	-0.75	NA	-1	NA	-1	-0.75
-3.125	-3.25	-3	-0.5	-2.75	-0.75	-3.25	-3.125
-3.75	-3.5	-3.25	-0.5	-3.5	-0.5	-3.75	-3.5
-2.25	-2.25	-2	-0.5	-2	-0.5	-2.25	-2.25
-0.75	-0.625	-1	0.75	-0.75	NA	-0.75	-0.625
-0.75	-1	-1	NA	-0.75	NA	-1	-0.75
-0.375	-0.375	0	-0.75	0	-0.75	-0.375	-0.375
-3.875	-4.875	-4.5	-0.75	-3.5	-0.75	-4.875	-3.875
-8.875	-7.25	-6.75	-1	-8.5	-0.75	-8.875	-7.25
-2.75	-1.375	-1	-0.75	-2.5	-0.5	-2.75	-1.375
-3.625	-3.625	-3	-1.25	-3.25	-0.75	-3.625	-3.625
-1.125	-1.5	-0.75	-1.5	-0.5	-1.25	-1.5	-1.125
3	3	3	NA	3	NA	3	3
-3.375	-2.875	-2	-1.75	-2.25	-2.25	-3.375	-2.875
-1.75	-1.375	-1	-0.75	-1.5	-0.5	-1.75	-1.375
-3.875	-3.875	-3	-1.75	-3.5	-0.75	-3.875	-3.875
-4.5	-4.5	-4	-1	-4.25	-0.5	-4.5	-4.5
-3.25	-3.75	-3.5	-0.5	-3.25	NA	-3.75	-3.25
-5.25	-5.5	-5.25	-0.5	-5.25	NA	-5.5	-5.25
-0.75	-1	-1	NA	-0.75	NA	-1	-0.75
-0.625	-0.5	-0.25	-0.5	-0.25	-0.75	-0.625	-0.5
-1.5	-1.25	-1.25	NA	-1.5	NA	-1.5	-1.25
-0.75	-1.875	-1.75	-0.25	-0.75	NA	-1.875	-0.75
-5.375	-5.625	-4.75	-1.75	-4.75	-1.25	-5.625	-5.375
-5.75	-5.25	-4.5	-1.5	-4.75	-2	-5.75	-5.25
-3.75	-4.5	-2.75	-3.5	-2	-3.5	-4.5	-3.75
-8.875	-10.125	-8.25	-3.75	-7.25	-3.25	-10.125	-8.875
-2.75	-3.25	-3.25	NA	-2.5	-0.5	-3.25	-2.75

-1.375	-4.125	-3	-2.25	-0.5	-1.75	-4.125	-1.375
-2.75	-2.75	-2.75	NA	-2.75	NA	-2.75	-2.75
-2.25	-2.5	-2.25	-0.5	-2	-0.5	-2.5	-2.25
-6.125	-6.5	-6	-1	-5.75	-0.75	-6.5	-6.125
-5	-4.5	-3.75	-1.5	-4.25	-1.5	-5	-4.5
-2	-2	-2	NA	-2	NA	-2	-2
-1.5	-2.25	-2.25	NA	-1.5	NA	-2.25	-1.5
-5.5	-5.5	-4	-3	-4	-3	-5.5	-5.5
0.25	0.25	0.25	NA	0.25	NA	0.25	0.25
-0.5	-0.25	-0.25	NA	-0.5	NA	-0.5	-0.25
-6.125	-5.25	-4.25	-2	-5.75	-0.75	-6.125	-5.25
-0.5	-1	-0.75	-0.5	-0.5	NA	-1	-0.5
-7	-7	-6	-2	-6	-2	-7	-7
-3	-3.25	-3	-0.5	-3	NA	-3.25	-3
-1.25	-1.25	-1.25	NA	-1.25	NA	-1.25	-1.25
-1.875	-2.375	-1.75	-1.25	-1.5	-0.75	-2.375	-1.875
-7.25	-8.75	-8.75	NA	-6.5	-1.5	-8.75	-7.25
-1.75	-1.5	-1.25	-0.5	-1.75	NA	-1.75	-1.5
-1.375	-1.375	-1.25	-0.25	-1.25	-0.25	-1.375	-1.375
-0.75	-0.75	-0.75	NA	-0.75	NA	-0.75	-0.75
-2.75	-2.5	-2.5	NA	-2.75	NA	-2.75	-2.5
-2.25	-2	-1.75	-0.5	-2	-0.5	-2.25	-2
-2.25	-1.875	-1.75	-0.25	-2	-0.5	-2.25	-1.875
-5.5	-5.25	-4.75	-1	-5	-1	-5.5	-5.25
-4.25	-4.25	-3.75	-1	-3.75	-1	-4.25	-4.25
-1	-1	-1	NA	-1	NA	-1	-1
-6	-6.5	-6.25	-0.5	-6	NA	-6.5	-6
-1.75	-2.5	-2.5	NA	-1.75	NA	-2.5	-1.75
-2	-2.5	-2.5	NA	-2	NA	-2.5	-2
-0.75	-1	-0.75	-0.5	-0.5	-0.5	-1	-0.75
-5.75	-6.75	-6.75	NA	-5.75	NA	-6.75	-5.75
-4.25	-4	-3.75	-0.5	-4	-0.5	-4.25	-4
-2	-2.125	-1.75	-0.75	-1.75	-0.5	-2.125	-2
-4	-4.75	-4.5	-0.5	-3.5	-1	-4.75	-4
-1.75	-1.75	-1.75	NA	-1.75	NA	-1.75	-1.75
-4.375	-4.375	-3.75	-1.25	-3.75	-1.25	-4.375	-4.375
-3.5	-4.625	-4	-1.25	-2.75	-1.5	-4.625	-3.5
-0.75	-0.5	0	-1	-0.25	-1	-0.75	-0.5
-8.125	-8.375	-7	-2.75	-6.5	-3.25	-8.375	-8.125
-2.5	-2.75	-1.75	-2	-1.5	-2	-2.75	-2.5
-6.375	-6.5	-6	-1	-5.75	-1.25	-6.5	-6.375
-6	-4.375	-3.75	-1.25	-5.25	-1.5	-6	-4.375
0	0	0	NA	0	NA	0	0
-4.125	-4.25	-4	-0.5	-3.75	-0.75	-4.25	-4.125
-0.5	-0.5	-0.5	NA	-0.5	NA	-0.5	-0.5

-2.25	-2.5	-2.5	NA	-2.25	NA	-2.5	-2.25
-4.875	-6	-5	-2	-3.75	-2.25	-6	-4.875
-1.25	-1	-1	NA	-1.25	NA	-1.25	-1
-4	-3.5	-3.5	NA	-3.75	-0.5	-4	-3.5
-2.375	-1.875	-1.25	-1.25	-2	-0.75	-2.375	-1.875
-7	-9.125	-8.25	-1.75	-6.25	-1.5	-9.125	-7
-0.75	-1	-1	NA	-0.75	NA	-1	-0.75
-2.25	-0.75	-0.5	-0.5	-2	-0.5	-2.25	-0.75
-6.25	-7.25	-6.5	-1.5	-5.5	-1.5	-7.25	-6.25
-4.75	-4.875	-4.5	-0.75	-4.5	-0.5	-4.875	-4.75
-7.75	-7	-5	-4	-6.75	-2	-7.75	-7
3.125	2.625	4.25	-3.25	4.75	-3.25	2.625	3.125
-6	-4.5	-3.75	-1.5	-5.25	-1.5	-6	-4.5
0	0	0	NA	0	NA	0	0
4.875	3.5	4.75	-2.5	5.5	-1.25	3.5	4.875
0	0.375	1	-1.25	0.5	-1	0	0.375
0.5	0.5	0.5	NA	0.5	NA	0.5	0.5
-1.75	-1.75	-1.25	-1	-1.5	-0.5	-1.75	-1.75
-2.25	-2.375	-1.25	-2.25	-1.25	-2	-2.375	-2.25
-4	-3.125	-2.25	-1.75	-3.75	-0.5	-4	-3.125
-0.375	-0.375	-0.25	-0.25	-0.25	-0.25	-0.375	-0.375
-0.125	-0.375	-0.25	-0.25	0	-0.25	-0.375	-0.125
-5	-5.5	-5.25	-0.5	-4.75	-0.5	-5.5	-5
-8.75	-7.75	-5.75	-4	-8	-1.5	-8.75	-7.75
-5	-5	-4.5	-1	-4.75	-0.5	-5	-5
-10.5	-11.5	-8.75	-5.5	-7.75	-5.5	-11.5	-10.5
-13.25	-11.875	-11	-1.75	-12	-2.5	-13.25	-11.875
0	0	0	NA	0	NA	0	0
-1.25	-1	-1	NA	-1.25	NA	-1.25	-1
-2.75	-2.5	-2.25	-0.5	-2.5	-0.5	-2.75	-2.5
-3.125	-1.625	-1.25	-0.75	-2.75	-0.75	-3.125	-1.625
0.25	0.25	NA	0.5	NA	0.5	0.25	0.25
0.5	0.5	0.5	NA	0.5	NA	0.5	0.5
-4.625	-4.625	-5	0.75	-5	0.75	-4.625	-4.625
-1.375	-1.25	-1	-0.5	-1	-0.75	-1.375	-1.25
-2	-1	0	-2	-1.25	-1.5	-2	-1
-0.375	-0.125	0.5	-1.25	0	-0.75	-0.375	-0.125
-3.75	-3.75	-3.5	-0.5	-3.5	-0.5	-3.75	-3.75
-7.75	-6.75	-6.25	-1	-7.5	-0.5	-7.75	-6.75
-2.25	-2.5	-2.5	NA	-2.25	NA	-2.5	-2.25
-1.75	-1.5	-1.5	NA	-1.75	NA	-1.75	-1.5
-1	-1	-0.75	-0.5	-1	NA	-1	-1
-1.5	-1.625	-1.5	-0.25	-1.25	-0.5	-1.625	-1.5
-3.625	-5.375	-4.75	-1.25	-3	-1.25	-5.375	-3.625
-7	-6.625	-6.25	-0.75	-6.75	-0.5	-7	-6.625

-7	-5.875	-6.5	1.25	-7.5	1	-7	-5.875
-2.875	-2.875	-2.5	-0.75	-2.5	-0.75	-2.875	-2.875
-0.5	-0.125	0	-0.25	-0.5	NA	-0.5	-0.125
-4.5	-4.75	-4	-1.5	-3.75	-1.5	-4.75	-4.5
-1.25	-1.25	-1.25	NA	-1.25	NA	-1.25	-1.25
-4.125	-4.375	-4	-0.75	-4	-0.25	-4.375	-4.125
0.5	0.75	0	1.5	0	1	0.5	0.75
-1.5	-2.25	-1.75	-1	-0.75	-1.5	-2.25	-1.5
-2.875	-2.125	-1.75	-0.75	-2.5	-0.75	-2.875	-2.125
-0.375	-1.125	-0.75	-0.75	-0.25	-0.25	-1.125	-0.375
-6.25	-6.375	-5.25	-2.25	-5	-2.5	-6.375	-6.25
-8	-8.25	-7.75	-1	-8	NA	-8.25	-8
-3.125	-3.125	-3	-0.25	-3	-0.25	-3.125	-3.125
-2	-1.875	-1.5	-0.75	-1.75	-0.5	-2	-1.875
-2.5	-2.5	-2.5	NA	-2.5	NA	-2.5	-2.5
0	-0.125	-0.75	1.25	-0.25	0.5	-0.125	0
-0.25	0	-1.5	3	-2	3.5	-0.25	0
-9	-9.75	-9	-1.5	-8.5	-1	-9.75	-9
-7.75	-8.125	-7.25	-1.75	-7	-1.5	-8.125	-7.75
-1.875	-1.5	-1.5	NA	-1.75	-0.25	-1.875	-1.5
-1.75	-2.5	-2.5	NA	-1.75	NA	-2.5	-1.75
-0.75	-1.125	-0.75	-0.75	-0.75	NA	-1.125	-0.75
-1.875	-1.875	-1.5	-0.75	-1.5	-0.75	-1.875	-1.875
-1.625	-1.625	-1.25	-0.75	-1.25	-0.75	-1.625	-1.625
-5.25	-7.625	-7	-1.25	-5.25	NA	-7.625	-5.25
-2.875	-3.5	-3.5	NA	-2.5	-0.75	-3.5	-2.875
-3.625	-2.875	-2.5	-0.75	-2.5	-2.25	-3.625	-2.875
-1.5	-1.25	-1	-0.5	-1.25	-0.5	-1.5	-1.25
-1.25	-1.5	-1.5	NA	-1	-0.5	-1.5	-1.25
-5	-5.75	-5.25	-1	-4.5	-1	-5.75	-5
-8	-8.25	-7.75	-1	-7.5	-1	-8.25	-8
-11.5	-10.875	-9.5	-2.75	-10.5	-2	-11.5	-10.875
-1	-1.5	-1.25	-0.5	-0.75	-0.5	-1.5	-1
-1.5	-1.5	-1.25	-0.5	-1.25	-0.5	-1.5	-1.5
-3	-2.5	-2.25	-0.5	-2.75	-0.5	-3	-2.5
-4.75	-3.875	-3.5	-0.75	-4.5	-0.5	-4.75	-3.875
-1.25	-0.075	0	-0.15	-1	-0.5	-1.25	-0.075
-1	-1	-1	NA	-1	NA	-1	-1

SE_mean	AL_mean	Sphere_mean	Cyl_mean	AL_asym	SE_asym	MyopiaGroup
-2.5625	23.22	-2	-1.125	0.5	1.375	Myopia
-4.3125	25.675	-4	-0.625	0.23	0.375	Myopia
-3	25.18	-3	NA	0.02	0	Myopia
-1.5625	23.57	-1.25	-0.625	0.1	0.125	Myopia
-6.8125	26.925	-6.25	-1.125	0.37	0.875	HM
-3.75	24.445	-3.75	NA	0.97	2.5	Myopia
-2.4375	25.26	-2	-0.875	0.56	0.375	Myopia
-3.75	24.925	-3	-1.5	0.09	0.5	Myopia
-7.25	26.75	-6	-2.5	0.08	0.25	HM
1.125	22.07	1.125	NA	0	0.25	Myopia
-7.4375	27.21	-6.125	-2.625	0.12	0.125	HM
-11.25	26.795	-10	-2.5	0.23	0.5	EHM
-7.4375	26.775	-7.125	-0.625	0.29	0.875	HM
-5.6875	24.745	-5	-1.375	0.03	0.125	Myopia
0.5	22.73	0.5	NA	0.16	0	Myopia
-7	25.945	-6.75	-1	0.11	1	HM
1.125	22.15	1.125	NA	0.06	0.25	Myopia
0	22.985	0	NA	0.09	0	Myopia
-1.25	23.495	-1.25	NA	0.05	0.5	Myopia
-2.75	24.5	-2.75	NA	0.02	0	Myopia
-3.5625	NA	-3.375	-0.375	NA	0.375	Myopia
-2	23.815	-2	NA	0.49	1	Myopia
-3.75	25.985	-3.75	NA	0.05	0	Myopia
0.3125	23.945	0.25	0.125	0.45	4.375	Myopia
-4.25	26.34	-4	-1	0	0	Myopia
-2	23.48	-2	NA	0	0	Myopia
-1.75	24.38	-1.5	-0.5	0.14	0.75	Myopia
-3.125	23.88	-2.625	-1	0.22	0.25	Myopia
-5.6875	21.955	-4.25	-2.875	1.13	4.625	HM
-3.125	23.29	-2.875	-0.5	0.06	0.25	Myopia
-1.75	23.45	-1.5	-0.5	0.12	0.25	Myopia
-6.3125	25.64	-5.5	-1.625	0.18	0.375	HM
-3	23.435	-2.625	-0.75	0.19	0.25	Myopia
-0.3125	24.015	0	-0.625	0.01	0.125	Myopia
-1.125	22.685	-0.75	-0.75	0.67	1.25	Myopia
-1.3125	23.175	-1	-0.625	0.11	0.125	Myopia
-4.3125	25.69	-3.75	-1.125	0.14	0.125	Myopia
-8.875	25.38	-7.875	-2	0.54	1.25	HM
0.25	22.825	0.5	-0.5	0.09	0	Myopia
1	21.865	1	NA	0.03	0	Myopia
-0.125	22.625	0.5	-1.25	0.15	0.75	Myopia
-3.75	24.01	-3	-1.5	0.54	1.5	Myopia
-1.5625	23.105	-1.5	-0.25	0.07	0.125	Myopia

-1.125	27.145	-0.75	-0.75	0.07	0	Myopia
-3.5625	25.21	-3.125	-0.875	0.02	0.125	Myopia
-5.875	25.625	-5.625	-0.5	0.01	0.25	HM
-0.125	23.41	0	-0.25	0.18	0	Myopia
-1.25	23.295	-1	-0.5	0.13	0.25	Myopia
-6.1875	27.05	-5.75	-0.875	0.24	0.125	HM
-2.0625	22.965	-1.75	-0.625	0.01	0.125	Myopia
-7.4375	25.095	-5.75	-3.375	0.53	2.625	HM
-7.125	25.58	-6	-2.25	0.78	1	HM
-5.5	26.365	-5.5	NA	0.03	0	Myopia
-1.5625	24.32	-1.375	-0.75	0.1	0.375	Myopia
-3.875	23.88	-3.875	NA	0.14	0.25	Myopia
-1.445	23.345	-1.375	-0.28	0.43	1.89	Myopia
-3.1875	24.19	-2.5	-1.375	0.2	0.125	Myopia
-3.125	24.69	-2.625	-1	0.06	0	Myopia
-3	24.645	-2.75	-0.5	0.05	0	Myopia
-5.8125	26.695	-5.125	-1.375	1.07	2.125	HM
-2.3125	24.21	-2	-0.625	0	0.125	Myopia
-3.125	24.735	-3.125	NA	0.15	0.25	Myopia
-1.625	23.545	-0.5	-2.25	0.07	0.25	Myopia
-0.375	23.495	-0.375	NA	0.13	0.25	Myopia
-2.25	24.89	-2	-0.5	0.34	0.5	Myopia
-1.3125	23.185	-0.75	-1.125	0.09	0.375	Myopia
-0.875	22.875	0.75	-3.25	0.11	0.5	Myopia
-6.4375	25.61	-5.625	-1.625	0.06	0.125	HM
-2.125	25.025	-2.125	NA	0.23	0.25	Myopia
0.25	23.735	0.25	NA	0.19	0	Myopia
-6.125	25.205	-5.5	-1.25	0.25	1	HM
-0.75	23.465	-0.75	NA	0.13	0.5	Myopia
-4.8125	25.71	-4.125	-1.375	0.2	0.125	Myopia
-7.5	26.405	-7.5	NA	0.05	0.5	HM
-1.625	25.075	-1.25	-0.75	0.05	0	Myopia
-11.625	28.355	-10.125	-3	4.63	11.75	EHM
-4.5	26.31	-4	-1	0.28	1	Myopia
-3	24.825	-2.5	-1	0.43	0.25	Myopia
-4	24.98	-3.375	-1.25	0.04	0.25	Myopia
-0.5	23.4	-0.25	-0.5	0.08	0	Myopia
-5.125	25.265	-4.5	-1.25	0.11	0	Myopia
-2.5	24.25	-2.5	NA	0.26	0	Myopia
-6.875	26.07	-6.5	-0.75	0.3	0.5	HM
-6.25	26.005	-5.625	-1.25	0.15	0.25	HM
-1.9375	24.77	-1.625	-0.625	0.1	0.125	Myopia
-4.25	25.905	-4.25	NA	0.11	0	Myopia
-6.75	25.94	-6.5	-0.5	0.28	0.5	HM
-0.25	23.405	0	-0.5	0.03	0	Myopia

-1.25	24.56	-1	-0.5	0.04	0	Myopia
-2.1875	23.135	-1.75	-0.875	0.13	0.625	Myopia
-6.6875	24.32	-5.875	-1.625	0.14	0.375	HM
-8.125	26.995	-7.625	-1	1.41	2.25	HM
-7.125	27.75	-6.375	-1.5	0.28	0	HM
-1.5	22.89	-1.5	NA	0.04	0	Myopia
0.25	22.305	0.25	NA	0.09	0	Myopia
0.1875	24.945	0.625	-0.875	0.45	0.625	Myopia
-4.875	25.125	-4.75	-0.5	0.01	0.25	Myopia
-8.5	28.175	-8	-1	0.43	0.5	HM
-1.75	23.285	-1.375	-0.75	0.09	0.25	Myopia
-2.625	24.21	-2	-1.25	0.22	0.75	Myopia
-3.9375	24.06	-2.875	-2.125	0.3	0.125	Myopia
-2.375	26.05	-2.375	NA	0.2	0.25	Myopia
-1.5625	22.515	-0.875	-1.375	0.07	0.375	Myopia
0.375	23.115	2.875	-5	0.07	0.25	Myopia
-4.0625	25.6	-3.875	-0.75	0.3	0.875	Myopia
-5.4375	25.03	-4.875	-1.125	1	2.125	HM
-0.75	23.005	-1	-0.5	0.47	1	Myopia
-4.25	24.9	-3.875	-0.75	0.12	0.5	Myopia
-0.375	24.03	-0.375	NA	0.02	0.25	Myopia
0.5	23.225	0.75	-0.5	0.19	0	Myopia
-7	25.625	-6.125	-1.75	0.39	2	HM
0	22.775	0	NA	0.01	0	Myopia
-0.5	23.84	-0.25	-0.5	0.14	0	Myopia
5.625	21.175	5.625	NA	0.23	0.75	Myopia
-0.125	23.59	0	-0.25	0.02	0	Myopia
-3	24.04	-2.5	-1	0.02	0.5	Myopia
-1.25	24.275	-1.25	NA	0.07	0.5	Myopia
-0.3125	23.07	0	-0.625	0.02	0.125	Myopia
-2.875	24.135	-2.625	-0.5	0.19	0.25	Myopia
-3	24.115	-2.75	-0.5	0.13	0.5	Myopia
-1.125	23.175	-1	-0.5	0.05	0.25	Myopia
0.75	24.04	0.75	NA	0.08	0	Myopia
-1	23.54	-0.875	-0.25	0.26	0.25	Myopia
-0.375	23.935	-0.125	-0.5	0.15	0.25	Myopia
-6	26.16	-5.25	-1.5	0.06	0	HM
-6.375	25.49	-5.25	-2.25	0.22	0.25	HM
-2.75	23.64	-2.625	-0.25	0	0.25	Myopia
-1.375	22.61	-1.375	NA	0.1	0.25	Myopia
-0.9375	23.195	-0.75	-0.375	0.11	0.125	Myopia
0.25	22.79	0.25	NA	0.04	0	Myopia
-4.5	25.71	-3.875	-1.25	1.06	2	Myopia
-9.25	27.425	-8.125	-2.25	1.71	4.25	EHM
-5.6875	25.49	-5.5	-0.75	0.12	0.375	Myopia

1	22.485	1	NA	0.07	0	Myopia
-5.875	26	-5.5	-0.75	0.24	1	HM
-5.25	26.095	-5	-1	0.15	0	Myopia
-1.75	24.33	-1.75	NA	0.16	0	Myopia
-2.75	24.625	-2.5	-0.5	0.13	0	Myopia
0.375	23.185	0.375	NA	0.07	0.25	Myopia
-8.125	27.85	-7.875	-0.5	0.08	0.25	HM
-3	24.12	-2.875	-0.5	0.04	0	Myopia
-2.25	24.94	-2.125	-0.5	0.06	0	Myopia
-5.75	25.535	-5.25	-1	0.17	1	HM
1.125	23.28	1.125	NA	0.14	0.25	Myopia
-0.625	24.43	-0.375	-0.5	0	0.25	Myopia
-0.75	24	-0.25	-1	0.42	0.5	Myopia
-9.875	25.465	-9.5	-0.75	0.01	0.25	EHM
-1.1875	23.45	-0.875	-0.625	0.18	0.125	Myopia
-3.125	26.345	-3.125	NA	0.19	0.25	Myopia
-0.625	23.52	0	-1.25	0.1	0.25	Myopia
-1.6875	23.84	-1.5	-0.375	0.2	0.375	Myopia
-4.125	25.545	-3.75	-0.75	0.23	0.5	Myopia
-6.75	26.01	-6.25	-1	0.64	1.5	HM
0.3125	23.155	-0.125	0.875	0.07	0.125	Myopia
-7.5625	25.075	-7	-1.125	0.07	0.375	HM
-0.625	23.575	-0.25	-0.75	0.21	0.5	Myopia
-2.1875	24.055	-2.125	-0.25	0.09	0.125	Myopia
-8.75	25.355	-7.125	-3.25	0.13	1.25	HM
-1.1875	22.4	-0.375	-1.625	0.14	0.625	Myopia
-7.4375	26.85	-6.375	-2.125	0.1	0.375	HM
-1	23.72	-1	NA	0.02	0	Myopia
-2.5	23.75	-2.25	-0.5	0.06	0	Myopia
-0.5	23.73	-0.5	NA	0.12	0.5	Myopia
1.5	22.85	1.5	NA	0.12	0	Myopia
-0.625	22.56	-0.5	-0.25	0.18	0	Myopia
-4.4875	25.265	-4	-0.975	0.11	0.225	Myopia
0.25	23.19	0.25	NA	0	0	Myopia
-1.375	23.225	-1.375	NA	0.17	0.25	Myopia
-2	24.67	-3	2	0	0	Myopia
-3.25	24.745	-3	-0.5	0.13	0.25	Myopia
-3.75	24.68	-2.75	-2	0.86	2	Myopia
-1.5	25.68	-1.5	NA	0.2	0	Myopia
-8.3125	26.295	-7.625	-1.375	0.87	1.875	HM
-2.125	24.79	-2.125	NA	0.12	0.25	Myopia
-1.875	23.155	-1.625	-0.5	0.15	0.25	Myopia
0.125	23.65	0.125	NA	0.02	0.25	Myopia
0	22.19	0	NA	0.06	0	Myopia
0.5	23.3	0.5	NA	0.06	0	Myopia

-3.0625	24.755	-2.5	-1.125	0.13	0.375	Myopia
-1.5	24.595	-1.5	NA	0.11	0	Myopia
-4.875	25.585	-4.625	-0.5	0.05	0	Myopia
-2	23.84	-2	NA	0.04	0	Myopia
-7	25.555	-5.5	-3	0.31	0.5	HM
-1	23.59	-1	NA	0.2	0	Myopia
-0.5	24.045	0	-1	0.09	0.5	Myopia
-2.625	24.3	-2.5	-0.5	0.16	0.25	Myopia
-2.25	24.91	-1.75	-1	0.16	0.5	Myopia
-2.375	23.105	-2.25	-0.5	0.49	0.75	Myopia
-2.3125	24.9	-1.875	-0.875	0.02	0.375	Myopia
-3	25.285	-2.75	-0.5	0.13	0.5	Myopia
-4.5	27.02	-4.5	NA	0.66	1	Myopia
-6.1875	25.72	-5.5	-1.375	0.1	0.625	HM
-1	22.91	-0.75	-0.5	0.14	0.5	Myopia
-1.5	22.605	-1	-1	0.03	0	Myopia
-1.875	23.495	-1.625	-0.5	0.05	0.25	Myopia
-5.9375	26.45	-5.625	-0.625	0.2	0.125	HM
-0.375	24.195	-0.375	NA	0.69	0.75	Myopia
-0.5	23.45	-0.5	NA	0.12	0	Myopia
-1.625	24.38	-1.625	NA	0.04	0.25	Myopia
-0.375	23.98	-0.25	-0.25	0.12	0.5	Myopia
-3.75	24.2	-3.375	-0.75	0.1	0	Myopia
-0.25	22.385	1	-2.5	0.15	0	Myopia
-2	22.7	-1.5	-1	0.2	0.5	Myopia
-3.125	23.76	-3.125	NA	0.12	0.25	Myopia
-4.875	25.945	-4.75	-0.5	0.07	0.25	Myopia
-1.25	24.055	-0.25	-2	0.17	0	Myopia
-3.25	23.18	-3.125	-0.5	0.06	0	Myopia
-2.4375	24.12	-1.25	-2.375	0.18	0.375	Myopia
-5.3125	25.915	-4.375	-1.875	0.23	1.875	HM
-14	30.145	-14	NA	0.03	0	EHM
-1.125	23.87	-1.125	NA	0.08	2.25	Myopia
-1.375	25.71	-1.375	NA	0.02	0.25	Myopia
-3.875	25.58	-3.875	NA	0.24	0.75	Myopia
-7	25.34	-7	NA	0.04	0	HM
-1.375	24.54	-1.25	-0.5	0.16	0.25	Myopia
-1	23.825	-1	NA	0.07	0	Myopia
-1.5	25.155	-1.5	NA	0.07	0	Myopia
-2.625	24.715	-2.25	-0.75	0.01	0.25	Myopia
-1.625	23.025	-1.625	NA	0.01	0.25	Myopia
-2	23.98	-2	NA	0.02	0	Myopia
-4.25	25.105	-3.25	-2	0.03	0	Myopia
-1.375	23.42	-1.125	-0.5	0.1	0.25	Myopia
-1.25	23.33	-1.25	NA	0.14	0	Myopia

-6.625	25.8	-5.375	-2.5	0.18	0.25	HM
-3.125	24.245	-3.125	NA	0.13	0.25	Myopia
-3.25	24.93	-2.5	-1.5	0.06	0.5	Myopia
-2.1875	24.38	-1.5	-1.375	0.36	0.625	Myopia
-3.375	25.23	-3.25	-0.5	0.06	0.25	Myopia
-3.875	23.255	-3.5	-0.75	1.49	3.75	Myopia
-3	23.775	-2.5	-1	0.03	0.5	Myopia
-1.625	23.915	-1.625	NA	0.25	0.75	Myopia
0.25	23.32	0.375	-0.5	0.16	0	Myopia
-2	24.22	-2	NA	0	0	Myopia
-2	23.675	-2	NA	0.05	0.5	Myopia
-9.1875	26.765	-8.75	-0.875	0.03	0.125	HM
-9	28.37	-9	NA	0.68	0.5	HM
-8.625	26.39	-7.25	-2.75	0.24	0.25	HM
-4.25	25.945	-4	-0.5	1.09	3	Myopia
-0.625	22.745	-0.625	NA	0.01	0.25	Myopia
0.1875	22.06	-0.25	0.875	0.06	0.375	Myopia
-1.375	21.585	-0.5	-1.75	0.23	0.25	Myopia
-5.625	25.2	-4.5	-2.25	0.94	2.25	HM
-2.25	24.015	-2.25	NA	0.07	0	Myopia
-11.875	27.185	-11.25	-1.25	0.17	0	EHM
-10.5	26.095	-8.375	-4.25	0.31	1	EHM
-7.3125	26.415	-8.125	1.625	0.19	0.625	HM
-2.125	24.395	-2.125	NA	0.01	0.25	Myopia
-6	26.49	-5.625	-0.75	0.32	0.5	HM
-5.9375	25.155	-5.375	-1.125	0.07	0.875	HM
-8	26.6	-7.125	-1.75	0.74	1.5	HM
-7.8125	25.345	-6.625	-2.375	0.17	0.125	HM
-2.4375	23.525	-2.25	-0.375	0.05	0.125	Myopia
-3.0625	24.785	-2.5	-1.125	1.05	2.375	Myopia
-6.875	26.24	-6.25	-1.25	0.42	0.75	HM
-2.8125	25.215	-2.125	-1.375	0.19	0.125	Myopia
-2.125	22.96	-1.875	-0.5	0.04	0.25	Myopia
-3.625	NA	-3.375	-0.5	NA	0.25	Myopia
-0.75	26.905	-0.75	NA	0.05	0	Myopia
-5.75	26.505	-5.5	-0.5	0.67	0	Myopia
-1.25	23.425	-1	-0.5	0.21	0.5	Myopia
-4.75	24.085	-4.625	-0.5	0.03	0	Myopia
0.875	21.94	0.75	0.5	0.1	0.25	Myopia
-6.75	26.3	-7.75	2	0.04	0	HM
-4.25	NA	-4	-0.5	NA	0	Myopia
0.25	24.35	0.25	NA	0.06	0	Myopia
0.75	22.615	0.75	NA	0.05	0	Myopia
-7.0625	25.655	-6.75	-0.625	3.21	8.625	EHM
-1.75	23.985	-1.5	-0.5	0.01	0	Myopia

-3.4375	23.89	-2.25	-2.375	0.32	0.375	Myopia
-2.8125	25.195	-3.125	0.625	0.07	0.375	Myopia
0.5	23.46	0.5	NA	0.02	0	Myopia
-3	25.295	-2.625	-0.75	0.63	1.5	Myopia
-1.5	21.86	-0.625	-1.75	0.24	0.25	Myopia
-1.625	25.17	-1.375	-0.5	0.12	0.25	Myopia
-1.125	24.43	-1.125	NA	0.12	0.25	Myopia
-1.875	23.87	-1.875	NA	0.02	0.25	Myopia
-4.5	25.955	-4	-1	0.07	0.25	Myopia
-2.875	24.155	-2.625	-0.5	0.01	0.25	Myopia
-4.0625	25.905	-2.375	-3.375	0.77	1.125	Myopia
-0.25	21.59	-1.125	1.75	0.02	0	Myopia
-5.8125	25.545	-5.625	-0.75	0.01	0.125	Myopia
-5.6875	25.29	-5.25	-0.875	0.04	0.125	Myopia
-5.5	26.055	-5.375	-0.5	0.09	0.5	Myopia
-2.375	24.44	-1.875	-1	0.1	0.25	Myopia
-7.625	27.555	-7	-1.25	0.29	0.75	HM
-5.8125	26.335	-6.25	0.875	0.05	0.375	HM
-8.125	27.68	-7.75	-0.75	0.48	0.25	HM
-8	26.405	-7.125	-1.75	0.11	1.5	HM
-12.6875	27.55	-11.125	-3.125	0.02	0.125	EHM
-6.875	24.04	-6	-1.75	3.12	11.75	EHM
-8.25	28.43	-7	-2.5	1.36	0.5	HM
-1.5	23.595	-1.625	0.5	0.03	0	Myopia
-13.5	29.145	-11.375	-4.25	0.17	0.5	EHM
-4.5	22.865	-5.625	2.25	0.77	0	Myopia
-6.125	23.67	-5.5	-1.25	3.1	10	EHM
-16.625	24.065	-16	-1.25	0.41	0.25	EHM
-2.3125	22.845	-3.75	2.875	0.45	1.375	Myopia
0.625	21.58	0.375	0.5	0.14	0.75	Myopia
-3.5	25.51	-3.375	-0.5	0.02	0	Myopia
-3.125	24.35	-2.875	-0.5	0.68	1.25	Myopia
-2.5	24.21	-1.75	-1.5	0.44	1.25	Myopia
-7.5	26.585	-6.75	-1.5	0.31	0.5	HM
-2.75	23.77	-2.375	-0.75	0.04	0.25	Myopia
-6.625	24.55	-6.125	-1	0.5	2.5	HM
-4.4375	25.36	-3.875	-1.125	0.22	0.375	Myopia
-9.75	27.045	-8.625	-2.25	0.25	0.75	EHM
-5.0625	23.74	-4.875	-0.75	0.06	0.625	Myopia
-1.25	23.915	-0.25	-2	0.31	0.25	Myopia
-3.375	25.01	-3	-0.75	0.1	0.25	Myopia
-4.625	25.085	-4.5	-0.25	0.09	0.5	Myopia
-1.75	23.805	-1.375	-0.75	0.43	1.25	Myopia
2.9375	22.815	3.375	-0.875	1.77	4.375	Myopia
-2.375	24.645	-2.25	-0.5	0.01	0.25	Myopia

-2.375	23.23	-1.875	-1	0.22	0.25	Myopia
-0.5625	23.045	-0.125	-0.875	0.15	0.125	Myopia
-1.4375	24.77	-0.25	-2.375	0.22	0.375	Myopia
-0.25	23.54	-0.25	NA	0.32	0	Myopia
-3.3125	25.175	-2.5	-1.625	0.19	0.375	Myopia
-7.625	26.58	-7	-1.25	1.32	3.75	HM
-0.75	23.55	-0.75	NA	0.04	0	Myopia
-5.6875	24.785	-5.125	-1.125	0.09	0.125	Myopia
-1.625	24.27	-1.625	NA	0.06	0.25	Myopia
-4.125	25.41	-3.5	-1.25	0.02	0	Myopia
-0.125	23.46	-0.125	NA	0.22	0.25	Myopia
-0.375	24.56	0	-0.75	0.04	0	Myopia
-5.9375	24.35	-4.25	-3.375	1.88	5.625	HM
-9.8125	27.175	-9.5	-0.625	0.27	0.875	EHM
-10.5	NA	-10.125	-0.75	NA	1.5	EHM
-8	26.885	-6.625	-2.75	0.43	1	HM
-0.8125	23.08	-0.75	-0.25	0.16	0.375	Myopia
-5.5	26.19	-5.5	NA	0.18	0.5	Myopia
-4.6875	25.245	-4.25	-0.875	0.07	0.125	Myopia
-7.0625	26.01	-6.5	-1.125	0.04	0.125	HM
-1.1875	23.71	0.125	-2.625	0.18	0.625	Myopia
-13.875	29.87	-13	-1.75	0.72	1	EHM
-5.25	26.655	-5	-0.5	0.23	0.5	Myopia
-7.6875	28.245	-6.125	-3.125	0.59	1.875	HM
-1.375	24.04	-1	-0.75	0.56	0.75	Myopia
-5.8125	25.36	-5.5	-0.625	0.06	0.125	Myopia
-6.875	28.04	-6.75	-0.5	0.28	0.25	HM
-3.75	25.075	-3	-1.5	0.11	0.25	Myopia
-2.4375	23.935	-3	1.125	0.01	0.125	Myopia
-0.375	21.415	0.125	-1	0.09	0.75	Myopia
2.375	24.45	2.25	0.25	0.12	0.5	Myopia
-3.75	24.075	-3.75	NA	0.01	0	Myopia
-11.5625	27.46	-10.625	-1.875	0.06	0.625	EHM
-10.875	29.16	-9.5	-2.75	0.12	0	EHM
-7.375	27.045	-6.75	-1.25	0.17	0.75	HM
-5	24.3	-4.375	-1.25	0.44	1.25	Myopia
-2.375	24.25	-2.25	-0.5	0.02	0.25	Myopia
-2.375	24.335	-2.75	0.75	0.07	0	Myopia
-5.875	26.555	-5	-1.75	0.97	2.5	HM
-4.625	26.29	-4.625	NA	0.38	0.75	Myopia
-0.75	25.155	-0.75	NA	0.77	1.5	Myopia
-0.875	23	-0.75	-0.5	0.06	0.25	Myopia
-1	24.9	-1	NA	0.28	0	Myopia
-1.1875	23.395	-1.125	-0.25	0.13	0.125	Myopia
-1.25	23.5	-0.875	-0.75	0.08	0.25	Myopia

-2	23.855	-1.875	-0.5	0.01	0.5	Myopia
0	22.91	0	NA	0.14	0	Myopia
-1.125	24.435	-0.5	-1.25	0.07	0.25	Myopia
-6.0625	25.57	-4.125	-3.875	0.08	0.125	HM
-3.6875	22.77	-2.875	-1.625	0.02	0.125	Myopia
-1.1875	23.725	-1	-0.375	0.05	0.125	Myopia
-3.4375	24.535	-2.875	-1.125	0.07	0.125	Myopia
0	23.23	0	NA	0.08	0	Myopia
-11.75	26.76	-9.75	-4	0.16	1	EHM
0	22.33	0	NA	0.02	0	Myopia
-2.3125	24.18	-1.75	-1.125	0.14	0.625	Myopia
-3.375	24.96	-2.375	-2	0.08	0.25	Myopia
-0.4375	22.595	0	-0.875	0.05	0.125	Myopia
-5.125	25.61	-4.5	-1.25	0.58	1.75	HM
-2.875	23.62	-1.5	-2.75	0.28	1	Myopia
-1.875	23.79	-1.875	NA	0.08	0.25	Myopia
-7.8125	26.71	-7	-1.625	0.06	0.125	HM
-6.375	25.19	-5.125	-2.5	0.02	0.5	HM
-6	25.53	-4.5	-3	0.3	0.5	HM
-5.1875	25.55	-3.5	-3.375	0.72	1.875	HM
-0.8125	23.08	-0.625	-0.75	0.04	0.375	Myopia
-5.9375	25.365	-4.875	-2.125	1.09	2.375	HM
-0.75	23.7	-0.625	-0.25	0.34	0.75	Myopia
-2.625	24.545	-2.125	-1	0.15	0.5	Myopia
-4	26.18	-3.125	-1.75	0.02	0.25	Myopia
-2.25	24.675	-2.25	NA	0.01	0	Myopia
-1.1875	23.19	-1	-0.75	0.02	0.125	Myopia
-1.75	24.475	-1.75	NA	0.25	0.5	Myopia
-8	27.04	-7	-2	0.3	0.75	HM
-7.875	25.995	-7.375	-1	0.01	0.75	HM
-2.9375	25.305	-2.5	-0.875	0.01	0.125	Myopia
-4.75	23.475	-4.625	-0.5	0.15	0.5	Myopia
-2	23.29	-1.875	-0.25	0.14	0.25	Myopia
-6.75	26.245	-6.25	-1	0.55	1.5	HM
-9.875	27.835	-9.75	-0.5	0.85	1.75	EHM
-0.5625	23.84	-0.25	-0.625	0.2	0.375	Myopia
-10.3125	27.045	-9.5	-1.625	0.35	1.125	EHM
-1.375	23.95	-1.375	NA	0.06	0.25	Myopia
-0.5625	22.625	0	-1.125	0.17	0.125	Myopia
-3.875	24.855	-3	-1.75	0.01	0.25	Myopia
-2.1875	25.28	-1.75	-0.875	0.16	0.375	Myopia
-1.875	23.53	-1.375	-1	0.22	0.25	Myopia
-3.9375	23.845	-3.375	-1.125	0.03	0.375	Myopia
-3.5625	25.135	-3	-1.125	0.41	0.875	Myopia
-1.1875	24.07	-1	-0.375	0.12	0.625	Myopia

-0.625	22.855	-0.625	NA	0.05	0.25	Myopia
-1	23.865	-1	NA	0.07	0	Myopia
-0.9375	23.225	-0.5	-0.875	0.15	0.375	Myopia
-0.125	22.465	-0.125	0	0.19	0.75	Myopia
-6.25	25.03	-5.375	-1.75	0.36	0.5	HM
-8.8125	26.85	-7.875	-1.875	0.14	0.625	HM
-5.375	25.66	-5	-0.75	0.16	0.25	Myopia
-2.5	23.74	-2.5	0	1.74	5.5	Myopia
-8.125	26.535	-7.75	-0.75	0.27	0.75	HM
-11	28.625	-9.125	-3.75	0.01	0	EHM
-8.75	27.305	-7.5	-2.5	0.85	2.5	EHM
-4.25	24.495	-4.375	0.5	0.05	0	Myopia
5.75	22.47	5.5	0.5	1.46	2	Myopia
-4.625	24.825	-4.5	-0.5	0.01	0.25	Myopia
-1.625	22.565	-1.5	-0.5	0.21	0.75	Myopia
-3.375	24.29	-2.875	-1	0.62	1.5	Myopia
-1.9375	24.045	-1.125	-1.625	0.17	0.125	Myopia
-0.875	23.61	-0.875	NA	0.1	0.25	Myopia
-3.1875	27.78	-2.875	-0.625	0.2	0.125	Myopia
-3.625	25.395	-3.375	-0.5	0.27	0.25	Myopia
-2.25	24.39	-2	-0.5	0.04	0	Myopia
-0.6875	23.085	-0.875	0.75	0.27	0.125	Myopia
-0.875	24.815	-0.875	NA	0.17	0.25	Myopia
-0.375	22.315	0	-0.75	0.17	0	Myopia
-4.375	25.97	-4	-0.75	0.4	1	Myopia
-8.0625	26.545	-7.625	-0.875	0.61	1.625	HM
-2.0625	26.645	-1.75	-0.625	0.55	1.375	Myopia
-3.625	23.665	-3.125	-1	0.09	0	Myopia
-1.3125	25.685	-0.625	-1.375	0.11	0.375	Myopia
3	21.88	3	NA	0.06	0	Myopia
-3.125	23.7	-2.125	-2	0.24	0.5	Myopia
-1.5625	23.95	-1.25	-0.625	0.08	0.375	Myopia
-3.875	25.9	-3.25	-1.25	0.1	0	Myopia
-4.5	NA	-4.125	-0.75	NA	0	Myopia
-3.5	26.23	-3.375	-0.5	0.34	0.5	Myopia
-5.375	25.91	-5.25	-0.5	0.02	0.25	Myopia
-0.875	22.41	-0.875	NA	0.02	0.25	Myopia
-0.5625	22.86	-0.25	-0.625	0.2	0.125	Myopia
-1.375	23.9	-1.375	NA	0.22	0.25	Myopia
-1.3125	24.38	-1.25	-0.25	0.38	1.125	Myopia
-5.5	25.035	-4.75	-1.5	0.01	0.25	Myopia
-5.5	25.04	-4.625	-1.75	0	0.5	Myopia
-4.125	24.88	-2.375	-3.5	0.22	0.75	Myopia
-9.5	25.735	-7.75	-3.5	0.39	1.25	EHM
-3	25.785	-2.875	-0.5	0.13	0.5	Myopia

-2.75	22.785	-1.75	-2	1.19	2.75	Myopia
-2.75	25.035	-2.75	NA	0.05	0	Myopia
-2.375	23.46	-2.125	-0.5	0.16	0.25	Myopia
-6.3125	25.03	-5.875	-0.875	0.06	0.375	HM
-4.75	24.76	-4	-1.5	0.1	0.5	Myopia
-2	24.96	-2	NA	0.04	0	Myopia
-1.875	24.105	-1.875	NA	0.17	0.75	Myopia
-5.5	24.525	-4	-3	0.07	0	Myopia
0.25	23.72	0.25	NA	0.1	0	Myopia
-0.375	23.25	-0.375	NA	0.02	0.25	Myopia
-5.6875	24.66	-5	-1.375	0.46	0.875	HM
-0.75	24.54	-0.625	-0.5	0.12	0.5	Myopia
-7	26.83	-6	-2	0	0	HM
-3.125	23.955	-3	-0.5	0.05	0.25	Myopia
-1.25	22.67	-1.25	NA	0	0	Myopia
-2.125	23.125	-1.625	-1	0.13	0.5	Myopia
-8	26.93	-7.625	-1.5	0.48	1.5	HM
-1.625	23.805	-1.5	-0.5	0.33	0.25	Myopia
-1.375	23.98	-1.25	-0.25	0.16	0	Myopia
-0.75	23.98	-0.75	NA	0.1	0	Myopia
-2.625	24.875	-2.625	NA	0.01	0.25	Myopia
-2.125	22.665	-1.875	-0.5	0.01	0.25	Myopia
-2.0625	24.27	-1.875	-0.375	0.18	0.375	Myopia
-5.375	24.285	-4.875	-1	0.07	0.25	Myopia
-4.25	24.34	-3.75	-1	0	0	Myopia
-1	25.07	-1	NA	0.06	0	Myopia
-6.25	25.29	-6.125	-0.5	0.02	0.5	HM
-2.125	25.72	-2.125	NA	0.08	0.75	Myopia
-2.25	24.85	-2.25	NA	0.02	0.5	Myopia
-0.875	23.7	-0.625	-0.5	0.02	0.25	Myopia
-6.25	25.165	-6.25	NA	0.19	1	HM
-4.125	24.735	-3.875	-0.5	0.01	0.25	Myopia
-2.0625	26.115	-1.75	-0.625	0.19	0.125	Myopia
-4.375	24.62	-4	-0.75	0.12	0.75	Myopia
-1.75	23.985	-1.75	NA	0.09	0	Myopia
-4.375	25.75	-3.75	-1.25	0.4	0	Myopia
-4.0625	24.945	-3.375	-1.375	0.43	1.125	Myopia
-0.625	22.62	-0.125	-1	0.1	0.25	Myopia
-8.25	25.12	-6.75	-3	0.3	0.25	HM
-2.625	24.305	-1.625	-2	0.49	0.25	Myopia
-6.4375	27.34	-5.875	-1.125	0.16	0.125	HM
-5.1875	25.88	-4.5	-1.375	0.8	1.625	HM
0	22.245	0	NA	0.05	0	Myopia
-4.1875	23.515	-3.875	-0.625	0.11	0.125	Myopia
-0.5	23.89	-0.5	NA	0.08	0	Myopia

-2.375	24.74	-2.375	NA	0.16	0.25	Myopia
-5.4375	25.36	-4.375	-2.125	0.58	1.125	HM
-1.125	23.85	-1.125	NA	0.02	0.25	Myopia
-3.75	25.22	-3.625	-0.5	0.02	0.5	Myopia
-2.125	24.61	-1.625	-1	0.04	0.5	Myopia
-8.0625	25.85	-7.25	-1.625	0.7	2.125	HM
-0.875	23.355	-0.875	NA	0.09	0.25	Myopia
-1.5	22.855	-1.25	-0.5	0.71	1.5	Myopia
-6.75	26.52	-6	-1.5	0.44	1	HM
-4.8125	25.605	-4.5	-0.625	0.07	0.125	Myopia
-7.375	26.315	-5.875	-3	0.39	0.75	HM
2.875	21.515	4.5	-3.25	0.01	0.5	Myopia
-5.25	24.19	-4.5	-1.5	0.5	1.5	HM
0	22.05	0	NA	0.02	0	Myopia
4.1875	22.63	5.125	-1.875	0.44	1.375	Myopia
0.1875	22.71	0.75	-1.125	0.08	0.375	Myopia
0.5	22.33	0.5	NA	0.02	0	Myopia
-1.75	23.55	-1.375	-0.75	0.1	0	Myopia
-2.3125	23.42	-1.25	-2.125	0.08	0.125	Myopia
-3.5625	24.21	-3	-1.125	0.44	0.875	Myopia
-0.375	22.86	-0.25	-0.25	0.1	0	Myopia
-0.25	22.3	-0.125	-0.25	0.04	0.25	Myopia
-5.25	24.63	-5	-0.5	0.26	0.5	Myopia
-8.25	24.235	-6.875	-2.75	0.39	1	HM
-5	24.64	-4.625	-0.75	0.06	0	Myopia
-11	27.295	-8.25	-5.5	0.41	1	EHM
-12.5625	27.205	-11.5	-2.125	0.39	1.375	EHM
0	22.705	0	NA	0.03	0	Myopia
-1.125	23.695	-1.125	NA	0.03	0.25	Myopia
-2.625	23.96	-2.375	-0.5	0.08	0.25	Myopia
-2.375	24.155	-2	-0.75	0.59	1.5	Myopia
0.25	24.85	NA	0.5	0.1	0	Myopia
0.5	22.585	0.5	NA	0.11	0	Myopia
-4.625	25.315	-5	0.75	0.17	0	Myopia
-1.3125	21.76	-1	-0.625	0.26	0.125	Myopia
-1.5	23.32	-0.625	-1.75	0.32	1	Myopia
-0.25	21.615	0.25	-1	0.17	0.25	Myopia
-3.75	25.17	-3.5	-0.5	0.04	0	Myopia
-7.25	27.675	-6.875	-0.75	0.39	1	HM
-2.375	22.8	-2.375	NA	0.04	0.25	Myopia
-1.625	23.25	-1.625	NA	0	0.25	Myopia
-1	23.835	-0.875	-0.5	0.15	0	Myopia
-1.5625	24.12	-1.375	-0.375	0.04	0.125	Myopia
-4.5	24.92	-3.875	-1.25	0.92	1.75	Myopia
-6.8125	25.42	-6.5	-0.625	0.06	0.375	HM

-6.4375	24.51	-7	1.125	0.54	1.125	HM
-2.875	24.83	-2.5	-0.75	0.08	0	Myopia
-0.3125	25.13	-0.25	-0.25	0.58	0.375	Myopia
-4.625	25.43	-3.875	-1.5	0.2	0.25	Myopia
-1.25	23.515	-1.25	NA	0.01	0	Myopia
-4.25	24.145	-4	-0.5	0.29	0.25	Myopia
0.625	22.93	0	1.25	0	0.25	Myopia
-1.875	23.79	-1.25	-1.25	0.38	0.75	Myopia
-2.5	25.525	-2.125	-0.75	0.17	0.75	Myopia
-0.75	22.615	-0.5	-0.5	0.41	0.75	Myopia
-6.3125	25.305	-5.125	-2.375	0.01	0.125	HM
-8.125	26.695	-7.875	-1	0.01	0.25	HM
-3.125	24.505	-3	-0.25	0.01	0	Myopia
-1.9375	26.38	-1.625	-0.625	0.1	0.125	Myopia
-2.5	24.165	-2.5	NA	0.05	0	Myopia
-0.0625	23.06	-0.5	0.875	0	0.125	Myopia
-0.125	23.34	-1.75	3.25	0.04	0.25	Myopia
-9.375	27.1	-8.75	-1.25	0.32	0.75	HM
-7.9375	26.035	-7.125	-1.625	0.19	0.375	HM
-1.6875	24.085	-1.625	-0.25	0.15	0.375	Myopia
-2.125	23.75	-2.125	NA	0.4	0.75	Myopia
-0.9375	23.175	-0.75	-0.75	0.09	0.375	Myopia
-1.875	23.14	-1.5	-0.75	0.18	0	Myopia
-1.625	21.8	-1.25	-0.75	0.1	0	Myopia
-6.4375	24.91	-6.125	-1.25	1.4	2.375	HM
-3.1875	24.045	-3	-0.75	0.33	0.625	Myopia
-3.25	23.965	-2.5	-1.5	0.31	0.75	Myopia
-1.375	24.175	-1.125	-0.5	0.13	0.25	Myopia
-1.375	23.62	-1.25	-0.5	0.22	0.25	Myopia
-5.375	25.525	-4.875	-1	0.37	0.75	Myopia
-8.125	27.675	-7.625	-1	0.11	0.25	HM
-11.1875	28.375	-10	-2.375	0.57	0.625	EHM
-1.25	24.245	-1	-0.5	0.23	0.5	Myopia
-1.5	23.56	-1.25	-0.5	0.18	0	Myopia
-2.75	24.555	-2.5	-0.5	0.03	0.5	Myopia
-4.3125	25.975	-4	-0.625	0.71	0.875	Myopia
-0.6625	23.86	-0.5	-0.325	0.22	1.175	Myopia
-1	23.6	-1	NA	0.02	0	Myopia

PRS_z
-0.026497448
0.330378746
-0.660663141
-0.396015304
-0.561513774
2.679466801
-0.354843853
-0.200593824
0.434284037
0.002438459
2.513770656
0.04837237
-0.001260115
0.434284037
-2.66329809
-1.09465125
2.679466801
0.406547944
-1.135937991
-1.228978533
-0.400706179
-0.053264316
0.434284037
-0.306250273
-0.640497104
0.083560531
0.330378746
-0.026497448
-0.053264316
0.434284037
-0.838507832
0.002438459
0.406547944
-0.957277898
0.083560531
-0.561513774
0.037743037
-0.838507832
-0.700734556
0.250790898
2.099530294
-0.002739545
-0.306250273

-1.228978533
-0.700734556
2.099530294
2.030490234
-0.528594672
2.744191858
0.330378746
3.756491743
-0.354843853
0.083560531
-0.640497104
0.406547944
-0.103998301
0.504704899
-1.398211766
-0.429262617
-0.990012409
0.037743037
-0.400706179
-2.660755519
-0.561513774
-0.396015304
0.434284037
-0.026497448
2.679466801
-2.568020151
-0.838507832
0.406547944
0.037743037
-0.053264316
-1.135937991
0.330378746
-0.053264316
0.002438459
-0.660663141
2.099530294
-0.990012409
0.434284037
-0.561513774
0.434284037
0.330378746
-0.396015304
-0.838507832
-0.561513774
0.04837237

-1.398211766
0.434284037
-0.200593824
-0.838507832
0.002438459
-1.010667837
0.002438459
-2.660104177
-0.561513774
-1.010667837
-0.660663141
-0.923697614
0.26503726
0.406547944
-0.053264316
-2.621816272
-0.597438875
0.362907237
0.037743037
-0.117873998
0.037743037
-0.053264316
-0.957277898
1.47816975
0.26503726
0.434284037
1.833232917
0.04837237
-0.117873998
0.362907237
-0.819691883
-2.476629576
0.171169986
0.037743037
0.26503726
0.250790898
-0.396015304
-0.103998301
2.679466801
0.434284037
4.274292196
2.679466801
0.406547944
0.330378746
0.504704899

-0.095943627
0.330378746
0.406547944
-2.664233874
-0.200593824
-2.664233874
2.928011019
-0.026497448
-0.103998301
0.406547944
-2.634799116
-2.627288112
-0.053264316
-1.010667837
0.002438459
0.037743037
0.434284037
-0.400706179
-0.640497104
-0.819691883
-2.664233874
0.083560531
-0.167596015
-1.371060434
0.083560531
-0.053264316
-0.013095554
-1.226228044
-1.135937991
-0.484764695
-2.41700239
0.072711377
-0.896315184
2.679466801
-0.001260115
0.406547944
-0.400706179
-0.013095554
-1.228978533
0.26503726
-0.026497448
0.406547944
-0.053264316
-1.09465125
2.099530294

-0.896315184
-0.013095554
-0.819691883
-0.053264316
0.002438459
-0.597438875
1.833232917
0.406547944
-1.010667837
-0.660663141
2.099530294
-0.026497448
0.856809207
0.550836211
-0.528594672
0.171169986
0.037743037
0.330378746
-0.117873998
-1.251789305
0.083560531
-0.002739545
0.434284037
-0.957277898
-0.053264316
-0.923697614
-0.990012409
0.434284037
0.171169986
-1.226228044
0.406547944
2.099530294
-1.010667837
-0.200593824
-0.026497448
-0.528594672
-0.429262617
0.434284037
0.362907237
1.857890082
-0.597438875
1.685289931
0.434284037
0.29917795
-0.896315184

0.406547944
0.083560531
-0.561513774
0.250790898
0.04837237
-0.013095554
-0.200593824
-0.838507832
0.037743037
0.362907237
-1.714269196
2.108944849
1.685289931
-2.548266828
2.099530294
-0.838507832
0.406547944
0.406547944
2.099530294
-0.838507832
3.027428706
2.108944849
0.037743037
1.685289931
-0.200593824
-0.200593824
0.002438459
-0.429262617
1.685289931
-0.002739545
1.967203511
0.037743037
-0.002739545
-0.001260115
-0.306250273
0.037743037
-1.273975123
-0.400706179
-0.013095554
1.76008333
-1.295561325
-0.400706179
-1.20538738
0.04837237
-0.026497448

0.362907237
0.330378746
-0.103998301
-0.001260115
-1.434142066
0.406547944
0.037743037
0.250790898
0.083560531
0.083560531
0.434284037
-0.306250273
-0.396015304
-1.316571895
0.037743037
1.47816975
0.037743037
0.330378746
-0.354843853
1.003973547
1.849059376
-0.200593824
0.574372597
-0.400706179
1.967203511
-0.640497104
0.434284037
0.037743037
0.250790898
-0.095943627
0.406547944
-0.095943627
-0.819691883
-0.819691883
-0.001260115
-0.429262617
-0.957277898
0.884222172
-0.743295723
-0.002739545
0.037743037
-0.053264316
0.434284037
-0.002739545
0.250790898

-0.957277898
-0.396015304
-1.20538738
-0.640497104
-0.961635774
-1.398211766
0.002438459
-0.095943627
-0.095943627
0.083560531
-0.026497448
-1.228978533
-0.838507832
0.214736645
0.037743037
-0.200593824
2.030490234
0.406547944
0.330378746
2.030490234
-0.961635774
2.099530294
0.330378746
0.406547944
-2.513291495
-1.010667837
2.099530294
-1.228978533
0.330378746
0.434284037
0.330378746
-0.640497104
0.844973767
0.330378746
0.037743037
0.406547944
-0.819691883
0.434284037
0.250790898
-0.236325083
0.434284037
-0.013095554
-0.354843853
-0.528594672
0.04837237

-0.640497104
-0.095943627
-0.597438875
0.037743037
0.362907237
-0.400706179
0.083560531
0.406547944
0.002438459
-0.819691883
0.083560531
-0.026497448
-1.434142066
-0.429262617
0.037743037
0.406547944
0.406547944
1.833232917
-0.400706179
0.434284037
0.434284037
-0.400706179
-1.226228044
0.037743037
0.037743037
-0.528594672
0.406547944
-0.838507832
-0.660663141
1.833232917
0.037743037
-0.236325083
-1.09465125
0.037743037
0.434284037
-0.396015304
0.362907237
0.504704899
-0.819691883
2.099530294
-0.561513774
-0.001260115
-0.660663141
-0.561513774
0.434284037

0.434284037
0.406547944
-0.026497448
0.406547944
0.04837237
0.250790898
-0.200593824
-0.026497448
-0.819691883
0.406547944
1.857890082
0.04837237
0.301350539
-0.053264316
-0.354843853
-0.429262617
0.002438459
0.037743037
-1.010667837
-0.001260115
0.574372597
0.04837237
-0.001260115
-0.236325083
-1.371060434
-0.429262617
-0.026497448
-0.001260115
-0.597438875
-2.550894501
0.406547944
-0.660663141
-0.896315184
2.030490234
0.434284037
0.26503726
-0.400706179
-0.095943627
0.037743037
-0.306250273
-0.400706179
0.406547944
-0.819691883
0.04837237
0.037743037

-0.026497448
-0.660663141
4.274292196
-0.400706179
0.083560531
-0.053264316
-0.053264316
-0.961635774
0.406547944
-0.200593824
2.679466801
0.250790898
0.083560531
-0.013095554
-1.316571895
0.406547944
-1.010667837
0.171169986
0.434284037
0.037743037
-0.957277898
0.434284037
-0.026497448
0.002438459
-0.053264316
-0.103998301
-0.002739545
-0.167596015
-0.640497104
0.406547944
-0.001260115
0.406547944
-0.200593824
0.037743037
-0.990012409
-0.001260115
0.574372597
-0.561513774
0.037743037
-0.200593824
-0.200593824
0.250790898
-0.001260115
-0.053264316
-2.642449948

0.434284037
0.504704899
1.833232917
1.833232917
-0.23874754
-0.236325083
0.083560531
-0.002739545
0.434284037
0.406547944
0.434284037
-0.103998301
-0.026497448
0.26503726
-2.013905472
0.330378746
-0.990012409
0.330378746
-0.002739545
-0.002739545
0.362907237
2.099530294
1.47816975
-0.354843853
-0.561513774
3.9418098
0.26503726
-0.23874754
-0.640497104
0.434284037
-0.002739545
0.434284037
0.04837237
-0.002739545
-0.053264316
0.083560531
0.002438459
2.099530294
0.362907237
-0.838507832
-0.429262617
-0.095943627
0.434284037
-1.18697964
-0.001260115

-0.306250273
0.083560531
2.030490234
2.679466801
-1.251789305
-0.167596015
-0.957277898
-0.095943627
0.511608904
0.037743037
0.434284037
-1.337029555
0.002438459
0.037743037
-0.561513774
0.002438459
0.511608904
-0.660663141
-0.306250273
-0.838507832
-0.838507832
-0.400706179
0.574372597
-0.095943627
0.574372597
0.434284037
1.47816975
-0.396015304
0.250790898
-0.819691883
-0.396015304
1.716357959
-2.66329809
0.406547944
-0.400706179
-0.838507832
2.030490234
0.04837237
