# Supplementary material for: Integrating Polygenic Risk and Ocular Phenotyping Reveals an Axial-Length–Dominant Mechanism in High and Extreme High Myopia
Source: Ophthalmol Sci. 2026 Apr 15;6(6):101194. doi: 10.1016/j.xops.2026.101194 (PMC13195619; doi:10.1016/j.xops.2026.101194)
Supplement: Table S4 [file mmc4.pdf]

Table S4. Shared or concordant loci between HM and EHM stratified GWAS

Chromosome	Position	Nearest gene	HM P value	EHM P value	Consistency note
21	10752117	TPTE	$<5\times10^{-8}$	$<5\times10^{-8}$	Genome-wide significant in both analyses
12	85369026	LINC02820	$<5\times10^{-8}$	$<5\times10^{-8}$	Shared locus in HM and EHM
2	47264023	LOC107985882	$<5\times10^{-8}$	$<5\times10^{-8}$	Shared locus
6	49348423	MMUT	$<5\times10^{-8}$	$<5\times10^{-8}$	Shared locus
10	55450955	PCDH15	$<5\times10^{-8}$	$<5\times10^{-8}$	Shared locus
3	188512558	LPP	$<5\times10^{-8}$	$<5\times10^{-8}$	Shared locus
8	16805421	PLPP3	$<5\times10^{-8}$	$<5\times10^{-8}$	Shared locus
5	58495042	PDE4D	$<5\times10^{-8}$	$<5\times10^{-8}$	Shared locus
16	60621345	RBFOX1	$<5\times10^{-8}$	$<5\times10^{-8}$	Shared locus
7	122913812	MGAT4C	$<5\times10^{-8}$	$<5\times10^{-8}$	Shared locus
1	186472193	MACF1	$<5\times10^{-8}$	$<5\times10^{-8}$	Shared locus
3	142995084	LSAMP	$<5\times10^{-8}$	$<5\times10^{-8}$	Shared locus
